# Questionnaires Used to Explore the Perspectives of Parents and Health Professionals on Young Children’s Use of Technology: Systematic Review

**DOI:** 10.2196/84712

**Published:** 2026-06-11

**Authors:** Charlotte Lund Rasmussen, Ivan Pui Hung Au, Danica Hendry, Amber Beynon, Sarah Stearne, George Thomas, Kate Mannell, Lisa Kervin, Susan Edwards, Courtenay Harris, Leon Straker, Juliana Zabatiero

**Affiliations:** 1 School of Allied Health, Curtin University, Kent Street, Bentley Western Australia, Perth, 6102, Australia, 61 8 9266 3600; 2 ARC Centre of Excellence for the Digital Child, Brisbane, Australia; 3 School of Human Movement and Nutrition Sciences, Health and Wellbeing Centre for Research Innovation, The University of Queensland, Brisbane, Australia; 4 Faculty of Arts and Education, Deakin University, Melbourne, Australia; 5 Faculty of Education, Monash University, Clayton, Victoria, Australia; 6 Institute for Learning Sciences & Teacher Education, Australian Catholic University, Melbourne, Australia

**Keywords:** measurement tools, questionnaire validation, early childhood, digital parenting, digital age

## Abstract

**Background:**

Technology is integrated into many children’s daily lives, with parents’ and health professionals’ perspectives shaping children’s technology use. Measuring and understanding these perspectives are essential for developing strategies for supporting adults in decision-making that help children thrive in a digital world.

**Objective:**

This systematic review aimed to investigate the psychometric properties of questionnaires used to assess parents’ and health professionals’ perspectives on young children’s use of technology related to health, well-being, and development. The secondary aim was to synthesize findings on these perspectives.

**Methods:**

Peer-reviewed papers published between January 2010 and September 2024 were identified through searches in 7 electronic databases. Studies were included if they examined parental or health care professionals’ perspectives on technology use among children aged birth to 5 years. Two reviewers (CLR and IPHA) independently conducted the data extraction and study quality assessment. Reported psychometric properties of the questionnaires were synthesized. Deductive thematic analysis was used to explore the content focus of the questionnaire used in the included studies and synthesize the reported perspectives.

**Results:**

In total, 85 studies were included, all involving parents. No study investigated health professionals’ perspectives. The methodological quality of the studies was generally low, with 62 studies scoring below the threshold for acceptable quality. In total, 52 studies reported psychometric properties of the questionnaires used, of which, only 15 studies reported more than 1 measure of validity or reliability. A total of 75 studies reported participants’ perspectives on children’s technology use. Findings revealed that parents generally supported the role of digital devices in enhancing learning but expressed concerns about potential negative impacts on children’s physical health, emotions, and behaviors.

**Conclusions:**

Parents’ perspectives on children’s technology use were frequently assessed through questionnaires, though the validity of these questionnaires was often poor, with limited psychometric testing. Parental perspectives were mixed with educational benefits being recognized, while countered with concerns about the impact on children’s physical health and development. High-quality questionnaires are needed to generate stronger evidence informing strategies to support families in technology use decision-making with and for children.

## Introduction

Technology is now an integrated part of many children’s daily lives [1-3], encompassing both screen-based (eg, tablets, smartphones, and computers) and nonscreen-based (eg, smart toys and interactive learning tools) devices [4]. Recent survey data indicate a substantial increase in young children’s technology use. For example, data from the United States suggest that children younger than 8 years of age spend ~2.5 hours per day on screen devices [3], while Australian children aged 2‐6 years are estimated to spend ~25.9 hours per week [1]. The rise in young children’s technology use has led to an increase in research, assessing its potential benefits and harms for children’s health, well-being, and development [5]. Evidence suggests that technologies can enhance learning, language development, creativity, and support social connections [6-9]. However, excessive screen time has been associated with negative health effects, including sleep disturbances and obesity [10,11]. Moreover, exposure to inappropriate content has been linked to emotional and behavioral challenges such as attention issues and anxiety [7]. These mixed results highlight the complexity of technology’s role in the health, well-being, and development of young children (birth to 5 years), and how factors such as content, context, and duration of use can influence technology’s role [12].

Children’s interaction with technology happens within a system, whereby multiple factors influence how children use technology [13,14]. One of these factors is the perspectives (ie, beliefs, attitudes, and viewpoints) of key adult figures in children’s lives, including parents, educators, and health professionals. Parents are often primary mediators of children’s technology use, as they play a central role in shaping technology use by setting rules, providing supervision, and coengagement. Thus, parent perspectives may influence children’s use of technology and its impact on their health, well-being, and development [15,16]. Research suggests that parents hold mixed perspectives on children’s technology use, mirroring broader public debates [17]. In total, 3 systematic reviews found that parents hold concerns about the negative impacts of screen time on children’s physical health, social skills, exposure to inappropriate content, and development of addiction [18-20]. In contrast, the review by Choy et al [19] also found that parents perceived technology to be beneficial for learning, creativity, digital literacy, and social-emotional development. While these findings provide some insights into the complexity of parents’ perspectives toward technology, the reviews by Chong et al [18] and Visier-Alfonso et al [20] only considered qualitative studies [18,20]. Moreover, Chong et al [18] did not investigate positive views on screen time, and the reviews by Visier-Alfonso et al [20] and Choy et al [19] only included studies that reported on technologies used to support physical activities or were internet-based.

Health professionals, such as medical doctors, child health nurses, and allied health professionals, are a trusted information source [16] who provide recommendations on healthy technology use, which parents often rely upon to make informed decisions about their children’s digital engagement. However, their perspectives on children’s technology use have not been systematically investigated and synthesized. Educators play a crucial role in enhancing learning experiences and digital literacy within the early learning and school environment and in supporting parents [15]. The perspectives of educators on children’s technology use have been extensively discussed [15,21,22] and appear to reflect the broader community and parent perspectives. Specifically, educators have been found to hold positive views on the use of technology to support learning and engagement while also expressing concerns about exposure to inappropriate content and overuse. Given the substantial body of research on this topic, educators’ perspectives will not be considered in this paper.

The perspectives of parents and health professionals may play an important role in shaping children’s engagement with technology. For example, negative parental views on technology have been associated with more restrictive mediation strategies, whereas positive parental views have been linked to greater integration of technology into children’s lives [23]. Likewise, health professionals with a positive view on technology may encourage parents to actively engage with their children’s technology use, whereas those with a negative view may highlight the potential health risks associated with technology.

Perspectives can be captured through both qualitative and quantitative methods, such as interviews and questionnaires. While qualitative methods can provide a detailed understanding of perspectives, they are typically limited in sample size and generalizability. Given the low participant burden of questionnaires [24], this method enables large-scale data collection, which is essential for comparison of perspectives across different population groups. While questionnaires are useful tools, their validity is crucial for ensuring accurate and reliable high-quality evidence. However, no study has systematically investigated questionnaires that have been developed and used for assessing adults’ perspectives on children’s technology use and the validity of these tools. Thus, the focus of this study was on questionnaires. Having valid questionnaires available for capturing such perspectives can aid in developing evidence-based strategies for supporting adults in moving toward perspectives and practices that help children to thrive in a digital world.

Therefore, the primary aim of this systematic review was to investigate the psychometric properties of questionnaires that have been used to assess parents’ and health professionals’ perspectives on young children’s use of technology related to health, well-being, and development. A secondary aim was to summarize the perspectives of parents and health professionals on young children’s use of technology as measured by these tools.

## Methods

### Study Protocol

The study protocol was prospectively published on Open Scientific Framework [25]. This systematic review adhered to the PRISMA (Preferred Reporting Items for Systematic Review and Meta-Analyses) [26] (Checklist 1).

### Ethical Considerations

This systematic review was exempted from ethics or institutional review board approval, as it did not involve human participants, and ethics approval was not required to review published or publicly reported literature.

### Search Strategy

Searches were conducted in the following electronic databases: CINAHL, Embase, MEDLINE, ProQuest, PsycInfo, Scopus, SPORTDiscus, and Web of Science (Core Collection). These databases were selected to ensure coverage across multiple research disciplines relevant to the review topic, that is, health and medical sciences (MEDLINE and Embase), psychology and behavioral sciences (PsycInfo), nursing and allied health (CINAHL), as well as multidisciplinary sources (Scopus, Web of Science, and ProQuest). The search targeted papers published between January 1, 2010, and September 1, 2024, to capture contemporary technology and was conducted using a combination of search terms relating to adults’ perspectives, technology use, target population (ie, parents, health professionals, and children), and method (ie, questionnaire). A full list of search strategies by databases is provided in Multimedia Appendix 1. Additionally, reference lists of included papers were searched for additional relevant studies.

### Selection of Studies

All retrieved studies were exported into EndNote (version 20; Clarivate Analytics). Two reviewers (CLR and IPHA) independently screened titles and abstracts to identify potentially relevant papers using Research Screener, a software system that uses artificial intelligence to iteratively learn from screening decisions, reducing the need to review irrelevant papers [27]. Following title and abstract screening, full text screening of papers was completed independently by the same 2 reviewers. Any researcher disagreement on the eligibility of a particular study was resolved through discussion, with a third reviewer (JZ) to resolve discrepancies.

### Inclusion Criteria

Studies were included if they explored parents’ and health professionals’ perspectives on young children’s (aged birth to 5 years) technology use, including screen- and nonscreen-based devices, using questionnaires (eg, self-report survey instruments and diaries). Studies were included if they reported the use of a questionnaire relevant to the review aim, regardless of whether full details on the questionnaire and its development were reported. Only original peer-reviewed papers, published in English, were considered.

### Exclusion Criteria

Review studies, expert opinions, editorial letters, and studies that focused on children with chronic health conditions (eg, cancer, diabetes, and epilepsy) were excluded.

### Quality Assessment of Included Studies

The methodological quality of the included studies was assessed using the Quality Assessment Checklist for Survey Studies in Psychology (Q-SSP) [28]. The Q-SSP was selected, as it was specifically developed to evaluate the quality of survey-based studies, which aligned with the focus of this review. The Q-SSP was developed through a multistage process, which included reviewing existing quality assessment tools, establishing expert consensus on selected items, and refining the instrument based on validity assessments. The Q-SSP is a 20-item checklist that evaluates survey quality across 4 domains: introduction (4 items), participants (3 items), data (10 items), and ethics (3 items). Each item has the following response categories: yes (met criteria), no (did not meet criteria), not stated clearly, or not applicable. For each study, an overall quality score and a quality score for each domain were calculated as the percentage of “yes” out of the total applicable items. Depending on the number of applicable items, studies were required to achieve a set threshold score to be rated as “acceptable” in quality, while studies with scores less than this threshold were deemed to have “questionable” quality. Specifically, studies were classified as having “acceptable” methodological quality if they achieved an overall score ≥73% in accordance with the threshold defined in the Q-SSP [28]. The following were used as thresholds for “acceptable” quality for each domain as recommended: ≥75% for introduction, ≥67% for participants, ≥70% for data, and ≥100% for ethics. Of note, item 19 (“Were participants debriefed at the end of data collection?” ethics domain) was considered as not applicable, as debriefing could be argued as being impractical and that the data collection was harmless, given that all included studies were based on questionnaires. Study quality was independently assessed by 2 reviewers (CLR and IPHA), and any discrepancies were resolved by discussion without the requirement of a third reviewer.

### Data Extraction and Synthesis of Studies Included in the Review

The following data were extracted from the included studies: study characteristics, questionnaire characteristics, questionnaire items, and perspectives from study participants. Study characteristics included author, year of publication, country where the study was completed, country setting (ie, country income classification), study aim and design, sample sizes, sample characteristics, and recruitment strategy. Sample characteristics included age, gender identity, and relationship to the child. Information on the following questionnaire details was extracted: questionnaire details (ie, title, number of items, and administration format), questionnaire development, number of items and scoring details, and questionnaire psychometric properties.

Reported psychometric properties of the questionnaires were descriptively synthesized. When available, this included evidence of validity (ie, face validity, content validity, construct validity, and criterion validity) and reliability (ie, internal consistency and test-retest reliability). Questionnaire items related to adults’ perspectives on children’s technology use and the reported perspectives were extracted from each study and entered into NVivo (version 14; QSR International) [29] to facilitate coding and identification of common themes related to the focus of the questionnaires and reported perspectives. All data were extracted independently by 2 reviewers (CLR and IPHA) and cross-checked for any discrepancies, which were resolved without requiring a third reviewer.

A 2-level coding framework was developed to systematically assess (1) technology type, that is, technologies the questionnaire referred to, and (2) questionnaire content focus, that is, the focus of the items within the questionnaire regarding adults’ perspectives on children’s technology use. Following this framework, each questionnaire was first coded based on the technology type it referred to using the following codes (level 1): applications, computer, digital media or digital device, gaming, mobile touch screen devices, no specific hardware, screen time, and television. Next, a deductive thematic analysis [30] was used to investigate the content focus of each item and the reported perspectives, which were classified into 4 key dimensions based on the child-technology interaction model [14] to facilitate reader understanding:

- Child: characteristics of the child, including demographic factors and individual capabilities (eg, age, physical health, and cognition);
- Other people: the role of family members or other social influences, such as peers and siblings, in children’s technology use (eg, family interaction and coviewing);
- Tasks: the purpose for which the technology is used (eg, learning and entertainment); and
- Technology: the nature of the technology involved (eg, software and content).

## Results

### Study Inclusion

**Figure 1.**
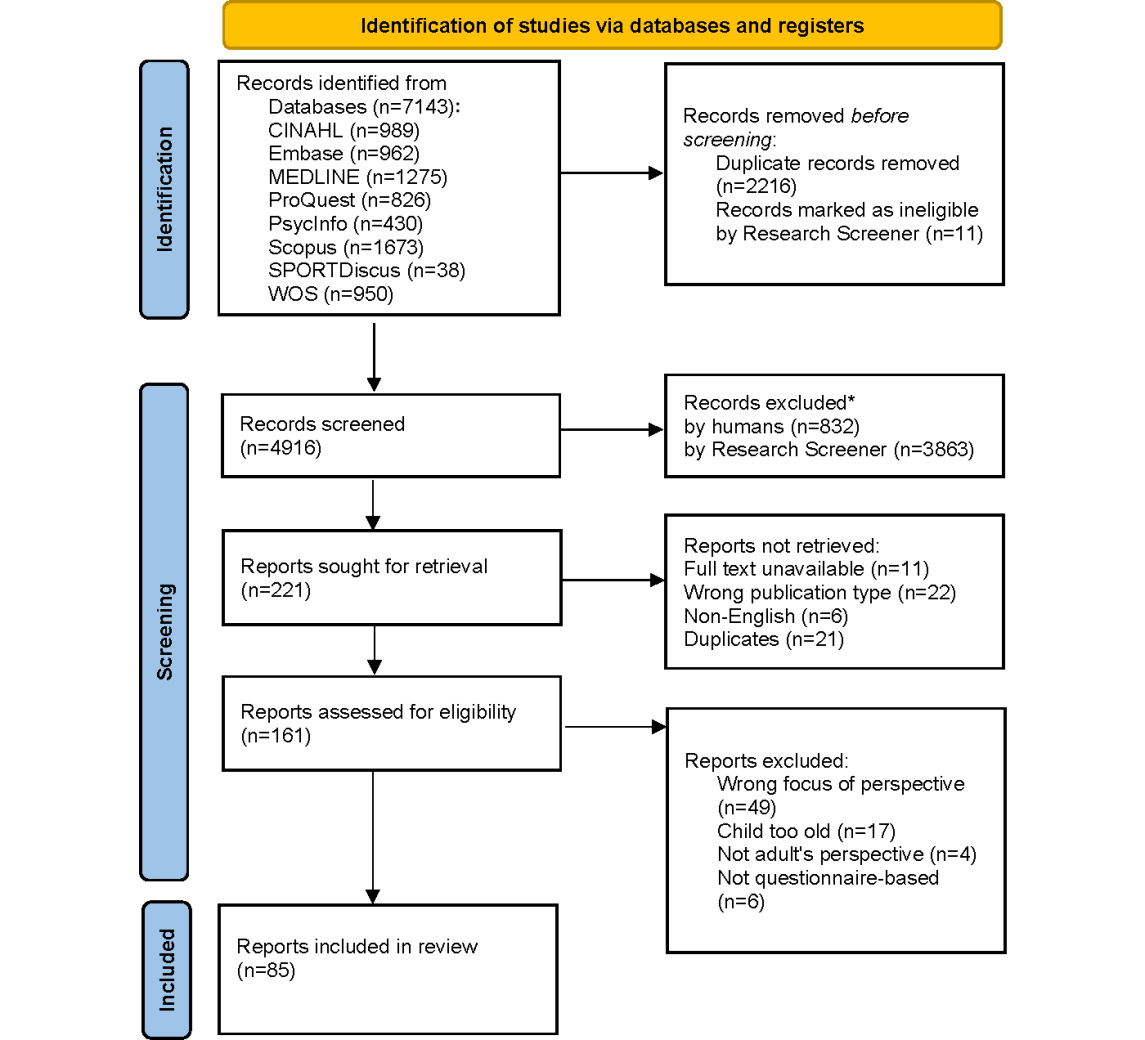
PRISMA flowchart of included studies. *Screening took place using “Research Screener,” an artificial intelligence screening software. Four records were considered ineligible by Research Screener due to a lack of abstracts. In total, 21 rounds of screening (50 in each round) took place before the screeners determined that no further useful papers were being shown. PRISMA: Preferred Reporting Items for Systematic Reviews and Meta-Analyses.

Figure 1 shows the PRISMA flowchart of search results and study exclusions. A total of 7143 records were identified based on the initial database search, of which 85 studies met the inclusion criteria for this review.

### Quality Assessment of Included Studies

Results of the quality assessment are presented in Table S1 in Multimedia Appendix 2 [31-115]. The average overall quality score was 61% (SD 15%; range 32%‐100%), with 62 studies scoring below the threshold for acceptable quality (ie, 73%), indicating questionable quality. Generally, studies had higher quality scores for introduction, as 84 studies scored above the threshold for acceptable quality (overall mean 98%, SD 8%; range 50%‐100%). In total, 47 and 17 studies, respectively, had acceptable quality related to information about study participants (mean 49%, SD 26%; range 0%‐100%) and data (mean 49%, SD 19%; range 10%‐100%). A total of 49 studies provided acceptable quality information related to ethics (mean 60%, SD 48%; range 0%‐100%).

### Characteristics of Included Studies

The characteristics of included studies are provided in Table 1. Studies were published between 2012 and 2024, with most studies published in 2022 (11/85, 13%) and 2023 (22/85, 26%). Only 3 studies aimed to explicitly develop and validate questionnaires for measuring parents’ perspectives on children’s use of technology [33,39,112]. Among the remaining studies, most studies aimed to investigate adults’ perspectives of children’s use of technology (48/85, 56%) and/or determinants of children’s technology use (19/85, 22%).

**Table 1.** Characteristics of the included studies.

Author, year, country, country setting^a^	Aim of study	Study design and measurement type	Participants’ characteristics (sample size; relationship to child; gender^b^; age)	Recruitment strategy
Akyol (2022) [31], Turkey, UMIC^c^	To investigate the effect of an online education program on parents’ views on their children’s technology use and online education.	Unclear if results are based on cross-sectional or longitudinal measurements. Mixed method (questionnaire and case study).	20; parents; 50% women; age NR^d^.	NR.
Aladé and Donohue (2023) [32], United States, HIC^e^	To investigate the relationships between children’s at-home technology use, their parents’ attitudes toward technology, and their in-school tablet use.	Longitudinal. Mixed method (questionnaire and case study).	258; parents; 78% women; age NR.^d^	Parents were recruited from kindergartens that participated in a project where urban schools were given a tablet to use in the classroom.
Al-Balushi and Al-Shihi (2016) [33], Oman, HIC	To investigate parents’ awareness of the risks of mobile devices on children and their happiness about their children’s mobile device use.	Cross-sectional. Mixed method (explorative case study and questionnaire validity study).	10; parents; gender NR; age NR.	NR.
Alkalash et al (2023) [34], Saudi Arabia, HIC	To investigate parents’ knowledge, attitudes, and practices of regulating screen exposure among their 6-year-old children in Saudi Arabia.	Cross-sectional. Questionnaire-based.	451; parents; 64% women; 35% aged between 25 and 34 years.	NR.
AlSamhori et al (2025) [35], Jordan, UMIC	To investigate parents’ awareness of their 4‐ to 10-year-old children’s electronic device use and their perspectives on how screen time affects children’s behavior in Jordan.	Cross-sectional. Questionnaire-based.	807; parents; gender NR; mothers’ mean age was 35 (SD 5) years, fathers’ mean age was 39 (SD 6) years.	Parents were recruited through various methods, including social media platforms and the distribution of the questionnaire in paper format at different locations in Jordan.
Amzalag (2021) [36], Israel, HIC	To investigate (1) parents’ perceptions about digital learning games and 21st century skills and (2) parents’ attitudes toward digital learning games instead of traditional homework.	Cross-sectional. Mixed method (questionnaire and semistructured interview).	198; parents; 71% women; age NR.	NR.
Arippin et al (2023) [37], Brunei Darussalam, HIC	To investigate parents’ knowledge, attitude, practice, and experience of the screen time spent by their children younger than 5 years of age at home and to identify the common screen-based devices used, content type, and level of screen time spent by the children.	Cross-sectional. Questionnaire-based.	113; parents; 91% women; age NR.	Parents were recruited through health care facilities in 4 districts in Brunei Darussalam.
Asplund et al (2015) [38], United States, HIC	To investigate (1) the rates of adherence to the American Academy of Pediatrics (AAP) screen time guidelines among Latino parents, (2) parental attitudes toward childhood electronic media use, and (3) the associations of child BMI, child and parent demographic characteristics, and household media environment with adherence to AAP screen time guidelines and with parents’ attitudes toward childhood media use.	Cross-sectional. Questionnaire-based.	302; parents; gender NR; 47% younger than 30 years.	Families with an appointment at a clinic offering a nutrition program for women, infants, and children were contacted by study staff and asked to complete a survey.
Balaban Dağal and Bayındır (2019) [39], Turkey, UMIC	To develop a scale, “Effects of Digital Games in Early Ages Scale,” which aimed to evaluate the effects of digital games on children during early childhood according to parental perception.	Cross-sectional. Questionnaire validation study.	777; parents; gender NR; age NR.	Families were recruited from 14 randomly selected schools with lower, middle, and upper socioeconomic status.
Bansal et al (2023) [40], India, LMIC^f^	To investigate the exposure of media and its influence among children.	Cross-sectional. Questionnaire-based.	405; parents; 63% mothers; age NR.	NR.
Barmomanesh et al (2017) [41], New Zealand, HIC	To investigate the use of touch screen devices in particular tablets or smartphones among 0‐ to 5-year-old children and to ascertain parents’ insights toward the use of these types of technologies by their children.	Cross-sectional. Questionnaire-based.	166; parents; 95% women; age NR.	Parents were recruited through a parenting Facebook page. An announcement containing a direct link to the survey was also sent by a manager at an education center to invite parents for participation in the survey.
Beyens and Eggermont (2014) [42], Belgium, HIC	To investigate predictors and consequences associated with the use of television as a “babysitter” for young children.	Cross-sectional. Questionnaire-based.	844; parents; 88% women; mean age 36 (SD 4.70) years.	Parents were recruited via invitation letters sent to 47 kindergartens and childcare centers.
Bleakley et al (2013) [43], United States, HIC	To investigate determinants and beliefs associated with parents’ intention to limit their children’s television viewing.	Cross-sectional. Based on telephone surveys.	516; parents; 67% women; mean age of 41 (SD 9.7) years.	Parents were selected using a random digit dialing from a publicly available list of households with a greater likelihood of containing a child between 3 and 16 years.
Boonmun et al (2023) [44], Thailand, UMIC	To investigate the effects of a training program on parents’ planned behaviors and screen time reduction of their children.	Quasi-experimental study. Questionnaire-based.	67; parents; gender NR; mean ages were 27 years in the control group and 30 years in the intervention group.	Parents were recruited from 2 childcare centers in Thailand.
Bourha et al (2024) [45], Greece, HIC	To investigate parental perspectives on using digital technologies and the use of technology-enhanced toys among their 1‐ to 4-year-old children.	Sequential explanatory study (dual phase: pre- and postinteraction with technology-enhanced toys). Questionnaire-based.	Prequestionnaire: 78; parents; 81% women; 59% were between 30 and 40 years. Postquestionnaire: 59; parents; 86% women; 66% were between 30 and 40 years.	Parents were recruited from 5 childcare centers.
Brauchli et al (2024) [46], Switzerland, HIC	To investigate the roles of parenting stress and attitudes toward children’s screen time throughout early childhood.	Longitudinal. Questionnaire-based.	462; parents; 93% women; mean ages were 36‐37 (SD 4) years across 4 measurement time points.	Parents were recruited through newsletters from nonprofit organizations, posts in social media, advertisements in parenting magazines, and flyers distributed at daycare centers, pediatricians, and parental counseling centers.
Brown et al (2023) [47], United States, HIC	To investigate (1) parental knowledge and educational interests, (2) parental motivation or self-efficacy, and (3) parental beliefs about digital media use and determine how these vary based on children’s age.	Cross-sectional. Questionnaire-based.	611; parents and grandparents; 77% women; mean age of 36.2 years (95% CI 35.4‐37.1).	Parents from one of the pediatric practices associated with a statewide pediatric clinical trials and research network for their children’s wellness appointment were offered to participate in the study by filling out a paper survey containing a description of the project.
Cardy et al (2023) [48] Canada, HIC	To investigate the patterns and purposes of technology use among autistic children compared to nonautistic children, explore how technology use affects children’s well-being, and examine parents’ attitudes toward children’s technology use.	Cross-sectional. Questionnaire-based.	611; parents; gender NR; age NR.	Parents were recruited via hospital social media channels, newsletters and email distribution lists, and word-of-mouth.
Carson and Janssen (2012) [49], Canada, HIC	To investigate the influence of intrapersonal, interpersonal, and physical environment factors within the home setting on screen time among 0‐ to 5-year-old children.	Cross-sectional. Questionnaire-based.	746; parents; 92% women; age NR.	Parents with children ≤5 years were recruited from licensed childcare centers and public health or community programs.
Carson et al (2013) [50], Canada, HIC	To investigate the proportion of 0‐ to 4-year-old children meeting the new Canadian Sedentary Behaviour Guidelines for the Early Years and to describe parental attitudes toward and barriers to reducing screen time.	Cross-sectional. Questionnaire-based.	657; parents; gender NR; age NR.	Parents of preschool children were recruited from childcare centers and public health or community programs.
Chattha et al (2021) [51], Pakistan, LMIC	To investigate daily screen time among 2‐ to 5-year-old children along with predisposing factors, quality of screen time, and parental perceptions of their children’s screen time.	Cross-sectional. Mixed method (interview and questionnaire-based).	200; parents; gender NR; age NR.	Parents were recruited from general outpatient departments and pediatric outpatient departments of hospitals.
Chia et al (2022) [52], Singapore, HIC	To investigate the relationship between parent and child digital media use and to compare the characteristics of the top and bottom quartiles of children’s daily digital media use.	Cross-sectional. Questionnaire-based.	1481; parents; 79% women; age NR.	Parents of children aged 2‐5 years were recruited from preschools via invitation letters sent by the preschool operator.
Chen and Tu (2018) [53], Taiwan, HIC	To investigate the impact on parents’ attitudes toward internet use and their expectations of preschools to use internet-related applications.	Cross-sectional. Questionnaire-based.	483; parents; 71% women; 67% were aged 31‐40 years.	NR.
Cingel and Krcmar (2013) [54], United States, HIC	To investigate how parent and child demographics, parental media-use motives, parental subjective norms, and parental attitudes toward preschool media use relate to actual media exposure among children.	Cross-sectional. Questionnaire-based.	168; parents; 85% women; age NR.	Parents were recruited from daycare centers, organized parent groups, and via an online snowball sample, where a link to an online version of the survey was sent to a number of parents who forwarded the link to others.
Covolo et al (2021) [55], Italy, HIC	To investigate (1) adults’ opinions about mobile device use among 0‐ to 5-year-old children, (2) if being parents of young children increased risk perception, and (3) the relationship between risk perception and parental attitudes to their children’s mobile device use.	Cross-sectional. Questionnaire-based.	3115; parents; 86% women; mean age of 37.2 (SD 10.5) years.	Participants were recruited using a snowball sampling method by sharing a link of an online survey via social media and distributing the link through WhatsApp and Facebook groups dealing with children’s health.
Dardanou et al (2020) [56], Norway, Portugal, and Japan, HIC	To investigate (1) parents’ views on 0‐ to 3-year-old children’s use of touchscreen devices at home in Norway, Portugal, and Japan and (2) how dominant cultural discourses influence parents’ perceptions and concerns regarding their 0‐ to 3-year-old children’s use of touchscreen devices.	Cross-sectional. Questionnaire-based.	552; parents; gender NR; age NR.	NR.
Dong et al (2022) [57], China, UMIC	To investigate (1) the average levels of young children’s digital literacy and multimodal practices in central China, (2) the latent profiles of digital families in central China, and (3) the predictors of young children’s digital literacy and multimodal practices in central China.	Cross-sectional. Questionnaire-based.	1953; parents and grandparents; 80% women; 39% aged between 31 and 35 years.	Online invitations were sent to all parents of children aged 0‐8 years in a province in central China using an online survey platform. Furthermore, kindergarten principals, teachers, educators, and researchers advertised the online survey via social networks.
Eales et al (2021) [58], United States, HIC	To investigate (1) how children use screen media differently pre- versus post–COVID-19 and parent perceptions of their child’s media use and (2) the parent and child factors that moderate trajectories of change in screen media use.	Longitudinal. Questionnaire-based.	129; parents; 98% women; mean age of 39.4 (SD 4.34) years.	Parents of children aged 2‐11 years were randomly selected from a city-wide participant pool and sent an email with a survey link. Parents who indicated that they were willing to be recontacted were sent a follow-up mail with an invitation to complete another survey.
Ebbeck et al (2016) [59], Singapore, HIC	To investigate (1) which technological devices are used by 0‐ to 7-year-old children in Singapore, (2) the time duration spent on using different technological devices by 0‐ to 7-year-old children in Singapore, and (3) parents’ views of risks and benefits of their children accessing the emerging touch screen devices.	Cross-sectional. Questionnaire-based.	1058; parents; 72% women, 69% aged between 31 and 40 years.	Information letters were sent to parents or caregivers by email and hard copy to principals of 34 childcare centers.
Fan et al (2023) [60], China, UMIC	To investigate the parental perspectives of 2‐ to 6-year-old children’s use of short-video mobile apps.	Cross-sectional. Mixed method (questionnaire and interviews).	266; parents; 68% women; 73% were aged between 30 and 40 years.	Parents were recruited from an online survey service.
Farima et al (2023) [61], Moldova, UMIC	To investigate parents’ knowledge, attitudes, and practices regarding the use of electronic devices by preschool children.	Cross-sectional. Questionnaire-based.	422; parents; 87% women; 63% were aged between 26 and 35 years.	NR.
Garcia-Conde et al (2020) [62], Spain, HIC	To investigate how parental attitudes toward sleeping, screen use, and feeding their child influence the child’s body mass index.	Cross-sectional. Questionnaire-based.	908; parents; 84% women; age NR.	Parents were recruited from 21 primary schools in the main cities.
Gjelaj et al (2020) [63], Kosovo, UMIC	To investigate preschool teachers’ and parents’ attitudes and practices regarding digital technology use during preschool education.	Cross-sectional. Mixed method (questionnaire filled out by parents and teachers, and interviews).	100; parents; gender NR; age NR.	Parents and teachers were recruited from preschools by random sampling.
González-Sanmamed et al (2023) [64], Spain, HIC	To investigate families’ perspectives on their children’s use of mobile devices.	Cross-sectional. Questionnaire-based.	241; parents; 73% women; mean age was 39 (SD 7) years.	Parents were recruited from schools.
Grané et al (2023) [65], Spain, HIC	To investigate how and in what way families’ perceptions and beliefs about digital technologies modulate children’s use of screens at home.	Cross-sectional. Mixed method (questionnaire and interview).	46; parents; 76% women; age NR.	NR.
Griffith et al (2025) [66], United States, HIC	To investigate parents’ experiences with remote schooling and technology-facilitated homework and how family characteristics, parent attitudes toward screen media, and parent digital skills relate to experiences with remote schooling and attitudes toward the use of digital technology for homework.	Cross-sectional. Questionnaire-based.	822; parents; 82% women; mean age was 36 (SD 9) years.	Parents were recruited from online panels via web-based advertising, customer loyalty web portals, and targeted email lists.
Halpin et al (2021) [67], Australia, HIC	To investigate relationships between parenting style, self-efficacy, child behavior, parent distress, and screen time in young children, and to identify important variables predicting self-efficacy for managing children’s screen time and actual screen use in young children.	Cross-sectional. Questionnaire-based.	106; parents; 95% women; mean age 33.76 (SD 5.35) years.	Parents were recruited via advertisements on social media, parenting forums and websites, and emails sent out to childcare centers.
Hamilton et al (2016) [68], Australia, HIC	To investigate the decision-making process and the beliefs underpinning parents’ decisions to ensure that their child’s screen time is limited according to the guidelines developed by the Department of Health, Australia.	Longitudinal. Mixed method (questionnaire and telephone interview).	207; parents; 67% women; mean age of 36.38 years.	Participants were recruited via online sources, face-to-face recruitment, and through their child’s daycare facility.
Hatzigianni et al (2014) [69], Australia, HIC	To investigate parents’ beliefs about how and why their young children use computers.	Longitudinal. Questionnaire-based.	51; parents; gender NR; age NR.	Parents were recruited from primary schools and childcare centers, where the teachers were willing to participate in a computer intervention.
Howie et al (2020) [70], Australia and United States, HIC	To investigate the reliability and validity of the “Parent report and Adult versions of Technology Use Questionnaire” and describe the technology use among young children and their parents.	Cross-sectional. Questionnaire-based.	96; parents; 89% women; mean age 36.9 (SD 5.0) years.	Parents were recruited from not-for-profit childcare organizations and childcare centers from 2 different cities.
Hutton et al (2018) [71], United States, HIC	To investigate attitudes and behaviors regarding shared reading and infant television viewing during the perinatal period among low socioeconomic status mothers.	Longitudinal. Questionnaire-based.	282; parents; 100% women; mean age 22.9 (SD 4.6) years.	All mothers were enrolled in a randomized controlled trial of safe sleep education.
Ihmeideh and Alkhawaldeh (2017) [72], Jordan, UMIC	To investigate the perceptions of preschool teachers and parents on how technology and digital media contribute toward developing child culture in Jordanian early years education.	Cross-sectional. Mixed method (questionnaire and interview).	480; teachers (n=170) and parents (n=310); gender NR; age NR.	Preschool teachers and parents of children were recruited randomly from a list of all private kindergartens.
Ilgar and Karakurt (2018) [73], Turkey, UMIC	To investigate the general attitudes of mothers of preschoolers toward the impact of computer games on children’s development and behavior.	Cross-sectional. Questionnaire-based.	749; parents; 100% women; age NR.	NR.
Istenič et al (2023) [74], Slovenia, HIC	To investigate Slovene parents’ opinions and perceptions of play and how they structure their child’s play by offering them a set of toys.	Cross-sectional. Questionnaire-based.	306; parents; 90% women; age NR.	NR.
Istenič et al (2023) [75], Slovenia, HIC	To investigate how parents (1) perceive digital technology and its role in a child’s development and learning, (2) perceive characteristics of a child’s play with traditional and digital toys, and (3) construct the home environment and play regarding a share of digital play versus traditional.	Cross-sectional. Questionnaire-based.	306; parents; 90% women; age NR.	NR.
Jain et al (2023) [76], India, LMIC	To determine the prevalence and predictors of excessive screen viewing time in children and its effect on physical or outdoor activity, sleep, and the prevalence of eye symptoms and headache in children in India.	Cross-sectional. Questionnaire-based.	Parents of 600 children answered a questionnaire when the children were younger than 10 years but unclear how many parents answered the questionnaire; parents; gender NR; age NR.	Households with children aged 3‐15 years were identified during house-to-house visits in 36 urban wards and 36 villages using a 3-stage cluster sampling method.
Jin (2013) [77], Korea, HIC	To investigate (1) the relationship between 5 types of parental internet content filtering software (ICFS) adopters and the parents’ adoption of ICFS, (2) how adoption type affects parents’ perception of and satisfaction with ICFS, and (3) parental attitudes toward their children’s internet use.	Cross-sectional. Questionnaire-based.	784; parents; 51% women; 69% aged 40‐49 years.	Parents were randomly selected through consumer panel data on ICFS users. Active panel members were contacted by email and phone and asked to participate.
Joginder Singh et al (2021) [78], Malaysia, UMIC	To investigate (1) the amount of screen time reported by parents of 3‐ to 5-year-old children, (2) how parental perception of screen time affects their children’s language skills, and (3) the association between reported screen time and parental perceptions of how screen time affects their children’s language skills.	Cross-sectional. Questionnaire-based.	340; parents; 85% women; 70% aged 31‐40 years.	Parents were recruited online through WhatsApp, Telegram, and Facebook groups.
Konok et al (2020) [79], Hungary, HIC	To investigate whether parents’ digital parenting style, general attitude, and beliefs regarding early mobile touch screen device use and role-modeling as indicated by their mobile attachment and use are associated with child digital activity.	Cross-sectional. Questionnaire-based.	1270; parents; 83% women; mean age 35.41 (SD 5.06) years.	Parents were recruited online through Facebook and the website of a Hungarian online magazine.
Kostyrka-Allchorne et al (2017) [80], United Kingdom, HIC	To investigate (1) media preferences and use among young children and (2) parental supervision methods and beliefs about media.	Cross-sectional. Questionnaire-based.	90; parents; 91% women; age NR.	Parents were recruited from 2 primary schools and 4 preschools.
Lee et al (2022) [81], Korea, HIC	To investigate the association between young children’s media use and parents’ media use, attitudes toward media, and parenting style.	Cross-sectional. Questionnaire-based.	1020; parents; 50% women; age NR.	Parents were recruited from a national panel pool by a Korean survey company.
Lepicnik-Vodopivec and Samec (2013) [82] Slovenia, HIC	To investigate (1) how many types of information-communication technology (ICTs) the child’s family owns, (2) the nature of the child’s access at home, (3) how the child uses ICTs at home, (4) how often the child uses ICTs at home, (5) the influences on the child’s use of ICTs at home, (6) the influence of the child’s ICT use on their development, (7) the child’s attitude toward ICTs at home, and (8) the parents’ awareness about ICT use.	Cross-sectional. Questionnaire-based.	130; parents; 84% women; age NR.	NR.
Li and Chen (2015) [83], China, UMIC	To investigate how preschool children use tablets and the extent to which their parents’ media behavior influences the children’s tablet use.	Cross-sectional. Mixed method (questionnaire and interview).	161; parents; gender NR; age NR.	Parents were recruited from 3 kindergartens.
Liibaan et al (2023) [84], Scotland, HIC	To investigate parents’ opinions of screen time, including impact on children’s sleep, behavior, development, and underlying factors surrounding use and awareness of national guidelines.	Cross-sectional. Mixed method (questionnaire and interview).	9; parents; gender NR; age NR.	Parents were recruited via social media posts.
Little (2019) [85], United Kingdom, HIC	To investigate heritage language families’ attitudes to using game-based digital technology for heritage language and literacy development, and how these attitudes are associated with children’s self-awareness as heritage language speakers.	Cross-sectional. Mixed method (questionnaire and interview).	212; parents; gender NR; age NR.	Parents were recruited via social media posts, posted in 6 online parenting groups linked to heritage language or bilingualism.
Luo et al (2023) [86], Taiwan, HIC	To investigate parents’ attitudes toward their young children’s use of information communication technology, use patterns, and the relationship with socioeconomic status.	Cross-sectional. Questionnaire-based.	629; parents; 78% women; 40% were aged between 36 and 40 years.	Parents were recruited via kindergartens and elementary schools using a convenience sampling technique.
Mansor et al (2021) [87], Malaysia, UMIC	To investigate parental barriers toward reducing a child’s screen time and its predictors from sociodemographic, parental, child-related, and environmental factors among parents of children younger than 5 years of age who attended a child health clinic in Malaysia.	Cross-sectional. Questionnaire-based.	789; parents; 84% women, mean age 31.6 (SD 4.8) years.	Parents were recruited from 9 health clinics based on an attendance record from each clinic.
Matziou et al (2021) [88], Greece, HIC	To investigate (1) preschool children’s sleeping and television viewing habits, (2) parents’ perceptions about television viewing, and (3) potential correlation between the 2.	Cross-sectional. Questionnaire-based.	100; parents; gender NR; mean age of fathers 37.4 (SD 4.4) years, mean age of mothers 32.9 (SD 4.0) years.	Parents were recruited from randomly selected kindergartens.
Mikelić Preradović et al (2016) [89], Croatia, HIC	To investigate (1) perceptions of parents in Croatia toward computer use in general and their children’s computer use and (2) parents’ concerns and opinions about digital technology education in kindergarten.	Cross-sectional. Questionnaire-based.	152; parents; 72% women; 64.4% were aged 30‐39 years.	Parents were recruited from a public early childhood educational institution.
Milford et al (2022) [90], Australia, HIC	To investigate how parents access information related to mobile media and their perspectives about the impact of mobile media on their child’s behavior and executive functioning.	Cross-sectional. Questionnaire-based.	213; parents and other caregivers; 95% women; 45% were aged 31‐40 years.	Parents or caregivers were recruited by posting advertisements on social media platforms including Facebook and Twitter and promoting within the community.
Nabi and Krcmar (2016) [91], United States, HIC	To investigate how perceived child characteristics influence parents’ motives for allowing their children to use screen media.	Cross-sectional. Questionnaire-based.	151; parents; 82% women; age NR.	Parents were recruited from daycare centers, organized parent groups, and online snowball sampling, in which a link to the online version of the survey was sent to several parents, who then forwarded the link to others. The survey link was also posted on social networking sites.
Natsiopoulou et al (2013) [92], Greece, HIC	To investigate Greek parents’ views on integrating computers into the preschool classroom and how they relate to family socioeconomic background.	Cross-sectional. Questionnaire-based.	280; parents; gender NR; age NR.	Parents were recruited from private and municipal preschool centers.
Nikken (2019) [93], Netherlands, HIC	To investigate whether parents see media devices as useful tools in child-rearing, and how parent-family characteristics and parental perceptions on parenting, media effects, and child development predict the acceptance of instructional media use.	Cross-sectional. Questionnaire-based.	516; parents; 68% women; age NR.	Parents were recruited online by a professional marketing research bureau.
Nikken and Schols (2015) [94], Netherlands, HIC	To investigate if children’s media skills and activities, parents’ attitudes about media for children, and other child and parent characteristics predicted parental mediation practices.	Cross-sectional. Questionnaire-based.	896; parents; 47% women; mean age 37.3 (SD 6.1) years.	Parents were recruited from a large online panel.
Njoroge et al (2013) [95], United States, HIC	To investigate the associations between race or ethnicity, family socioeconomic status, and child television or DVD viewing, while assessing differences in ethnically diverse parents’ beliefs and attitudes regarding the impact of television or DVD viewing on early childhood development.	Cross-sectional. Questionnaire-based.	596; parents; gender NR; age NR.	Parents were recruited from 2 metropolitan Seattle pediatric clinics and a pediatric practice network. Letters describing the study were sent to families with 3‐ to 5-year-old children without regard to whether the child had been seen in their pediatric clinic recently.
Nwankwo et al (2019) [96], United Kingdom, HIC	To investigate the drivers of screen-related sedentary behavior within the home context and parents’ attitudes in supporting children’s associated behavior.	Cross-sectional. Mixed method (questionnaire and interview).	140; parents; 58% mothers; 49% were aged 36‐45 years.	Parents were recruited by sending a survey link through emails or social media groups (Facebook, WhatsApp, and Twitter).
O’Connor and Fotakopoulou (2016) [97], United Kingdom, HIC	To investigate (1) what 0‐ to 3-year-old children are doing with touch-screen devices in UK families, (2) what parents perceive as the advantages and drawbacks of their 0‐ to 3-year-old children using new technology, and (3) the extent to which the justification of their practices appears to be aligned with dominant discourses around young children and technology.	Cross-sectional. Questionnaire-based.	226; parents and grandparents; 90% women; 67% were aged 31‐40 years.	Parents were recruited by sending the online questionnaire to personal, parenting, and professional networks.
Ophir et al (2023) [98], Israel, HIC	To investigate the extent to which children’s screen time increased during the COVID-19 lockdown and to evaluate mothers’ consequent negative emotional reactions (study 1); and to investigate what could be contributing to the increases in screen time (study 2).	Cross-sectional with 2 independent cohorts. Questionnaire-based.	Study 1: 299; mothers; mean age was 41 (SD 5) years. Study 2: 283; mothers; mean age was 37 (SD 6) years.	Mothers were recruited from a survey company in Israel (study 1) and an Israeli college (study 2).
Van Petegem et al (2019) [99], Belgium, HIC	To investigate associations between parents’ degree and style of restrictive mediation of children’s digital gaming and parents’ attitude about digital gaming, perceptions of children’s defiance and problematic gaming, and their interest in social play.	Cross-sectional. Questionnaire-based.	762; parents; 83% women; mean age 35.27 (SD 5.65) years.	A large-scale survey was distributed through the mailing list of a child-oriented entertainment company. Parents with a child aged between 3 and 9 years were invited to participate.
Raj et al (2022) [100], Malaysia, UMIC	To investigate the prevalence and determinants of excessive screen time among children younger than 5 years in Selangor, Malaysia.	Cross-sectional. Questionnaire-based.	489; parents; 48% women; mean age 32.2 (SD 0.2) years.	Parent-child dyads were recruited from 9 child health clinics.
Raj et al (2023) [101], Malaysia, UMIC	To develop, implement, and evaluate the effectiveness of a digital parental health education intervention to reduce excessive screen time among preschoolers from low socioeconomic families in Malaysia.	Randomized controlled trial. Questionnaire-based.	360; mothers; mean ages were 34 (SD 4.3) years in the intervention group and 33 (SD 5) years in the control group.	Mothers were recruited from preschools via electronic pamphlets containing information about the study. Interested mothers self-registered for the study using a Google Form link.
Rajalakshmi et al (2023) [102], India, LMIC	To investigate the burden of digital screen exposure and parental perceptions of its effects in children.	Cross-sectional. Questionnaire-based.	140; caregivers; 84% women; age NR.	Adults were recruited from tertiary health care centers using a random sampling technique.
Rosanda et al (2022) [103], Slovenia, HIC	To investigate (1) the use of digital technology by Slovenian infants and toddlers younger than 2 years of age, (2) what types of digital devices and apps young children use in their play, and (3) the parents’ perspectives about their children’s use of digital devices.	Cross-sectional. Questionnaire-based.	26; 25 parents and 1 aunt; gender NR; mean age 33.5 (SD 4.3) years.	NR.
Sada Garibay and Lapierre (2024) [104], Mexico, UMIC	To investigate what drives parents to use technical restriction tools offered by Subscription Video on Demand services.	Cross-sectional. Questionnaire-based.	1022; parents; 65%women; 44% were aged between 41 and 50 years.	Parents were recruited via Facebook, Instagram, student WhatsApp accounts, and other social media.
Peštaj et al (2024) [105], Slovenia, HIC	To investigate parental views on the characteristics of a quality children’s program and its positive and negative effects on children’s development and learning.	Cross-sectional. Questionnaire-based.	239; parents; 88% women; mean age was 34.9 (SD 5.24) years.	Parents were recruited via Facebook and emails by a counselor in the preschool where their children were enrolled.
Solomon-Moore et al (2017) [106], United Kingdom, HIC	To investigate whether parents’ attitudes toward their young child’s screen viewing behavior are associated with parents’ sedentary time and screen viewing behaviors.	Cross-sectional. Questionnaire-based.	809; parents; 74% women; age NR.	Child-parent dyads were recruited from 57 primary schools in the greater Bristol area.
Stuckelman et al (2023) [107], United States, HIC	To investigate the inclusion of parent-directed suggestions or “nudges” in a touchscreen game app to promote positive shared family interactions during digital coplay.	Randomized controlled trial. Questionnaire-based.	77; parents; 92% women; control group mean age was 36.15 (SD 7.07) years, experimental group mean age was 35.45 (SD 6.74) years.	Parent-child dyads were recruited from social media posts, state birth records, and a university database of families interested in research participation.
Suresh and Tiwari (2023) [108], India, LMIC	To investigate the parental knowledge, attitudes, and concerns toward media technology and screen time use by preschool children with autism spectrum disorder (ASD) and typically developing (TD) children in the Indian context.	Cross-sectional. Questionnaire-based.	182; parents (92 parents of TD children, 90 parents of children with ASD); 83% women in TD group, 86% women in ASD group; parents in both groups were aged between 20 and 49 years.	Parents in the ASD group were recruited from department clinics, regular schools, special schools, parent groups for children with ASD, and private speech therapy clinics. Parents in the TD group were recruited through regular primary schools, preschools, and day care centers.
Tanusha et al (2023) [109], Malaysia, UMIC	To investigate parents’ perception of digital device use among their preschool children.	Cross-sectional. Questionnaire-based.	139; parents; gender NR; age NR.	Parents were recruited from their children’s kindergartens. In total, 10 government kindergartens were randomly selected, and 15 children were selected randomly by computer from each school using the student registry.
Tay et al (2021) [110], Singapore, HIC	To investigate parents’ attitudes and concerns toward their preschoolers’ use of technology and digital media in Singapore.	Cross-sectional. Questionnaire-based.	3413; parents; gender NR; age NR.	Parents were recruited via letters, with a QR code to scan and complete the survey, sent out by the research team.
Vaala and Hornik (2014) [111], United States, HIC	To investigate (1) the relationships between American infants’ and toddlers’ television or video viewing time and their mothers’ demographics, structural life circumstances, and cognitions and (2) the extent to which mothers’ cognitions mediate relationships between the structural circumstances of mothers’ lives and their infants’ or toddlers’ estimated television or video viewing.	Cross-sectional. Questionnaire-based.	698; mothers; 100% women; mean age 28.5 (SD 6.6) years.	Mothers were recruited through a national panel of around 1 million members. The panel sent recruitment emails to panel members who fit the criteria for study participation.
Vaiopoulou et al (2021) [112], Greece, HIC	To investigate (1) the psychometric characteristics of the Perceptions about Educational Apps Use-parents (PEAU-p) instrument and (2) explore parents’ perceptions and attitudes toward using educational apps by implementing the PEAU-p instrument.	Cross-sectional. Questionnaire validation study.	435; parents; 96% women; mean age 36.88 (SD 5.21) years.	Parents were recruited using an electronic sampling process via social media groups.
Vittrup et al (2016) [113], United States, HIC	To investigate parental media attitudes and perceptions of their children’s knowledge and engagement with various media technologies and to explore their actual knowledge and experience with these tools.	Cross-sectional. Mixed method (questionnaire and interview).	101; parents; gender NR; mean age 36 (range 25‐53) years.	Parents were recruited from childcare centers, homeschool networks, and institutions of higher education via flyers with study information and packets of surveys.
Vincent and Blot (2021) [115], France, HIC	To investigate (1) the proportion of parents who report having discussed the subject of their child’s exposure to screens during a consultation with a health professional, (2) preschool children’s exposure to television and mobile media devices, and (3) parents’ views on the benefits and risks of exposing their children younger than 3 years of age.	Cross-sectional. Questionnaire-based.	451; parents; gender NR; median age was 34 (IQR 31‐38) years.	Parents were recruited from 8 nurseries and a hospital’s pediatric emergency room.
Wang et al (2024) [114], China, UMIC	To investigate Chinese intergenerational caregiving during the COVID-19 pandemic and different types of caregivers’ beliefs, practices, and communication with children through various digital tools in the home environment.	Cross-sectional. Mixed method (questionnaire and semistructured interview).	264; parents and grandparents (185 mothers, 8 fathers, and 71 grandparents); 70% women; age NR.	Adults were recruited from preschools.

a Country income level was classified according to the World Bank income categories.

b Covers both gender and sex, depending on how this was reported in the study.

c UMIC: upper-middle-income country.

d NR: not reported.

e HIC: high-income country.

f LMIC: lower-middle-income country.

Among the 85 included studies, most were cross-sectional (74/85, 87%), 70 were based on questionnaire responses only, and 15 used a mixed methods approach of both questionnaires and interviews. The studies were conducted in a wide range of countries, but primarily in the United States (11/85, 13%), the United Kingdom (5/85, 6%), and Australia (5/85, 6%). The average number of study participants was 495 (SD 562; range 10‐3413), of which, 82 studies only consisted of parents. In 3 studies, grandparents were also included [47,57,97]. No studies included health professionals. Accordingly, only parental perspectives are reported on in this paper. Among the 59 studies that reported participants’ gender, most were women (mean 80%, SD 14%; range 47%‐100%). Participants’ age was reported in 46 studies and mostly ranged between 30 and 40 years.

### Characteristics and Psychometric Properties of Questionnaires Used in Included Studies

Multimedia Appendix 3 [31-146] provides the characteristics of questionnaires used among the 85 included studies. In total, 19 studies reported the title of the questionnaire, and 76 reported the item type. Among the studies describing questionnaire item type, 52 reported using questionnaires with closed-ended items, and 24 used questionnaires with a combination of closed- and open-ended items. A total of 63 studies provided information on administration format, of which most (n=42) used an online questionnaire. Information on how the questionnaire was developed was provided by 74 studies, most commonly (n=45) reported as being developed specifically for the study.

In total, 30 studies reported the total number of items in the questionnaire (mean 34; range 6‐86), and 67 studies reported the number of items specifically related to parents’ perspective of children’s use of digital technologies (mean 11; range 1‐33). Details on item scoring was reported in 61 studies, with a Likert-scale to measure level of agreement most commonly used (n=49).

Psychometric properties of questionnaires were reported by 52 studies. Among these, 14 studies conducted pilot tests to ensure face validity [33,34,47,52,55,71-73,76-78,93,100,109]. In total, 12 studies evaluated content validity by consulting experts within the field [39,44,52,53,64,65,72,73,76,86,108,110], of which, 3 studies reported content validity indexes, which ranged between 0.83 and 1.00 [44,76,108].

A total of 10 studies reported measures of construct validity based on results from a type of factor analysis [39,53,57,64,86,91,93,98,99,112]. Confirmatory factor analysis (CFA) was used in 4 studies [53,57,99,112], though the reported metrics varied. Chen and Tu [53] reported a root-mean-square error of approximation (RMSEA) of 0.06, a standardized root-mean-square residual (SRMR) of 0.05, a composite reliability of 0.88, and average variance extracted of 0.66. Dong et al [57] also performed a CFA and found that the model explained 43% of total variance, with eigenvalues ranging from 13.18 to 2.92, but no additional performance metrics were reported. Van Petegem et al [99] reported an RMSEA of 0.06 and SRMR of 0.02, while Vaiopoulou et al [112] found an RMSEA of 0.027 and SRMR of 0.07. Exploratory factor analysis was applied in 5 studies [39,64,86,93,112]. Balaban Dağal and Bayındır [39] used principal component analysis, reporting factor loadings of 0.54‐0.87. González-Sanmamed et al [64] found loadings between 0.52 and 0.90, while Luo et al [86] identified a 2D structure with an internal reliability of 0.95 (specific metric not reported). Nikken [93] identified a 5D structure, with item loadings >0.45, and Vaiopoulou et al [112] used principal component analysis alongside CFA, reporting factor loadings of 0.54‐0.89. In total, 2 studies conducted a factor analysis, but did not specify which type [91,98]. Nabi and Krcmar [91] identified a 2D structure, while Ophir et al [98] reported a 2D structure explaining 69%‐70% of variance, with factor loadings ranging from 0.69 to 0.81.

Cronbach α was predominantly reported (n=26 studies) as a measure of internal consistency reliability, ranging from 0.24 to 0.95 (mean 0.80), and one study used McDonald ω as a measure of internal consistency [46], which ranged between 0.62 and 0.69. A total of 7 studies reported a measure related to assessing reliability, but did not specify which metric this was based on [77,81,86,91,100,101,109]. One study reported a test-retest reliability based on intraclass correlation (ranging between 0.64 and 0.98) [87].

Taken together, these findings show that internal consistency reliability was the most frequently reported psychometric property (n=35), whereas fewer studies reported evidence of validity, including face validity (n=14), content validity (n=12), and construct validity [10]. Few studies (n=15) reported more than a single aspect of validity and/or reliability.

### Focus of Questionnaires Used in Included Studies

A total of 67 studies reported either all or some examples of the questionnaire items used to measure parents’ perspectives on children’s technology use (Table S1 in Multimedia Appendix 4 [31-115]). When considering which type of digital technology each questionnaire covered (ie, results of the level 1 coding), most questionnaires focused on digital media or digital devices (n=22), where technology was broadly referred to without a particular hardware, software, or content type. In total, 12 studies focused on screen time, which either covered children’s exposure to any screens or to specific device types (eg, television and smartphones) [37,40,44,49,50,67,76,78,87,98,106]. Among specific device types, 9 studies focused on children’s exposure to mobile touch screen devices (eg, smartphones and tablets) [32-34,55,56,70,79,97,113], and 8 studies focused on television viewing [38,42,43,62,71,94,95,111]. A total of 7 studies did not specify a particular device (eg, referring to general internet use) [35,48,53,77,102,104,105]. Gaming and computer use were referred to in 4 [36,39,75,99] and 3 [73,89,92] studies, respectively. Finally, 2 studies focused on applications [60,112].

Table 2 provides an overview of the content focus of the questionnaire items according to the 4 overall dimensions of the child-technology interaction model. When considering the overall dimensions covered, most of the 437 reported items were related to the child dimension (n=187). Within this dimension, the most common content focus of the items was parents’ perspectives on the potential impact of technology on children’s physical health (n=48), including sleep issues and lack of physical activity, followed by the potential influence of technology on children’s emotions and behaviors (n=47) and cognition (n=12). A total of 56 items could be considered as having a content focus under the other people dimension, of which, most items aimed to measure parental regulation of children’s technology use (n=32). A total of 176 items were related to the task dimension, of which, most focused on parents’ perspectives on the potential impact of technology use on how children learn (n=82) and how technology can be used for social aspects (n=40). Only 14 items could be considered as related to the technology dimension, of which, safety was the most common theme (n=16).

**Table 2.** Classification of items according to the 4 dimensions of the child-technology interaction model, including codes used to classify each item.

Code^a^	Definition of code	Values (N=437), n (%)
“Child” dimension (n=187, 43%^a^)		
Addiction	Item relates to child’s addiction to technology.	12 (3)
Age of child	Item relates to the age of the child. Can either be mentioning a specific age (eg, 5 years) or range (eg, young children).	17 (4)
Attention	Item relates to technology’s impact on children’s attention.	10 (2)
Cognition	Item relates to technology’s impact on children’s cognition. Includes aesthetic (eg, order and beauty).	21 (5)
Creativity	Item relates to technology’s impact on children’s creativity.	12 (3)
Digital literacy	Item relates to children having the skills to use the technology.	8 (2)
Emotions and behaviors	Item relates to technology’s impacts on children emotionally and/or their behavior (eg, makes child aggressive).	47 (9)
Physical health (including sleep issues)	Item relates to technology’s impact on children’s physical health.	48 (11)
Religion and culture	Item relates to technology’s impact on children’s religion and/or culture (eg, by helping children learn about religion or may influence cultural values).	3 (1)
Well-being	Item relates to technology’s impact on children’s overall well-being (eg, by influencing mental health or overall quality of life).	9 (2)
“Other people” dimension (n=56, 13%^a^)		
Family interaction	Item relates to how children’s technology use can promote or hinder family time or family interaction.	24 (5)
Parental regulation of technology	Item relates to regulations of technology use.	32 (7)
“Tasks” dimension (n=176, 40%^a^)		
Displacement of activities	Item relates to time spent on digital technology instead of other activities such as normal or traditional play.	11 (3)
Entertainment and relaxation	Item relates to technology as being entertaining or a way of relaxation for children.	22 (5)
Keeping children occupied	Item relates to technology as being a way to “keep children occupied.”	16 (4)
Learning	Item relates to children using digital technology for learning.	82 (19)
Preparing for adult life	Item relates to how technology can prepare children for adult life.	5 (1)
Social	Item relates to how technology can be used for or influences social aspects (eg, by influencing social skills or level and quality of social interactions).	40 (9)
“Technology” dimension (n=14, 4%^a^)		
Internet and online	Item relates to children using the internet or being online.	2 (<1)
Safety	Item relates to whether adults feel safe about their children using technology or to the age-appropriateness of content.	16 (4)

a Each item was only coded as belonging to one of the listed codes.****

### Perspectives From Included Studies’ Participants

A total of 75 studies reported the study participants’ perspectives on children’s use of technology. Table S1 in Multimedia Appendix 5 [31-115] provides a full summary of study findings related to perspectives toward children’s technology use based on questionnaire responses. Multimedia Appendix 6 shows the distribution of reported perspectives within each of the 4 dimensions of the child-technology interaction model (ie, child, other people, tasks, and technology) according to the classification codes represented in Table 2. Please note that perspectives were coded based on reported findings in Table S1 in Multimedia Appendix 5; thus, each study could contribute to multiple themes.

Across the 4 dimensions of the child-technology interaction model, parents expressed diverse perspectives on children’s use of technology.

When considering the child dimension, 23 studies reported that parents were concerned about potential negative impacts of technology on their children’s physical health. Commonly raised concerns were related to lack of physical activity, sleep issues, and poor eyesight. In total, 4 studies reported that parents believed that technology could have beneficial impacts on their children’s physical health, such as improvement in movement skills [45,72,82,103]. A total of 13 studies reported that parents were concerned about technology leading to addiction [31,37,47,52,59,64,65,73,83,86,97,105,109]. Parents’ perspectives on the impact of technology on children’s emotions and behaviors were addressed in 11 studies [40,68,72-74,82,93,102,105,113,115]. While most of these studies (n=9) reported that parents had concerns about technologies leading to behavioral issues [40,68,73,74,93,102,105,113,115], some also reported that parents believe technology could have positive impacts on children’s emotions and behaviors, for example, in supporting emotional regulation [72,82,93,105].

When considering the task dimension of the child-technology interaction model, a total of 41 studies reported perspectives related to the use of and/or potential influence of technology on children’s learning. Among these studies, all participants reported positive perspectives, particularly in relation to the role of technology for enhancing children’s academic performance. Mixed perspectives were found for the role of technology on children’s social development. In total, 9 studies reported that the participants had concerns about technology negatively impacting children’s social skills and development [47,56,70,73,81,93,105,108], while 4 studies reported that the participants believed technology had a positive impact, particularly in relation to social interaction [68,72,82,105]. A total of 9 studies reported that parents acknowledged technology as a positive source of entertainment and relaxation for children [32,38,47,50,56,62,92,105,110].

For the other people dimension, parents were concerned about technology interfering with family time [37,68,73,89]. Finally, for the technology dimension, a commonly held concern among the study participants was related to children’s safety when using technology, particularly in relation to children being exposed to inappropriate content [47,51,52,59,60,64,70,80].

## Discussion

### Principal Findings

This systematic review aimed to investigate the psychometric properties of questionnaires that have been used to measure perspectives of parents and health professionals on young children’s technology use and to synthesize these perspectives. Overall, the majority of the included studies were of questionable methodological quality (n=62, 73%), particularly in terms of describing the development and validation of the questionnaires used for measuring perspectives. Of the 85 included studies, 52 studies reported psychometric properties of the questionnaire used, but the psychometric evaluation was often limited and incomplete, often focusing on a single metric for validity. When assessing the questionnaires themselves, most items focused on asking participants about how technology could aid children’s learning or how it could influence their physical health, emotions and behaviors, and social skills. As none of the studies recruited health professionals, we could only provide a synthesis of parental perspectives on young children’s technology use. This synthesis indicated that parents generally had positive perspectives on the use of technology for academic and educational purposes, but concerns were expressed regarding the potential negative influences of technology on children’s health and well-being.

High-quality methods for capturing the perspectives of parents and health professionals on children’s technology use are essential for obtaining accurate, unbiased, and reliable data. The development of a valid and reliable questionnaire is an extensive process, in which several steps are taken to ensure face, content, and construct validity as well as internal reliability [147,148]. Overall, while many studies included in this review reported some acceptable validity and reliability metrics, gaps remained in the consistent use of metrics, with only 15 studies evaluating more than 1 measure of validity and/or reliability [39,52,53,57,64,72,73,76,77,86,93,99,100,108,109]. Moreover, only 3 studies specifically aimed to develop and validate a questionnaire for measuring parents’ perspectives on children’s technology use [33,39,112]. Among these, the study by Balaban Dağal and Bayındır [39] conducted the most detailed validity and reliability analysis, assessing content, construct, and internal validity as well as test-retest reliability. In contrast, the study by Al-Balushi and Al-Shihi [33] only evaluated face and content validity, and the study by Vaiopoulou et al [112] only evaluated construct and internal validity. Overall, these findings suggest that there is a need for further development of high-quality questionnaires within this research field.

When assessing the focus of questionnaire items used to measure perspectives, there was a lack of clarity and heterogeneity in the definition of technology adopted by studies. Most studies used the term “digital media” or “digital devices,” which covered a range of hardware, software, and content. The lack of specificity could have influenced the responses from the study participants. For example, for the same item, some respondents may have thought of digital media as being social media, while others may have considered it to be television programs or learning apps. This could lead to inconsistent or even contradictory results and limit the accuracy and comparability of study findings. When considering the questionnaire items’ focus, most items aimed to measure perspectives related to the potential impact of technology on how children learn, their physical health, emotions and behaviors, as well as social skills and interactions. It is worth noticing that the phrasing of the items themselves differed substantially both within and between studies, with some being negatively phrased (eg, “[computers] have a negative impact on children’s socialisation” used in Ilgar and Karakurt [73]) and others being positively phrased (eg, “[digital media] help children communicate and interact with others” used in Ihmeideh and Alkhawaldeh [72]). This choice of item phrasing may stem from an implicit position of technological determinism, in which technology is believed to be socially causative [149]. It may be that the assumptions underlying the item development (eg, that technology use is harmful) and phrasing of the items directly influenced how participants interpreted the question and thereby how they chose to reply [150]. Specifically, negatively phrased items might have primed the respondents to focus on potential harms of technology use, whereas positively phrased items might have encouraged considerations of potential benefits. The framing of questions may also be influenced by the discipline context of the research team. As a result, the overall conclusions drawn about parental perspectives on children’s technology use may be biased.

Despite the lack of construct clarity and large variability in phrasing of items, some general trends were found when synthesizing the parental perspectives reported among the included studies. In general, positive perspectives were reported in relation to how technologies can be used for enhancing children’s learning, particularly in terms of academic skills and digital literacy. In contrast, concerns were often raised about technology’s impact on children’s physical health and emotions and behaviors. Mixed perspectives were found in relation to children’s social skills. Such positive and negative perspectives on young children’s use of technology have also been reported for early childhood educators [151]. Our results also concur with the findings of previous qualitative studies of parents [18-20]. The reviews by Choy et al [19], Chong et al [18], and Visier-Alfonso et al [20] reported parental concerns about technology negatively impacting their children’s physical (eg, eyesight and sleep patterns), attention issues, and social development. However, Choy et al [19] also reported that parents believed that digital devices could positively contribute to children learning new skills. Taken together, the findings from previous qualitative studies and our results suggest that parents have mixed perspectives on the role of technology in their children’s lives. These perceptions are likely influenced by a range of factors, including sociodemographic, cultural influences, parental age, age of their children, and parents’ own experiences with technology [16]. Moreover, parental mixed perspectives may also be related to a lack of clear, evidence-based guidance on healthy technology use [17], although existing evidence does support parents’ perspectives that technology use has potential risks and benefits [5].

### Practical Implications

Based on our evaluation of questionnaires used for capturing the perspectives of parents on children’s technology use, it is apparent that more work is required for developing high-quality questionnaires within this research field. The lack of validated questionnaires to capture the perspectives of health professionals also highlights a need. We encourage future research to prioritize the development of accurate, unbiased, and reliable questionnaires and propose that several steps are followed to support this. First, we would advise researchers to clearly define the research question, target population, their literacy level, and constructs aimed to be measured. Some of the key considerations include the type of technology, the age of children, the purpose of technology use, and the setting in which technologies are used, as these are likely to influence adults’ perspectives. For example, gaming consoles could be more appropriate to ask parents of older children about who are commonly more exposed to this technology compared to younger children [3]. We would also encourage researchers to carefully consider which aspects of technology to measure, including not only the amount of time using technology (ie, quantity) but also the content and context in which it is used (ie, quality). Second, when developing the items and item response categories, we suggest using clear and concise language with balanced statements regarding potential benefits and risks associated with children’s technology use to ensure that the respondents are not led toward a particular answer. Finally, we highly recommend that researchers thoroughly evaluate the validity and reliability of their questionnaire by using well-established approaches for measuring face, content, construct, and internal validity as well as assessing stability over time. Until further validated questionnaires are developed, we recommend the use of existing questionnaires with substantive validity evidence, such as the Balaban Dağal and Bayındır [39] questionnaire, if it matches the study’s research question.

Our synthesis of perspectives revealed that parents have mixed views on the role of technology in their children’s lives. While the potential benefits of technology, particularly for enhancing academic skills and social interactions, were acknowledged, concerns were often expressed about potential negative impacts on children’s physical and mental health and overall development. These findings suggest that parents are cognizant of the complex role of technology in young children’s lives, and this aligns with prior reports that parents are struggling to navigate conflicting narratives surrounding children’s technology use [17]. These findings highlight several priorities for future research. Specifically, parents may be exposed to a broad range and, at times, controversial guidance from educators, health professionals, and media sources, making it challenging to make decisions regarding healthy technology use. We encourage future research to investigate the basis for parent perspectives, including what influences their perspectives. Moreover, prior research has reported sources parents use to gain information about health issues [16], but whether parents have the ability to independently develop a clear understanding of the evidence regarding the likely impacts of technology use on child outcomes is not known. Thus, this presents an important area for further investigation. Future research should also investigate the impact of social desirability on parental perspectives. Further, more work is required to understand whether perspectives are evidence-based or reflect parental views on ideal childhoods and other values. Addressing these research gaps would enable educators and health professionals to better support parents through the challenges of raising children in a digital world by providing a better understanding of how parents’ perspectives are developed and how these relate to their decision-making regarding their children’s use of technology.

### Strengths and Limitations

Strengths of this systematic review included the developed search strategy applied in a wide range of databases as well as the cross-checking of study screening, quality assessment, and data extraction independently by 2 authors (CLR and IPHA). The development of a comprehensive, theory-based, 2-level coding framework for classifying the focus of the questionnaires and the items used was another strength. This framework, based on the child-technology interaction model [14], enabled a structured and systematic approach for assessing the diverse ways in which studies measured parental perspectives. Although we acknowledge that some codes could be classified under multiple dimensions of the child-technology interaction model, the application of this structured framework enhances the reproducibility of our findings and provides an early foundation for future research seeking to improve the measurement of perspectives on children’s technology use.

Only reports published in English were considered for inclusion, and potentially relevant studies might have been missed due to unclear titles or abstracts and artificial intelligence–supported screening. Moreover, we aimed to report how perspectives on children’s technology use may depend on the population group (eg, parents and health professionals) and the age of the child. However, these assessments were not feasible, as the only participants in the included studies were parents, and the age of the children was rarely specified.

### Conclusions

This systematic review found that parents’ perspectives on children’s technology use have been assessed using questionnaires in several studies. However, the reported psychometric evaluation of the questionnaires was limited and incomplete, with most studies focusing on a single metric. Further, varied definitions of technologies make it difficult to establish to what extent the existing literature is examining variations in technologies relative to parents’ perspectives. Study participants consisted of parents only, highlighting a clear evidence gap in understanding health professionals’ perspectives on children’s technology use. The reported parental perspectives were mixed. While positive views were found, considering the use of technology for enhancing children’s learning and skill development, concerns were commonly raised about potential negative impacts on children’s physical health and emotions and behaviors. As parents’ perspectives may influence their children’s use of technology and potentially associated development, there is a need for better-quality tools to measure adults’ perspectives, understand how perspectives are developed, and how they influence practice. We encourage such work to focus on improving questionnaire design based on a clear theoretical understanding of technologies to prevent confusion in defining terms and item phrasing.

## Supplementary material

10.2196/84712 Multimedia Appendix 1 Search strategy used for the following databases: PsycInfo, Web of Science (Core Collection), CINAHL, SPORTDiscus, Embase, MEDLINE, Scopus, and ProQuest.

10.2196/84712 Multimedia Appendix 2 Results of the quality assessment of included studies.

10.2196/84712 Multimedia Appendix 3 Characteristics and psychometric properties of questionnaire used.

10.2196/84712 Multimedia Appendix 4 Reported questionnaire items used for measuring parents’ perspectives on children’s technology use.

10.2196/84712 Multimedia Appendix 5 Full summaries of study findings related to perspectives toward children’s technology use based on questionnaire responses.

10.2196/84712 Multimedia Appendix 6 Distribution of reported perspectives within each of the 4 dimensions of the child-technology interaction model. See Table 2 for definition of each code within each of the 4 dimensions.

10.2196/84712 Checklist 1 PRISMA checklist.

## Abbreviations

CFA: confirmatory factor analysis
PRISMA: Preferred Reporting Items for Systematic Reviews and Meta-Analyses
Q-SSP: Quality Assessment Checklist for Survey Studies in Psychology
RMSEA: root-mean-square error of approximation
SRMR: standardized root-mean-square residual
