# Supplementary material for: Questionnaires Used to Explore the Perspectives of Parents and Health Professionals on Young Children’s Use of Technology: Systematic Review
Source: JMIR Pediatr Parent. 2026 Jun 11;9:e84712. doi: 10.2196/84712 (PMC13256481; doi:10.2196/84712)
Supplement: Multimedia Appendix 1 [file pediatrics-v9-e84712-s001.docx]

**Table S1.** Core search concepts and related keywords.

|  | **Concept 1: Child** | **Concept 2: Technology** | **Concept 3: Perspectives + Adults** | **Concept 4: Questionnaire** |
| --- | --- | --- | --- | --- |
| **Keywords** | child* OR infan* OR toddler* OR preschool* OR pre-school* OR kindergarten* OR newborn* OR neonate* OR baby OR babies OR paediatric* OR pediatric* | tv OR television OR screen* OR comput* OR laptop* OR video* OR tablet* OR iPad OR DVD OR phone OR cellphone OR mobile OR media OR device* OR handheld OR console* OR smartphone* OR smart phone* OR interactive whiteboard* OR projector* OR digital sign* OR PC monitor* OR cinema OR camera* OR smartwatch* OR gaming OR gamer* OR electronic gam* OR e-gam* OR egam* OR Xbox OR PS4 OR PS5 OR digital toy* OR IoT OR playstation OR Wii OR virtual reality OR augmented reality OR internet OR Nintendo OR multitask* OR multi-task* OR watch* OR multiscreen* OR multi-screen OR technolog* OR web brows* OR social media OR facebook OR twitter OR Instagram OR TikTok OR snapchat OR youtube OR Netflix OR Disney plus OR hulu OR internet of toy* | perspect* OR attitude* OR belief* OR outlook OR approach* OR view* OR feeling* OR idea* OR perception* AND adult* OR parent* OR caregiver* OR care giver* OR mother OR father OR mum OR mom OR dad OR famil* OR health professional* | question* OR survey* OR diary OR self-report* OR selfreport* OR instrument* OR score OR scale* OR assess* OR measure* OR child report* OR parent report* |

**Table S2.** *Data-base specific adaptions for each core search concept.*

| **Database** | **Keywords** | **Database-specific subject headings** |
| --- | --- | --- |
| Medline (Mesh) | (Concept 1) ADJ5 (Concept 2) AND (Concept 3) ADJ5 AND (Concept 4) | Child, Preschool/ or exp Infant/;  Digital Technology/ or Multimedia/ or Sound Recordings/ or exp Television/ or social media/ or exp Video games/ or Cell Phone/ or Computers/ or exp Computer Simulation/;  Exp Attitude/ or exp Emotions/  ADJ5  Adult/ or Caregivers/ or Legal Guardians or exp Parents/ or exp Health Personnel/;  "Surveys and Questionnaires"/ OR Self Report/ |
| EMBASE | (Concept 1) ADJ5 (Concept 2) AND (Concept 3) ADJ5 AND (Concept 4) | preschool child/ or toddler/ or infant/;  digital technology/ or multimedia/ or audio recording/ or Television/ or exp social media/ or exp video game/ or exp mobile phone/ or computer/ or exp Computer Simulation/;  Attitude/ or exp emotion/ or perception/;  Adult/ or caregiver/ or legal guardian/ or exp parent/ or health care personnel/;  Exp questionnaire/ or self report/ |
| Psycinfo | (Concept 1) ADJ5 (Concept 2) AND (Concept 3) ADJ5 AND (Concept 4) | Exp Preschool Students/;  Exp Digital Technology/ or Multimedia/ or exp Television/ or exp social media/ or exp Computer Games/ or exp Cell Phone/ or Computers/ or exp Computer Simulation/ or exp Digital Media/;  Attitudes/ or Exp Adult Attitudes/ or exp Emotions/ or perception/ or exp Health Personnel Attitudes/;  Exp Caregivers/ or exp Parents/ or exp Health Personnel/;  Exp Surveys/ OR exp Self-Report/ |
| CINAHL | (Concept 1) N5 (Concept 2) AND (Concept 3) N5 AND (Concept 4) | MH Child, Preschool or MH Infant;  MH Digital Technology or MH Multimedia or or MH Television MH “social media+” (MH "Video Games") OR (MH "Exergames") OR (MH "Virtual Reality") or  (MH "Cellular Phone") OR (MH "Text Messaging") OR (MH "Smartphone") or MH “Computer Types”);  (MH "Attitude of Health Personnel+") OR (MH "Parental Attitudes+") OR (MH "Caregiver Attitudes") OR (MH "Attitude to Computers") OR (MH “emotions+”) OR MH Perception;  MH Adult OR (MH "Caregivers") OR (MH "Parents+") OR (MH ‘Health Personnel+”;  MH Surveys OR (MH “questionnaires+”) OR MH “self report” |
| SportDiscus | (Concept 1) N5 (Concept 2) AND (Concept 3) N5 AND (Concept 4) | DE "SUBSCRIPTION television" OR (DE "VIDEO games" OR DE "COMPUTER baseball games" OR DE "DANCE Dance Revolution (Game)" OR DE "EXERCISE video games" OR DE "SPORTS in video games" OR DE "VIDEO games & children");  DE "ATTITUDE (Psychology)" OR DE "EMOTIONS" ;  DE "CAREGIVERS" OR (DE "MEDICAL personnel" OR DE "NURSES") |
| WOS | (Concept 1) W/5 (Concept 2) AND (Concept 3) W/5 AND (Concept 4) | NA |
| Web of Science | (Concept 1) NEAR/5 (Concept 2) AND (Concept 3) NEAR/5 AND (Concept 4) | NA |
| ProQuest | (Concept 1) NEAR/5 (Concept 2) AND (Concept 3) NEAR/5 AND (Concept 4) | NA |

*NA = Not Applicable.*
