# Supplementary material for: Questionnaires Used to Explore the Perspectives of Parents and Health Professionals on Young Children’s Use of Technology: Systematic Review
Source: JMIR Pediatr Parent. 2026 Jun 11;9:e84712. doi: 10.2196/84712 (PMC13256481; doi:10.2196/84712)
Supplement: Multimedia Appendix 2 [file pediatrics-v9-e84712-s002.docx]

Table S1 provides the results of the quality assessment of included studies.

**Table S1.** Quality assessment of included studies, with questionable quality highlighted with shading.

| **Study** | **Overall**  **score** | **Introduction**  **score** | **Participant**  **score** | **Data score** | **Ethics score** |
| --- | --- | --- | --- | --- | --- |
| Akyol, 2022 (31) | 58 | 100 | 33 | 50 | 100 |
| Aladé and Donohue, 2022 (32) | 58 | 100 | 33 | 40 | 100 |
| Al-Balushi and Al-Shihi, 2016 (33) | 32 | 100 | 0 | 20 | 0 |
| Alkalash et al, 2023 (34) | 79 | 100 | 67 | 70 | 100 |
| AlSamhori et al, 2023 (35) | 61 | 100 | 67 | 33 | 100 |
| Amzalag, 2021 (36) | 42 | 100 | 0 | 40 | 0 |
| Arippin et al, 2023 (37) | 53 | 100 | 67 | 30 | 100 |
| Asplund et al, 2015 (38) | 74 | 100 | 33 | 70 | 100 |
| Balaban and Bayindir, 2019 (39) | 68 | 100 | 67 | 70 | 0 |
| Bansal et al, 2023 (40) | 47 | 100 | 33 | 20 | 100 |
| Barmomanesh et al, 2017 (41) | 47 | 100 | 67 | 30 | 0 |
| Beyens and Eggermont, 2014 (42) | 58 | 100 | 67 | 40 | 100 |
| Bleakley et al, 2013 (43) | 68 | 100 | 67 | 60 | 0 |
| Boonmun et al, 2023 (44) | 74 | 100 | 67 | 70 | 100 |
| Bourha et al, 2024 (45) | 53 | 100 | 0 | 40 | 100 |
| Brauchli et al, 2023 (46) | 84 | 100 | 33 | 90 | 100 |
| Brown et al, 2023 (47) | 74 | 100 | 33 | 80 | 0 |
| Cardy et al, 2023 (48) | 72 | 100 | 67 | 56 | 100 |
| Carson et al, 2012 (49) | 84 | 100 | 67 | 80 | 100 |
| Carson et al, 2013 (50) | 68 | 100 | 67 | 50 | 100 |
| Chattha et al, 2021 (51) | 53 | 50 | 67 | 40 | 100 |
| Chia et al, 2022 (52) | 79 | 100 | 67 | 70 | 100 |
| Chen and Tu, 2018 (53) | 47 | 100 | 0 | 40 | 100 |
| Cingel and Krcmar, 2013 (54) | 74 | 100 | 67 | 60 | 100 |
| Covolo et al, 2021 (55) | 74 | 100 | 67 | 60 | 100 |
| Dardanou et al, 2020 (56) | 37 | 100 | 0 | 20 | 0 |
| Dong et al, 2022 (57) | 63 | 100 | 67 | 60 | 0 |
| Eales et al, 2021 (58) | 74 | 100 | 67 | 70 | 0 |
| Ebbeck et al, 2016 (59) | 45 | 100 | 33 | 30 | 50 |
| Fan et al, 2022 (60) | 74 | 100 | 67 | 60 | 100 |
| Farima et al, 2023 (61) | 42 | 100 | 33 | 10 | 100 |
| Garcia-Conde et al, 2020 (62) | 58 | 75 | 67 | 50 | 0 |
| Gjelaj et al, 2020 (63) | 32 | 75 | 33 | 20 | 0 |
| González-Sanmamed et al, 2023 (64) | 53 | 100 | 0 | 50 | 0 |
| Grané et al, 2023 (65) | 61 | 100 | 33 | 44 | 100 |
| Griffith et al, 2023 (66) | 79 | 100 | 67 | 80 | 0 |
| Halpin et al, 2021 (67) | 89 | 100 | 100 | 80 | 100 |
| Hamilton et al, 2016 (68) | 53 | 100 | 67 | 40 | 0 |
| Hatzigianni et al, 2014 (69) | 37 | 75 | 33 | 20 | 100 |
| Howie et al, 2020 (70) | 68 | 100 | 67 | 50 | 100 |
| Hutton et al, 2018 (71) | 68 | 100 | 33 | 60 | 100 |
| Ihmeideh and Alkhawaldeh,2017 (72) | 74 | 100 | 67 | 70 | 100 |
| Ilgar and Karakurt, 2018 (73) | 37 | 100 | 33 | 20 | 0 |
| Istenic et al, 2023 (74) | 58 | 100 | 0 | 50 | 100 |
| Istenic et al, 2023b (75) | 58 | 100 | 0 | 50 | 100 |
| Jain et al, 2023 (76) | 100 | 100 | 100 | 100 | 100 |
| Jin, 2013 (77) | 63 | 100 | 67 | 60 | 0 |
| Joginder Singh et al, 2021 (78) | 63 | 100 | 33 | 50 | 100 |
| Konok et al, 2020 (79) | 74 | 100 | 67 | 60 | 100 |
| Kostyrka-Allchorne et al, 2017 (80) | 42 | 100 | 33 | 30 | 0 |
| Lee et al, 2022 (81) | 58 | 100 | 33 | 50 | 0 |
| Lepicnik et al, 2013 (82) | 58 | 100 | 33 | 40 | 100 |
| Li and Chen, 2015 (83) | 37 | 100 | 0 | 30 | 0 |
| Liibaan et al, 2023 (84) | 32 | 75 | 33 | 20 | 0 |
| Little, 2019 (85) | 58 | 100 | 33 | 50 | 0 |
| Luo et al, 2023 (86) | 68 | 100 | 67 | 50 | 100 |
| Mansor et al, 2021 (87) | 74 | 100 | 67 | 60 | 100 |
| Matziou et al, 2021 (88) | 63 | 100 | 33 | 60 | 100 |
| Mikelic Preradovic et al, 2016 (89) | 42 | 100 | 33 | 30 | 0 |
| Milford et al, 2022 (90) | 68 | 100 | 67 | 50 | 100 |
| Nabi and Krcmar, 2016 (91) | 74 | 100 | 67 | 70 | 0 |
| Natsiopoulou et al, 2013 (92) | 32 | 100 | 33 | 10 | 0 |
| Nikken, 2019 (93) | 68 | 100 | 67 | 50 | 100 |
| Nikken and Schols, 2015 (94) | 58 | 100 | 67 | 50 | 0 |
| Njoroge et al, 2013 (95) | 74 | 100 | 67 | 60 | 100 |
| Nwankwo et al, 2019 (96) | 58 | 100 | 67 | 30 | 100 |
| O’Connor and Fotakopoulou, 2016 (97) | 68 | 100 | 67 | 50 | 100 |
| Ophir et al, 2023 (98) | 58 | 100 | 0 | 50 | 100 |
| Petegem et al, 2019 (99) | 58 | 100 | 67 | 40 | 50 |
| Raj et al, 2022 (100) | 89 | 100 | 100 | 80 | 100 |
| Raj et al, 2023 (101) | 89 | 100 | 100 | 80 | 100 |
| Rajalakshmi et al, 2023 (102) | 63 | 100 | 67 | 40 | 100 |
| Rosanda et al, 2022 (103) | 47 | 100 | 0 | 50 | 0 |
| Sada Garibay and Lapierre, 2024 (104) | 60 | 100 | 67 | 50 | 0 |
| Seršen et al, 2024 (105) | 53 | 100 | 33 | 40 | 0 |
| Solomon-Moore et al, 2017 (106) | 63 | 100 | 33 | 50 | 100 |
| Stuckelman et al, 2023 (107) | 58 | 100 | 67 | 40 | 0 |
| Suresh and Tiwari, 2023 (108) | 79 | 100 | 67 | 70 | 100 |
| Tanusha et al, 2023 (109) | 63 | 100 | 67 | 50 | 100 |
| Tay et al, 2021 (110) | 58 | 100 | 67 | 50 | 0 |
| Vaala and Hornik, 2014 (111) | 47 | 75 | 33 | 40 | 0 |
| Vaiopoulou at al, 2021 (112) | 45 | 100 | 33 | 20 | 50 |
| Vittrup et al, 2016 (113) | 68 | 100 | 67 | 60 | 0 |
| Vincent et al, 2021 (115) | 68 | 100 | 67 | 60 | 50 |
| Wang et al, 2024 (114) | 50 | 75 | 33 | 33 | 100 |
| **Overall mean scores (range)** | **61**  **(32-100)** | **98**  **(50-100)** | **49**  **(0-100)** | **49**  **(10-100)** | **60**  **(0-100)** |

*Scores calculated as the ratio between “yes” scores and total applicable quality assessment items. For the calculation of overall score, a score of ≥73% may be considered acceptable quality. The threshold for acceptable quality for each domain were ≥75% for introduction; ≥67% for participants; ≥70% for data and ≥100% for ethics. Cells shaded in the table indicates studies that did not meet this threshold.*
