# Supplementary material for: Questionnaires Used to Explore the Perspectives of Parents and Health Professionals on Young Children’s Use of Technology: Systematic Review
Source: JMIR Pediatr Parent. 2026 Jun 11;9:e84712. doi: 10.2196/84712 (PMC13256481; doi:10.2196/84712)
Supplement: Multimedia Appendix 3 [file pediatrics-v9-e84712-s003.docx]

**Table S1.** Characteristics and psychometric properties of questionnaire used.

| **Author, year, country** | **Questionnaire details (title, item types, administration format)** | **Questionnaire development** | **N of items (total and related to perspective) and scoring details** | **Questionnaire psychometric properties^A^** |
| --- | --- | --- | --- | --- |
| Akyol, 2022, Turkey, UMIC (31) | Questionnaire titled “Parents’ Views of Preschool Children’s Technology Use Scale”.  Questionnaire comprised closed-ended questions.  Questionnaire was delivered and fill-out by parents via Google Forms. | Questionnaire was developed by Kilinc (2015) (116) and reported in an unpublished master thesis. | Total number of items: 25.  Number of items related to perspectives NR.  Items were rated on a 5-point Likert scale, where 1= strongly disagree and 5=strongly agree. | Internal consistency reported as Cronbach’s alpha which was 0.79. Unclear if this was for the total questionnaire or items related to perspectives. |
| Aladé and Donohue, 2022, USA, HIC (32) | Title NR. Questionnaire compromised open- and closed-ended items. Questionnaire was emailed to parents by school personnel on behalf of the researchers. The questionnaire was filled-out online and a reminder to complete the survey was sent out two weeks after the initial email. | Questionnaire was developed specifically for this study. | Total number of items NR.  Items related to perspectives: 10.  Items were scored using a 4-point Likert scale, where 1 = strongly agree and 4 = strongly disagree. | Internal consistency reported as Cronbach’s alpha. This was 0.67 for four of the five items used to measure attitudes toward mobile device use at home and 0.70 for all five items used to measure attitudes toward mobile device use at school. |
| Al-Balushi and Al-Shihi, 2016, Oman, HIC (33) | Title NR. Questionnaire comprised closed-ended questions. Administration format NR. | Questionnaire was developed specifically for this study based on a literature review. | Total number of items NR.  Items related to perspectives: 10.  Scoring details NR. | Face validity was evaluated by conducting a pilot study where 10 parents fill-out the questionnaire.  Content validity was evaluated by asking experts whether the questions effectively captured the topic under investigation.  Questionnaire was refined based on comments from experts and analysis of survey responses. |
| Alkalash et al, 2023, Saudi Arabia, HIC (34) | Title NR. Questionnaire compromised closed-ended items. Questionnaire was distributed to parents in selected schools in the western Saudi Arabia via WhatsApp and Telegram groups and to visitors to primary healthcare centres. | Questionnaire was developed specifically for this study. | Total number of items: 47.  Items related to perspectives: 6.  Items were scored using a 3-point Likert scale, where 1= agree and 3= disagree.  Attitudes were dichotomized into two categories (agree=1 point, neutral or disagree=0 points). A positive attitude was considered if the parents had a score of 60% or more. | Face validity was evaluated by conducting a pilot study of 26 parents to test the language clarity and question understandability.  Internal consistency reported as Cronbach’s alpha which was 0.89 for all items in the questionnaire. |
| AlSamhori et al, 2023, Jordan, UMIC (35) | Title NR. Questionnaire compromised closed-ended items. Questionnaire was distributed to parents and filled-out online. No further details provided on administration format. | Questionnaire was adopted from previous studies (117-120). The questionnaire was structured in English, translated to Arabic and translated back from English to Arabic. | Total number of items: 43.  Items related to perspectives: 2.  Items were marked ‘yes’ or ‘no’ for which they agreed with the statement. | NR. |
| Amzalag, 2021, Isreal, HIC (36) | Title NR. Questionnaire compromised close-ended items. Questionnaire distributed online through WhatsApp and fill-out using Google Forms. | Questionnaire items were based on two previous questionnaires (121) and adapted to the current study. | Total number of items NR.  Items related to perspectives: 24.  Items were scored using a 5-point Likert scale, where 1=do not agree at all, and 5=agree to a large extent. | Internal consistency was evaluated using Cronbach’s alpha, which ranged between 0.80-0.83 for items related to perspectives. |
| Arippin et al, 2023, Brunei Darussalam, HIC (37) | Title NR. Questionnaire compromised closed-ended items. Questionnaire was filled-out online. No further details provided on administration format. | Questionnaire was developed specifically for this study. | Total number of items NR.  Items related to perspectives: 21.  Frequency and percentage provided when reported results related to parents’ perspectives, but no information reported on how these measures were calculated or how items were scored. | Face validity was evaluated by conducting a pilot test among 5 mothers after which the questionnaire was modified. |
| Asplund et al, 2015, USA, HIC (38) | Title NR. Questionnaire compromised closed-ended items. Administration format NR. | Questionnaire items were adapted from the National Survey of Children’s Health 2007 and 2011, Viner and Cole’s child TV-viewing scale (2005) (122) and Pearson’s scale of restrictive parental practices regarding TV use (2011) (123). Items were translated to Spanish by a staff member trained in medical translation. | Number of items NR.  Items were scored using a 5-point Likert scale to report agreement/disagreement. Scale endpoints NR. | NR. |
| Balaban and Bayindir, 2019, Turkey, UMIC (39) | Questionnaire titled “Effects of Digital Games in Early Ages Scale”. Questionnaire compromised closed-ended items. Questionnaire was distributed to families at schools by the teachers. | Questionnaire was developed specifically for this study. | Total number of items: 25.  Items related to perspectives: 17.  Items were scored using a 5-point Likert scale, where 1 = never and 5 = always. | Content validity was evaluated by consulting 9 experts within the field.  Construct validity was evaluated using principal component factor analysis. The factor loading values of the final 25-item scale were between 0.54 and 0.87.  Internal validity was evaluated using Cronbach’s alpha. This was 0.87 for the overall scale, ranging between 0.82-0.90 for each sub-domain.  Test-retest reliability was evaluated among 46 children, which showed no statistical difference in mean scores over two weeks. |
| Bansal et al, 2023, India, LMIC (40) | Title NR. Questionnaire compromised closed-ended items. Administration format NR. | Questionnaire was predesigned. | Total number of items NR.  Items related to perspectives: 21.  Frequency and percentage provided when reported results related to parents’ perspectives, but no information reported on how these measures were calculated or how items were scored. | NR. |
| Barmomanesh et al, 2017, New Zealand, HIC (41) | Title NR. Questionnaire comprised open- and closed-ended items. Questionnaire delivered and fill-out as an online survey. | Questionnaire was developed specifically for this study. | Number of items NR.  The items related to parents’ perceptions were mostly multiple choice with optional text field.  Numerical analysis was used to analyse closed-end questions; open coding method was used to analyse open-ended questions. | NR. |
| Beyens and Eggermont, 2014, Belgium, HIC (42) | Title NR. Questionnaire comprised closed-ended items. Questionnaire was sent home with children to parents who had volunteered to participate in the study, and was collected in kindergartens and child-care centres after completion. | Questionnaire was developed specifically for this study and based on items used in previous studies (124,125). | Total number of items NR.  Items related to perspectives: 7.  Parental attitude toward television assessed by asking parents to indicate their attitude toward seven positive outcomes of television among young children using a 5-point Likert scale, where 1 = strongly disagree and 5 = strongly agree.  A summary score of the seven items were used. | Internal validity was evaluated using Cronbach’s alpha. This was 0.773 for the seven items used to generate a summary score for parental attitude. |
| Bleakley et al, 2013, USA, HIC (43) | Title NR. Telephone survey conducted based on open- and closed-ended questions. Administration format NR. | Questions were developed specifically for this study. | Total number of items NR.  Items related to perspectives: 6.  Parents’ attitudes were measured by six items (complicated/simple, bad/good, foolish/wise, unpleasant/pleasant, easy/difficult, harmful/beneficial) in an ordinal measure ranging from –3 to 3. The six items were then averaged to create a scale. | Internal validity was evaluated using Cronbach’s alpha. This was 0.79 (CI=0.88; 1.12) for the six items used to generate a summary score for parental attitude. |
| Boonmun et al, 2023, Thailand, UMIC (44) | Title of the questionnaire regarding parents’ perspectives was “The Parents’ Attitudes regarding Children’s Screen  Time Questionnaire”. Questionnaire compromised closed-ended items. Parents filled-out the questionnaire at three timepoints: before receiving the intervention; one week after the intervention; and two months after the intervention. | Questionnaire was developed specifically for this study. | Total number of items: NR.  Items related to perspectives: 18.  Items were scored using a 5-point Likert scale, where 1 = disagree and 5 = strongly disagree.  A higher score indicated a more positive attitude towards children’s screen time reduction. | Content validity was evaluated by five experts from different areas. For the questionnaire measuring parents’ attitudes regarding children’s screen time, content validity index was reported to be 1.00.  Internal validity was evaluated using Cronbach’s alpha which was reported to be 0.84 in the pilot study and 0.81 in the main study. |
| Bourha et al, 2024, Greece, HIC (45) | Title NR. Pre-questionnaire (i.e. before child’s interaction with technology-enhanced toys) compromised closed-ended items.  Post-questionnaire (i.e. post to child’s interaction with technology-enhanced toys) compromised closed-ended and open-ended items. Parents were given a link to fill-out the questionnaires online. | Questionnaire was developed based on two previous studies (126,127). | Total number of items: 10 (pre-questionnaire) & 20 (post-questionnaire).  Items related to perspectives: 1 (pre-questionnaire) & 5 (post-questionnaire; 2 closed-ended and 3 open-ended).  For both pre- and post-questionnaires, the first item was scored using a 5-point Likert scale, where 1 = almost not significant and 5 = extremely significant.  The second item was answered using multiple-choice options. | NR. |
| Brauchli et al, 2023, Switzerland, HIC (46) | Title NR. Questionnaire compromised closed-ended items. Questionnaire was filled-out online by sending an individualised link to the parents at four time points. A reminder email was sent to parents if they had not completed the questionnaire within a day. | Questionnaire developed specifically for this study. | Total number of items: 11  Items related to perspectives: 3.  Items were again rated on a 6-point Likert scale, where 1 = not true and 6 = true. | McDonald’s omega was reported as measure for internal consistency for the items related to perspectives which was 0.62, 0.65, 0.66 and 0.69 at T1/2/3/4. |
| Brown et al, 2023, USA, HIC (47) | Title NR. Questionnaire compromised open- and closed-ended items. Questionnaire was delivered and filled-out in paper form. | Questionnaire was developed specifically for this study based on items used in previous studies (128-131). Items were available in English and Spanish. | Total number of items: 59.  Items related to perspectives: 2.  For the items related to perspectives, parents were asked to choose up to 3 most important options or pick “none”.  Results reported as top identified positive and negative views of childhood digital media use. | Face validity was evaluated by conducting a pilot test among a subset of parents. |
| Cardy et al, 2023, Canada, HIC (48) | Title NR. Questionnaire comprised closed-ended questions. Questionnaire was admitted using an anonymous electronic system hosted at a hospital | Questionnaire was developed specifically for this study in consultation with parents of autistic children through an iterative feedback and refinement process. | Total number of items: 44.  Items related to perspectives: 2.  Scoring details NR. | NR. |
| Carson et al, 2012, Canada, HIC (49) | Title NR. Questionnaire compromised closed-ended items. The questionnaire was delivered to eligible parents in a package and could be filled-out and returned in a prepared envelope or could be completed online. | Questionnaire was developed specifically for this study based on items used in previous studies. The questionnaire had been pilot tested and for some of the items within a section, details were provided on how these had been developed in previous studies. | Total number of items: 30.  Items related to perspectives: 8.  Items related to perspectives were rated on a 4-point Likert scale, where 1=strongly disagree and 4=strongly agree.  Responses were averaged to create an overall ‘attitude score’, with higher scores reflecting more positive attitudes. | Internal validity was evaluated using Cronbach’s alpha which was 0.84 for the items related to perspectives. |
| Carson et al, 2013, Canada, HIC (50) | Title NR. Questionnaire compromised closed-ended items. The questionnaire was delivered to eligible parents in a package. | Questionnaire was developed specifically for this study based on items used in previous studies (132-134). | Total number of items: 17.  Items related to perspectives: 15.  Items related to perspectives were rated on a 4-point Likert scale, where 1=strongly disagree and 4=strongly agree.  Responses were dichotomized into agree and disagree. | NR. |
| Chattha et al, 2021, Pakistan, LMIC (51) | No title nor details about the questionnaire was reported. | No details reported on questionnaire development. | Number of items NR.  Frequency and percentage provided when reported results related to parents’ perspectives, but no information reported on how these measures were calculated or how items were scored. | NR. |
| Chia et al, 2022, Singapore, HIC (52) | Questionnaire titled “Surveillance of Digital Media Habits in Early Childhood Questionnaire (SMALLQ®)”. Not reported if items were open or closed-ended. Questionnaire was delivered and fill-out as an online survey. | Questionnaire was developed in a previous study based on a 7-step approach (135). | Total number of items NR.  Number of items related to perspectives: 12.  Items related to perspectives were rated on a 5-point Likert scale, where 1=not important/not concerned and 5=very important/very concerned.  Responses were dichotomized into not very important/not very concerned and very important/very concerned. | Content validity was evaluated by consulting four experts within the field.  Face validity was evaluated by inviting 5 parents to provide input on the draft questionnaire.  Internal validity was evaluated using Cronbach’s alpha, which was 0.78 for the physical activity dimension, 0.74 for the screen time dimension 0.74, and 0.69 for the sleep dimension. |
| Chen and Tu, 2018, Taiwan, HIC (53) | Questionnaire titled “Use of the Internat and Preschool Education (UIPE) Attitude Survey”. Questionnaire compromised closed-ended items. Administration format NR. | Questionnaire was developed specifically for this study. | Total number of items in the questionnaire: 18.  Number of items related to perspectives: 4.  Items were rated on a 5-point Likert scale, where 1=strongly disagree and 5=strongly agree. | Content validity was evaluated by consulting experts within the field.  Construct validity was evaluated using a confirmatory factor analysis on five latent constructs based on all items: root mean square error of approximation = 0.06, root mean square residual = 0.03 and standardized root mean square residual = 0.05.  Reliability and convergent validity of the five latent constructs were evaluated by assessing the composite reliability and average variance extracted. For items related to perspective, composite reliability was 0.88 and average variance extracted was 0.66. |
| Cingel and Krcmar, 2013, USA, HIC (54) | Title NR. Not reported if items were open or closed-ended. Questionnaire was delivered and fill-out to parents either in paper format in daycare centres or as an online survey. | Questionnaire was developed for this study. | Total number of items NR.  Items related to perspectives: 12 items related to perceived positive effects of digital media. Number of items used to measure perceived negative beliefs about digital media was not reported.  Items were rated on a 7-point Likert scale, where 1=strongly disagree and 7=strongly agree.  Items were averaged to calculate an overall score for parents’ positive and negative beliefs. | Internal validity was evaluated using Cronbach’s alpha. This was 0.92 for items used to derive the positive belief score and 0.82 for items used to derive the negative belief score. |
| Covolo et al, 2021, Italy, HIC (55) | Title NR. Questionnaire compromised open- and closed-ended items. Questionnaire delivered and fill-out as an online survey. | Questionnaire was developed specifically for this study. | Total number of items: 26.  Items related to perspectives: 4.  Items were measured on a 7-point Likert scale, where 1=no risk or benefit and 7=high risk or high benefit.  Scores were categorised into no risk/benefit, low risk/benefit, high risk/benefit. | Face validity was evaluated by conducting a pilot test among a small group of people (~20). |
| Dardanou et al, 2020, Norway, Portugal and Japan, HIC (56) | Translated and adapted version of a questionnaire titled “Technobabies Questionnaire for Parents/Carers with child under 36 months”*.* Questionnaire compromised open- and closed-ended items.  Questionnaire was delivered and fill-out as an online survey. | Questionnaire was translated and adapted to be culturally valid from an existing survey. | Total number of items: 17.  Items related to perspectives: 2.  Mean and standard deviation provided when reporting results related to parents’ perspectives, but no information reported on how these measures were calculated or how the items were scored. | NR. |
| Dong et al, 2022, China, UMIC (57) | Questionnaire titled “Home Digital Practices Survey (HDPS)”. Questionnaire compromised open- and closed-ended items. Questionnaire was delivered and fill-out as an online survey. | Questionnaire was developed for this study. | Total number of items: 28.  Items related to perspectives: 6.  Items were measured on a 6-point Likert scale, where 1=strongly disagree and 6=strongly agree. | Construct validity was evaluated using a confirmatory analysis and results showed that 42.57% of the total variation could be explained by the five-construct model. The five constructs’ eigenvalues and explained percentages were 13.18 (18.56%), 6.36 (8.96%), 4.54 (6.40%), 3.22 (4.54%), and 2.92 (4.11%).  Internal validity was evaluated using Cronbach’s alpha, which ranged between 0.781 and 0.918 for the five constructs. |
| Eales et al, 2021, USA, HIC (58) | Questionnaire titled “The Common Sense Census (CSC) of children's media use”. The questionnaire compromised open- and closed ended questions. Questionnaire was delivered and filled-out as an online survey. | Questionnaire was developed specifically for this study based on items used in a previous study (128). | Total number of items not reported.  Number of items related to perspectives: 6.  Items were measured on a 5-point Likert scale, where 1=helps a lot and 5=hurts a lot. Means were calculated for each timepoint. | Internal validity was evaluated using Cronbach’s alpha, which was 0.78 at timepoint 1 and 0.70 at timepoint 2 for items related to perspectives. |
| Ebbeck et al, 2016, Singapore, HIC (59) | Title NR. The questionnaire comprised open- and closed-ended questions. Parents/caregivers filled-out a paper version and returned it to a collection box at a childcare centre within 2 weeks. | Questionnaire was developed specifically for this study. | Number of items NR.  Results reported as frequency count, but no details on scoring were provided. | Face validity was evaluated by conducting a pilot test using a “think-aloud technique”,”, and the participants reported that no changes to the questions were necessary. |
| Fan et al, 2022, China, UMIC (60) | Title NR. The questionnaire compromised open and closed-ended. Questionnaire was delivered and filled-out as an online survey. | Questionnaire was developed based on a previous study (136). | Total number of items: 17.  Items related to perspectives: 1.  Results reported as percentage, but no details on scoring were provided. | NR. |
| Farima et al, 2023, Moldova, UMIC (61) | Title NR. Questionnaire compromised closed-ended items. Administration format NR. | Questionnaire was developed specifically for this study based on a previous qualitative study and international recommendations of children’s technology use. | Total number of items NR.  Items related to perspectives NR.  Scoring details NR. | Face validity was evaluated by conducting a pilot test among 15 people, after which the questionnaire was adjusted. |
| Garcia-Conde et al, 2020, Spain, HIC (62) | Title NR. Not reported if items were open or closed-ended. Questionnaire was given to children by teachers who took it home to be filled-out by their parents. | Questionnaire based on previous studies (137). | Total number of items not reported.  Number of items related to perspectives: 2.  Items were measured on a 5-point Likert scale, ranging from 1=none to 5=very much. | Internal validity was evaluated using Cronbach’s alpha which as 0.24 for items related to perspectives. |
| Gjelaj et al, 2020, Kosovo, UMIC (63) | Title NR. Not reported if items were open or closed-ended. Questionnaire was delivered and filled-out as an online survey. | Questionnaire was developed specifically for this study. | Number of items NR.  Results reported as percentage, but no details on scoring were provided. | NR. |
| González-Sanmamed et al, 2023, Spain, HIC (64) | Title NR. Questionnaire compromised closed-ended items. Parents filled out questionnaires on their mobile phones using a QR code provided to them at their children’s school. | Questionnaire was developed specifically for this study. | Total number of items NR.  Items related to perspectives 15.  Items were again rated on a 5-point Likert scale, where 1 = never and 5 = always. | Content validity was evaluated by consulting eight experts within the field.  Construct validity was evaluated using Exploratory Factorial Analysis based on parallel analysis. All items in the questionnaire had a factor loading above 0.52 (range: 0.52-0.90).  Internal validity was evaluated using Cronbach’s alpha which was 0.89 for items related to perspectives. |
| Grané et al, 2023, Spain, HIC (65) | Title NR. Questionnaire compromised closed and open-ended items. Parents filled-out the questionnaire with two researchers guiding them through the questions. | Questionnaire was developed specifically for this study based on a systematic literature review and a previous study (138). | Total number of items: 30.  Items related to perspectives: 5.  Scoring details NR. | Content validity was evaluated by consulting experts within the field, after which the questionnaire was revised.  Face validity was evaluated by pilot testing the questionnaires among four families. |
| Griffith et al, 2023, USA, HIC (66) | Title NR. Questionnaire compromised closed-ended items. Questionnaires were filled-out online using a link sent to the parents via email. | Questionnaire was developed specifically for this study. | Total number of items NR.  Items related to perspectives: 8.  Items were again rated on a 5-point Likert scale, where 1 = hurts a lot and 5 = helps a lot.  A mean score was calculated, with higher scores indicating a more positive attitude. | Internal validity was evaluated using Cronbach’s alpha, which was 0.84 for the items related to perspectives. |
| Halpin et al, 2021, Australia, HIC (67) | Questionnaire titled “Effects of Children’s Screen Time Scale (ECSTS)”. Questionnaire compromised of closed-ended items. Questionnaire was delivered and filled-out as an online survey. | Questionnaire was developed in a previous study (139). | Total number of items: 23.  Number of items related to perspectives: 23.  Responses were measured on a 11-point Likert scale, where -5=extremely negative and +5=extremely positive. Raw scores were converted to item scores between 0-10. A mean score was calculated to give an overall score of how positively or negatively the participant perceived their child’s screen use, with higher scores indicating more positive effects. | Internal validity was evaluated using Cronbach’s alpha, which was 0.94 for the complete questionnaire. |
| Hamilton et al, 2016, Australia, HIC (68) | Title NR. Questionnaire compromised closed-ended items. Questionnaire was filled-out and returned either as an online survey or in paper format at baseline. | Questionnaire was developed based on previous studies. No further information provided on questionnaire development. | Total number of items NR.  Number of items related to perspectives: 10.  Participants were asked to rate how likely the costs (i.e., ‘Increase parent distress’) and benefits (i.e., ‘Improve my child’s mental wellbeing’) would result if they performed the target behaviour (i.e. screen time). Responses ranged from [1] extremely unlikely to [7] extremely likely.  Results reported as means and standard deviation. | NR. |
| Hatzigianni et al, 2014, Australia, HIC (69) | Title NR. Questionnaire compromised of closed- and open-ended items. Parents filled-out a paper version of the questionnaire which was returned to teachers or a researcher. Data were collected pre and post a seven-month intervention. | Questionnaire was developed specifically for this study. No further information provided on questionnaire development. | Number of items NR.  Results reported as frequency count and percentage, but no details on scoring were provided. | NR. |
| Howie et al, 2020, Australia & USA, HIC (70) | Questionnaire titled “The Technology Use Questionnaire (TechU-Q)”. Not reported if items were open or closed-ended. Questionnaire was filled-out and returned as an online survey via email and hard copies were available upon request. | Questionnaire was developed based on author expertise on the topic and existing published surveys of child technology use.  Items assessing parents’ perceptions of technology use were added to the questionnaire for this specific study. | Total number of items not reported.  Total number of items related to perspectives: 11.  Parents’ perspectives were scored on a 5-point Likert scale, where 1=strongly disagree and 5=strongly agree. | Internal validity was evaluated using Cronbach’s alpha, which was 0.80 for the complete questionnaire. |
| Hutton et al, 2018, USA, HIC (71) | Title NR. Questionnaire compromised closed-ended items. Administration format not reported, but data were collected at two home visits (third trimester at approximately 2 months of age). | Questionnaire was developed based on previous studies, reviewed by experts and designed at the author’s institution. | Total number of items NR.  Number of items related to perspectives: 1.  Item was measured on a 5-point Likert scale, where 1=not important and 5=extremely important. | Face validity was evaluated by conducting a pilot test among colleagues and a convenience sample of low socioeconomic status mothers. |
| Ihmeideh and Alkhawaldeh, 2017, Jordan, UMIC (72) | Title NR. Questionnaire compromised closed-ended items. The questionnaires were distributed to the teachers and parents by research assistants and were collected one week after distribution. | Questionnaire was developed specifically for this study. The items were prepared after a comprehensive review of the literature. They were first written in Arabic and then translated into English by the authors. | Total number of items in the questionnaire: 26  Number of items related to perspectives: 26.  Items were rated on a 5-point Likert scale, where 1=strongly disagree and 5=strongly agree. | Content validity was evaluated by a consulting experts within the field.  Face validity was evaluated by conducting a pilot test among 20 preschool teachers and 20 parents from the study population outside the original study sample.  Internal validity was evaluated using Cronbach’s alpha, which ranged between 0.70 and 0.81. |
| Ilgar and Karakurt, 2018, Turkey, UMIC (73) | Title NR. Questionnaire compromised closed-ended items. Administration format was not reported. | Questionnaire was developed specifically for this study based on literature review. | Total number of items in the questionnaire: 21.  Number of items related to perspectives: 21.  Items were marked ‘yes’ or ‘no’.  Frequency and percentage provided when reporting results related to mothers’ perspectives. | Content validity was evaluated by a consulting experts within the field.  Face validity was evaluated by conducting a pilot study with a group meeting similar criteria to those in the study sample. |
| Istenic et al, 2023, Slovenia, HIC (74) | Title NR. Questionnaire compromised closed-ended items. Questionnaire was delivered and fill-out as an online survey. | Questionnaire was developed specifically for this study based on literature review. | Total number of items was not reported.  Number of items related to perspectives: 6.  Items were marked ‘yes’ or ‘no’ for which they agreed with the statement. | Internal validity was evaluated using Cronbach’s alpha, which ranged between 0.79 and 0.92 for items related to perspectives. |
| Istenic et al, 2023b, Slovenia, HIC (75) | Title NR. Questionnaire compromised closed-ended items. Questionnaire was delivered and fill-out as an online survey. | Questionnaire was developed specifically for this study based on literature review. | Total number of items NR.  Number of items related to perspectives: 28.  Means and standard deviation reported in results, but no scoring details were provided. | Internal validity was evaluated using Cronbach’s alpha, which ranged between 0.79 and 0.92 for the items related to perspectives. |
| Jain et al, 2023, India, LMIC (76) | Title NR. Questionnaire compromised of closed- and open-ended items. Parents filled in a paper version of the questionnaire during an initial household visit. Filled-in questionnaires were collected by research assistants at a second household visit. Research assistants interviewed the parent and/or the child to fill-out incomplete questionnaires.  Children between 10 and 15 years of age completed the questionnaire themselves. | Questionnaire was developed specifically for this study through literature review by two subject experts. | Total number of items in questionnaire: 20.  Total number of items related to perspectives: 3.  Results reported as frequency count and percentage, but no details on scoring were provided. | Face validity was evaluated by conducting a pilot study among 30 children aged 3-15 years.  Content validity was evaluated in terms of content validity ratio (CVR) and content validity index (CVI).  CVR was defined as number of experts who assessed an item to be essential divided by number of experts (n=3). Total CVR was 17.33.  CVI was defined as the CVR divided by total number of items (n=20). CVI of the questionnaire was 0.867. |
| Jin, 2013, Korea, HIC (77) | Title NR. Questionnaire compromised both open- and closed-ended items. Questionnaire was delivered and fill-out as an online survey. | Questionnaire was developed specifically for this study based on a review of the literature and previously developed items, which were modified. | Total number of items in the questionnaire: 26  Number of items related to perspectives: 3.  Items were rated on a 5-point Likert scale, where 1=strongly disagree and 5=strongly agree. | Face validity was evaluated by conducting a pilot test among to 35 panel members.  Reliability was measured at 0.7 for items related to parents’ perspectives on their children’s internet usage, but the measure used to test reliability was not reported. |
| Joginder Singh et al, 2021, Malaysia, UMIC (78) | Title NR. Questionnaire compromised both open- and closed-ended items. Questionnaire was delivered and fill-out as an online survey. | Questionnaire was developed specifically for this study based on literature review. | Total number of items was NR.  Number of items related to perspectives: 1.  Items were rated on a 5-point Likert scale, where 1=strongly agree and 5=strongly disagree.  Results reported as percentage. | Face validity was evaluated by conducting a pilot test among 10 parents. |
| Konok et al, 2020, Hungary, HIC (79) | Questionnaire titled “Digital Kids Questionnaire”. Questionnaire compromised closed-ended items. Questionnaire was delivered and fill-out as an online survey. | Questionnaire was based on an earlier open-ended survey completed by 96 parents of children aged between 0 and 10 years. | Total number of items NR.  Number of items related to perspectives: 33.  Items of beliefs about harms/benefits were marked as “Yes’ or “No”.  Parental attitude towards children’s mobile touch screen device usage was based on agreement with if it is better for a child to start the use “as late as possible” (negative attitude, 1 point) or “as early as possible” (positive attitude, 3 point), or with neither (neutral/mixed attitude, 2 point). | NR. |
| Kostyrka-Allchorne et al, 2017, UK, HIC (80) | Title NR. Questionnaire compromised closed-ended items. Parents completed the questionnaires at home and returned the forms to the school office or a preschool manager. | Questionnaire was adopted from a previous study. | Total number of items in the questionnaire: 10.  Number of items related to perspectives: 2.  Items were rated on a 5/6-point Likert scale, where 0=Not harmful at all/ Very negative and 4=Extremely harmful or 5=Very positive. | NR. |
| Lee et al, 2022, Korea, HIC (81) | Title NR. Questionnaire compromised closed-ended items Questionnaire was delivered and fill-out as an online survey. | Questionnaire was adapted from a previous study and was translated from English to Korean. Translation was checked by researchers who were fluent in both languages. | Total number of items NR.  Number of items related to perspectives: 17.  Items were rated on a 5-point Likert scale, where 1=strongly disagree and 5=strongly agree. | Reliability reported as 0.89 for measures of positive attitudes towards children’s media use. For both negative attitudes towards media usage influence on intellectual and social domains, reliability reported as 0.84. No information provided on how the reliability measures were obtained. |
| Lepicnik et al, 2013, Slovenia, HIC (82) | Title NR. Questionnaire compromised closed-ended items Administration format NR. | Questionnaire was developed specifically for this study based on a literature search. | Total number of items NR.  Items related to perspectives: 1.  Frequency and percentage provided when reporting results related to parents’ perspectives, but no scoring details reported. | Face validity was evaluated by testing a draft of the questionnaire, but no details were provided on how this was conducted. |
| Li and Chen, 2015, China, UMIC (83) | Title NR. Questionnaire compromised both open- and closed-ended items. Administration format NR. | No details on questionnaire development were reported. | Total number of items and number of items related to perspectives NR.  Percentage provided when reporting results related to parents’ perspectives, but no scoring details were provided. | NR. |
| Liibaan et al, 2023, Scotland, HIC (84) | Title NR. Questionnaire compromised closed-ended items. Administration format NR. | Questionnaire was developed specifically for this study. | Total number of items NR.  Items related to perspectives NR.  Scoring details NR. | NR. |
| Little, 2019, UK, HIC (85) | Title NR. Questionnaire compromised both open- and closed-ended items. Questionnaire was delivered and fill-out as an online survey. | No details on questionnaire development were reported. | Total number of items and number of items related to perspectives NR.  No details on item scoring were reported. | NR. |
| Luo et al, 2023, Taiwan, HIC (86) | Title of questionnaire regarding parents’ perspectives was "Parental attitudes toward young children’s ICT use scale". Questionnaire compromised closed-ended items. Questionnaire were filled-out by parents in paper format at kindergartens and elementary schools. | Questionnaire was developed specifically for this study. | Total number of items NR.  Items related to perspectives: 23.  Items were scored using a 5-point Likert scale, where 1 = disagree completely and 5 = agree completely.  Two average scores were computed, one for “negative effects” and one for “positive effects”. | Content validity was evaluated by experts within the field.  Construct validity was evaluated using an exploratory factorial analysis for items related to perspectives. Based on this, a two-dimensional structure was derived.  Internal reliability score reported as 0.95, but which metric this was based on was not explicitly stated. |
| Mansor et al, 2021, Malaysia, UMIC (87) | Title NR. Questionnaire compromised closed-ended items. Administration format NR. | Questionnaire was adapted from previous studies and was translated into the Malay language by experts from a translation service. | Total number of items in the questionnaire: 71.  Number of items related to perspectives: 11.  Items related to parents’ attitudes towards screen time were rated on a 5-point Likert scale, where 1=Strongly disagree and 5=Strongly agree. The scores were categorized and parents who scored above 32 were rated as having a positive attitude.  Items related to parents’ perception of the influence of screen time on their child had the following three response options: “Negative influence”, “No influence” and “Positive influence”. Items were scored so that higher scores represented more positive perceptions. | The test-retest reliability of the original English version of the questionnaire was reported to be at a good to excellent level, with an intraclass correlation coefficient 0.64-0.98. |
| Matziou et al, 2021, Greece, HIC (88) | Questionnaire titled “Children’s Television-Viewing Habits Questionnaire (CTVQ)”. Questionnaire compromised closed-ended items. Parents completed the questionnaires at a scheduled appointment with the present of a researcher. | Questionnaire was adapted from a previous study and was translated into Greek following a 3-step process. | Total number of items in the questionnaire: 33  Number of items related to perspectives NR.  Percentage provided when reporting results related to parents’ perspectives, but no scoring details provided. | Internal validity was evaluated using Cronbach’s alpha, which was 0.71 for the complete questionnaire. |
| Mikelic Preradovic et al, 2016, Croatia, HIC (89) | Title NR. Questionnaire compromised closed-ended items. Questionnaires were distributed to parents during one week by kindergarten educators. Parents filled-out the questionnaire at home. | Questionnaire was developed specifically for this study by early childhood education experts, kindergarten principal and digital technology expert. | Total number of items NR.  Number of items related to perspectives: 10.  Items were rated on a 5-point Likert scale, where 1=Strongly disagree and 5=Strongly agree. Four items were inversely coded, so that higher scores meant a more positive attitude of the parents toward their children’s computer use. | NR. |
| Milford et al, 2022, Australia, HIC (90) | Title NR. Questionnaire compromised closed-ended items. Questionnaire was delivered and fill-out as an online survey. | Questionnaire was adapted from a previous study. | Number of items NR.  Items were rated on a 5-point Likert scale, where 1=Strongly agree and 5= strongly disagree. | NR. |
| Nabi and Krcmar, 2016, USA, HIC (91) | Questionnaire titled “Parent Play Beliefs Scale (PPBS)”. Questionnaire compromised closed-ended items. Parents recruited from the daycare centre completed paper versions of the survey, while others completed the survey online. | Questionnaire was adopted from a previous study. | Total number of items NR.  Number of items related to perspectives: 9.  Items were rated on a 7-point Likert scale. | Construct validity was evaluated using a factor analysis for the full set of items in the original version of the questionnaire related to parents’ perspectives on media, which revealed two dimensions: learning (12 items) and fun (6 items). |
| Natsiopoulou et al, 2013, Greece, HIC (92) | Title NR. Questionnaire compromised closed-ended items. Administration format NR. | Questionnaire was adopted from a previous study. | Total number of items NR.  Number of items related to perspectives: 7.  Items were rated on a 5-point Likert scale, where 1=totally disagree and 5=totally agree. | NR. |
| Nikken, 2019, Netherlands, HIC (93) | Title NR. Questionnaire compromised closed-ended items. Questionnaire was delivered and fill-out as an online survey. | Questionnaire was developed specifically for this study based on a literature review. | Total number of items was NR.  Number of items related to perspectives: 24.  Items were rated on a 5-point Likert scale, where 1=fully disagree and 5=fully agree. Responses were averaged and reported in terms of means and standard deviations. | Face validity was evaluated by conducting a pilot study with 8 families who had children between 18 months and 12 years.  Construct validity was evaluated using an exploratory factor analysis for the full set of 31 items in the original version of the questionnaire related to parents’ perspectives on media effects. The final items had a loading higher than 0.45. |
| Nikken and Schols, 2015, Netherlands, HIC (94) | Title NR. Questionnaire compromised closed-ended items. Questionnaire was delivered and fill-out as an online survey. | Questions were derived from previous studies and from opinions generally encountered in public debates about children and digital media. | Total number of items NR.  Number of items related to perspectives: NR.  Items were rated on a 5-point Likert scale, where 1=fully disagree and 5=fully agree. Responses were averaged to generate a “positive media effects” score and a “media function as a pacifier” score, and reported in terms of means and standard deviations. | Internal validity was evaluated using Cronbach’s alpha, which ranged between 0.75-0.89 for items related to perspectives. |
| Njoroge et al, 2013, USA, HIC (95) | Title NR. Questionnaire compromised closed-ended items. Parents were asked to complete the survey during a home visit by study staff. | No details on questionnaire development were reported. | Total number of items NR.  Number of items related to perspectives: 2.  Items were rated using a 5-point Likert scale, where 0= strongly disagree to 4 = strongly agree. Responses were dichotomized to agree, neutral or disagree. | NR. |
| Nwankwo et al, 2019, UK, HIC (96) | Title NR. Questionnaire compromised closed-ended items. Questionnaire was delivered and fill-out as an online survey. | Questionnaire was developed specifically for this study. | Number of items NR.  Results from questionnaire reported in terms of percentage, but no details on item scoring were provided. | NR. |
| O’Connor and Fotakopoulou, 2016, UK, HIC (97) | Title NR. Questionnaire compromised both open- and closed-ended items. Questionnaire was delivered and fill-out as an online survey. | NR. | Number of items NR.  Results from closed-ended items were reported in terms of percentage, but no details on item scoring were provided.  Open-endedended questions were coded and presented in relation to the categories that arose from recurrent themes in the responses. | NR. |
| Ophir et al, 2023, Isreal, HIC (98) | Title NR. Questionnaire compromised closed-ended items. Questionnaire was filled-out online using Qualtrics. | Questionnaire was developed specifically for this study. | Total number of items NR.  Items related to perspectives: 6.  Items were scored using a 5-point Likert scale, where 1=completely disagree and 5=fully agree.  Two average scores were computed, one for negative attitudes and one for positive attitudes. | Construct validity was evaluated suing a (unspecified) factor analysis for the 6 items related to perspectives in both Study 1 and Study 2. The analysis revealed a two-dimensional structure that together explained 70% (Study 1) and 65% (Study 2) of the original variance. Factor loadings ranging between 0.704-0.850 (Study 1) and 0.686-0.811 (Study 2) |
| Petegem et al, 2019, Belgium, HIC (99) | Title NR. Questionnaire compromised closed-end items. Administration format NR. | NR. | Total number of items in the questionnaire: 6.  Number of items related to perspectives: 6.  Items were rated on a 5-point Likert scale, where 1=completely untrue and 5=completely true.  Results reported in terms of means and standard deviation, but no details on item scoring were provided. | Construct validity was evaluated using a confirmatory factor analysis, which showed a root mean square error of approximation of 0.06 and a standardized root mean square residual of 0.02.    Internal validity was evaluated using Cronbach’s alpha, which was 0.86 for the complete questionnaire. |
| Raj et al, 2022, Malaysia, UMIC (100) | Title NR. Questionnaire compromised closed-ended items. Administration format NR. | Questionnaire was adopted from previous studies. | Total number of items NR.  Number of items related to parental attitude towards screen time: 8.  Items were rated on a 5-point Likert scale, where 1=strongly agree and 5=strongly disagree. Average score of above 3 was categorized as having a positive attitude, while those scoring below 3 were classified as having a negative attitude towards screen time.  Number of items related to parents’ perception regarding the influence of screen time on their child’s well-being: 11.  Items were classified into physical, cognitive and social wellbeing. They were rated on a 3-point scale with “positive influence” given a score of 3, “no influence” given a score of 2 and “negative influence” given a score of 1. A one-unit increase indicated a greater perception of positive influence of screen time on a child’s wellbeing. | Face validity was evaluated by conducting a pilot test among 50 parents who were not included in the main study.  Internal consistencies of items related to parental attitude towards screen time and parents’ perception regarding the influence of screen time on their child’s well-being were 0.84 and 0.81, respectively. However, the measurement to evaluate internal consistency was not specified. |
| Raj et al, 2023, Malaysia, UMIC (101) | Title NR. Questionnaire compromised closed-ended items. Questionnaire was filled-out online via Google Form that was distributed to the parents via WhatsApp at baseline, immediately after the intervention and 3 months after the intervention. | Questionnaire was developed based on a previous study (140). | Total number of items NR.  Items related to perspectives: 11.  Items were scored using a 5-point Likert scale, where 1=strongly disagree and 5=strongly agree.  The items were classified into physical, cognitive, and social well-being with higher scores representing more positive perceptions of screen time on child’s well-being. | The internal reliability for each construct was 0.88, 0.90, and 0.72, respectively. The metric on which this was based was not reported. |
| Rajalakshmi et al, 2023, India, LMIC (102) | Title of questionnaire was "Digital Screen Exposure Questionnaire" (DSEQ). Questionnaire compromised closed-ended items. Administration format NR. | Questionnaire was developed in a previous study (120). | Total number of items: 86.  Items related to perspectives: NR.  Results reported as frequency count and percentage, but no details on scoring were provided. | NR. |
| Rosanda et al, 2022, Slovenia, HIC (103) | Title NR. Questionnaire compromised closed-end items. Questionnaire was delivered and fill-out as an online survey. | Questionnaire was developed specifically for this study. | Total number of items NR.  Number of items related to perspectives: 21.  Parents were asked to tick off which positive effects they believed digital technology had on their child’s development.  Frequency and percentage provided when reporting results related to parents’ perspectives, but no scoring details were provided. | NR. |
| Sada Garibay and Lapierre, 2024, Mexico, UMIC (104) | Title NR. Questionnaire comprised closed-ended questions. Administration format NR. | NR. | Total number of items NR.  Items related to perspectives: 1.  Items were scored using a 4-point Likert scale, where 1 = "this media does not represent any risk” and 4 = “this media can represent a significant risk. I consider it very important to take safety measures.” | NR. |
| Seršen et al, 2024, Slovenia, HIC (105) | Title NR. Questionnaire compromised open- and closed-ended items. Questionnaire was delivered and fill-out as an online survey. | Parts of the questionnaire were adopted from previous studies. Two additional items related to perspectives were developed specifically for this study. | Total number of items: 62.  Items related to perspectives: 2.  The two items related to perspectives were open questions about parents’ opinions on how children’s programmes positively and negatively impact the development and learning of their child. If answering “yes”, they need to name three corresponding aspects. | NR. |
| Solomon-Moore et al, 2017, UK, HIC (106) | Title NR. Questionnaire compromised closed-end items. Administration format NR. | NR. | Total number of items was not reported.  Number of items related to perspectives: 2.  Items related to perspectives were rated using four 5-point Likert scales with anchor points: ‘beneficial ≥ harmful,’ ‘healthy ≥ unhealthy,’ ‘useful ≥ of no use,’ and ‘of no concern ≥ of concern’.  Results related to perspectives reported as means and standard deviation, but no details on item scoring were provided. | NR. |
| Stuckelman et al, 2023, USA, HIC (107) | Title NR. Not reported if items were open or closed-ended. Questionnaire was delivered and fill-out as an online survey. | Questionnaire was adopted from published measures (141-143). | Total number of items: 32.  Items related to perspectives: 12.  Scoring details NR. | Internal validity was evaluated using Cronbach’s alpha, which was 0.95 for the items related to perspectives. |
| Suresh and Tiwari, 2023, India, LMIC (108) | Questionnaire titled “Parental survey on media technology and screen time (MeTS) usage in children”. Questionnaire compromised closed-end items. Parents were provided with the survey link (Google form) through email and online social applications to fill the survey. | Questionnaire was developed by including selected questions from previous studies and was adapted to suit the Indian socio-cultural context. | Total number of items in the questionnaire: 73.  Number of items related to perspectives: 28.  Items were rated on a 5-point Likert scale, where 1=strongly agree and 5=strongly disagree.  Open-ended questions were analysed using a six-phase thematic analysis. | Content validity was evaluated using the Content Validity Index based on inputs from six experts within the field. Item and scale levels ranged between 0.83 to 1.00.  Internal validity was evaluated using Cronbach’s alpha, which ranged between 0.7-0.8, for the complete questionnaire. |
| Tanusha et al, 2023, Malaysia, UMIC (109) | Title NR. Questionnaire comprised closed-ended questions. An envelope containing the questionnaire was given to the selected children and fill-out in paper format by the parents. The questionnaire was returned to class teachers within a week and then collected by the researcher. | Questionnaire was developed specifically for this study based on two previous studies (59, 144). The questionnaire was developed in English and then forward and backward translated into the local language by two linguists. | Total number of items NR.  Items related to perspectives: 24  Items were scored using a 5-point Likert scale, where 1 = strongly agree and 5 = strongly disagree.  ‘Agree’ and ‘strongly agree’ were analysed together as agreement with the statement, while ‘disagree’ and ‘strongly disagree’ were analysed together as disagreement with the statement.  The overall perspective was assessed by a single question, followed by three answer options of ‘yes’, ‘no’ or ‘unsure’. | Face validity was evaluated by conducting a pilot test among 30 parents.  The internal consistency for items related to perceived risks and benefits was 0.974 and 0.713, respectively. No details provided on the metric used. |
| Tay et al, 2021, Singapore, HIC (110) | Questionnaire titled “Surveillance of digital media habits in early childhood questionnaire (SMALLQ)”. Questionnaire compromised closed-ended items. Questionnaire was delivered and fill-out as an online survey. | Questionnaire was developed by the research team in a previous study (135). | Total number of items in the questionnaire: 22  Number of items related to perspectives: 12.  Items were rated on a 5-point Likert scale, where 1=not important at all/ not concerned and 5=very important/ seriously concerned. | Content validity was established in a previous study with assistance from a panel of experts. |
| Vaala and Hornik, 2014, USA, HIC (111) | Title NR. Questionnaire compromised closed-end items. Questionnaire was delivered and fill-out as an online survey. | Questionnaire was developed specifically for this study based on results of an elicitation interview study with a separate sample of mothers. | Total number of items NR.  Number of items related to perspectives: 3.  Items related to perspectives were rated on a 7-point scale. The responses were averaged, with higher values indicating more positive perspectives. | Internal validity was evaluated using Cronbach’s alpha, which was 0.94 for the items related to perspectives. |
| Vaiopoulou at al, 2021, Greece, HIC (112) | Questionnaire titled “Perceptions about Educational Apps Use-parents (PEAU-p)”. Questionnaire compromised closed-end items. The questionnaire was uploaded on a web-based form via Google Forms, and parents completed it anonymously. | Questionnaire was developed specifically for this study based on literature review and using valid items used previously, which were adopted and refined. | Total number of items in the questionnaire: 27.  Number of items related to perspectives: 27.  Items were measured on a 7-point Likert scale. No further details on item scoring were provided. | Construct validity was evaluated using a principal component factor analysis and a confirmatory factor analysis.  The results showed factor loading values ranging between 0.54-0.89, a root mean square error of approximation of 0.027 (90% CI = [0.000; 0.040]), and a standardized root mean square residual of 0.07.  Internal validity was evaluated using Cronbach’s alpha, which ranged between 0.78-0.89.  . |
| Vittrup et al, 2016, USA, HIC (113) | A survey titled “*Attitudes, Perceptions, and Decisions Related to Technology Use with Young Children”*. Not reported if items were open or closed-ended.  Parents could fill-out paper or online versions of the questionnaire. Those who filled-out the paper version returned their survey in a sealable envelope to a collection box placed at the child-care centres. A research assistant collected the envelopes every week. | Questionnaire was developed specifically for this study. | Total number of items in questionnaire: 70.  Total number of items related to perspectives: 9.  Items were scored using a 7-point Likert scale.  The possible range of scores was 9–45. | NR. |
| Vincent et al, 2021, France, HIC (115) | Title NR. Questionnaire compromised both open- and closed-ended items. The questionnaire was filled-out in paper format. Completed questionnaires were returned in a locked mailbox accessible in the emergency room from which the parents were recruited. | NR. | Total number of items NR.  Total number of items related to perspectives: 13.  Frequency and percentage provided when reporting results related to parents’ perspectives, but no scoring details were provided. | NR. |
| Wang et al, 2024, China, UMIC (114) | Title NR. Questionnaire comprised closed-ended questions. The questionnaire was sent out by the managers to parents’ online accounts. | Questionnaire was adapted from previous studies (148, 149). | Total number of items NR.  Items related to perspectives: 2.  Items were scored using a 5-point Likert scale, where 1 = totally disagree, and 5 = totally agree. | NR. |

*NR = not reported.* **^A^***Covering evaluations of face validity, content validity, construct validity, and/or internal validity either for the overall questionnaire or, if specified, for the items related to measuring perspectives.*
