# Supplementary material for: Questionnaires Used to Explore the Perspectives of Parents and Health Professionals on Young Children’s Use of Technology: Systematic Review
Source: JMIR Pediatr Parent. 2026 Jun 11;9:e84712. doi: 10.2196/84712 (PMC13256481; doi:10.2196/84712)
Supplement: Multimedia Appendix 4 [file pediatrics-v9-e84712-s004.docx]

Table S1 presents reported questionnaire items used for measuring parents’ perspectives on children’s technology use.

**Table S1.** *Questionnaire items related to perspectives from each study.*

| **Author, year, country** | **Items related to perspectives** |
| --- | --- |
| Akyol, 2022, Turkey (31) | NR. |
| Aladé and Donohue, 2022, USA (32) | Parents were asked to rate their level of agreement (on a 4-point Likert scale) with the following statements regarding mobile device use at home:   - Having a mobile device in the home makes my job as a parent easier. - My child learns a lot when they use the mobile device. - My child enjoys using mobile devices. - I enjoy using the mobile device with my child. - I am concerned that my child uses the mobile device too much.   Parents were asked to rate their level of agreement (on a 4-point Likert scale) with the following statements regarding mobile device use at school:   - It is important for children to learn how to use technology in school. - Technology use in school distracts from learning. - There can be a healthy balance between traditional teaching methods and teaching with technology. - Tablets can be useful learning tool at school. - Tablet use takes away from other more important learning. |
| Al-Balushi and Al-Shihi, 2016, Oman (33) | No information provided on how parents responded to the following items:   - It is normal that my children own mobile devices. - I feel safe when my children use mobile devices when I am not with them. - It is normal that my children have access to social networks via mobile device. - It is normal that my children play online games via mobile devices. - It is good for a specific age of children to use and own mobile devices. - It is okay that parents do not allow their children to use mobile devices. - It is okay to allow the Wi-Fi network being connected to my children’s mobile device all the time. - I feel safe when my children share personal photos via social networks. - I feel safe when my children contact strangers. - I talk with my children about mobile devices’ risk. |
| Alkalash et al, 2023, Saudi Arabia (34) | Parents were asked to rate their level of agreement (on a 3-point Likert scale) with the following statements regarding exposure to screens for their children under the age of 6:   - Do you worry about the use of smartphones/tablets by your children? - Do you think it is your duty to limit the content that your son watches? - Do you find difficulty to control your child's use of smartphones/tablets or television, especially when there are many of these devices at home? - Do you face difficulty to monitor your child’s use of smartphones/tablets or television due to your busyness with work? - Have you got annoyed by your child eating a lot while busy using smartphones/tablets or watching TV? - Do you feel that the presence of smartphones/tablets at home reduces the time the family spends communicating or playing with its children? |
| AlSamhori et al, 2023, Jordan (35) | Parents were asked to tick off their opinion (yes/no) towards the following statements:   - Does the usage of technology improve your child mental and psychological growth? - Does the usage of technology impact negatively your child mental and psychologic growth? |
| Amzalag, 2021, Isreal (36) | Only example of items provided.  Parents were asked to rate their level of agreement with nine items regarding their attitudes towards digital games (e.g. “The digital game is a waste of time”); seven items regarding their attitudes towards digital learning games (e.g. “Digital learning games encourage the learner to show responsibility and active involvement in the learning process”); and eight items regarding their attitudes towards digital learning games as an alternative for traditional homework (e.g. “Digital learning games, given as homework, allow learners to control the learning topics, thanks to immediate feedback”). |
| Arippin et al, 2023, Brunei Darussalam (37) | No information provided on how parents responded to the following items:  Items related to “knowledge”:   - Increase in children’s screen time is likely to decrease their effort in physical activity. - Children’s sleep pattern and quality can be disrupted by increase in their screen time. - Increased children’s screen time may increase risk of the children being overweight/obesity - Increased children screen time is more likely to increase consumption of soft drinks and snacks. - Children that spend more screen time are at risk of emotional, mental and behavioural problems. - Uncontrolled children’s screen time can lead to addiction to the devices.   Items related to “attitude”:   - I have the responsibility to control our child(ren)’s screen time by paying close attention on the appropriateness of the screen time activities. - I should not be concerned about our child(ren)’s screen time and they can engage for as long as they want. - It is challenging to manage our child(ren)’s screen time when there is a lot of screen-based devices available in out household. - It is difficult to constantly supervise our child(ren)’s screen time activity when there is increase household and/or work demand. - I would not consider my child(ren)’s level of screen time to be a serious matter if he/she/they is/are active, healthy and well-behaved. - I am aware that our child(ren) engagement with screen time is influenced by our use of screen-based devices and/or by others (e.g. siblings and/or friends). - I observed that our child(ren)’s use of screen device interferes with our family quality time. - I am concerned about our child(ren)’s unhealthy food intake when engaging in screen-based activity.   Items related to “practice”:   - I encourage my child(ren) to play with toys or talk face-to-face rather than spending time every waking hour, using mobile phone, watching TV/video, and on laptop. - I ensure that I take away my child(ren) screen-based devices at home when they play or have social activities. - I try to limit or not use screen-based devices whenever I am with my child(ren). - I give screen-based devices to my child(ren) to keep them temporarily occupied and be quiet especially in time when I am busy and when he/she get fussy or moody. - I usually stop my child(ren)’s screen time at least an hour before bedtime to get him/her to fall asleep. - I offer screen-time to my child(ren) as a reward for good behaviour and removing it as a punishment for bad behaviour. - I do not allow my child(ren) to have any kinds of screen-based devices during family time (e.g. meal time) or in his/her/their bedroom. |
| Asplund et al, 2015, USA (38) | Only example of items provided.  Parents were asked to rate their level of agreement (on a 5-point Likert scale) on whether they perceived TV as valuable or useful (e.g. ‘‘young children who never watch TV miss a lot that is of value’’; ‘‘TV is a useful way of keeping the children amused’’) and they were restrictive about child TV viewing (e.g. ‘‘I restrict how much time my child spends watching TV’’; ‘‘I have to be sure my child does not watch too much TV’’; ‘‘I will switch off the TV if I think my child is watching too much’’). |
| Balaban and Bayindir, 2019, Turkey (39) | Only examples of items provided.  Parents were asked to indicate frequency (on a 5-point Likert scale) on three items related to an ‘Entertainment’ dimension (e.g. “My child is entertained when s/he is engaged in digital games”); eight items related to a ‘Learning’ dimension (e.g. “Digital games increase my child’s interest in mathematics”); six items related to a ‘Physical’ dimension (e.g. “My child sits in a hunchback position while playing a digital game”); five items related to a ‘Social’ dimension (e.g. “While playing digital games, my child’s communication with us decreases”); and three items related to a ‘Emotional’ dimension (e.g. “Playing digital games makes it difficult for my child to express his/her feelings (S/he does not want to use expressions like “happy”. “sad” etc)). |
| Bansal et al, 2023, India (40) | Same items as reported in Arippin et al. (2023). |
| Barmomanesh et al, 2017, New Zealand (41) | NR. |
| Beyens and Eggermont, 2014, Belgium (42) | Only examples of items provided.  Parental attitude toward television was assessed by asking parents to rate their level of agreement (on a 5-point Likert scale) toward seven positive outcomes of television among young children (e.g. “Children who are heavy TV viewers learn a lot of new words”). |
| Bleakley et al, 2013, USA (43) | Parents were asked to rate their level of agreement (information on scale details not provided) as to whether limiting/ continuing to limit their child’s television viewing to 2 h or less every day would be:   - complicated/ simple - bad/ good - foolish/ wise - unpleasant/ pleasant - easy/ difficult - harmful/ beneficial |
| Boonmun et al, 2023, Thailand (44) | Parents were asked to rate their level of agreement (on a 5-point Likert scale) for a range of questions regarding children’s screen time reduction, but only one example item was provided: “Parents are important individuals for limiting children’s screen time.” |
| Bourha et al, 2024, Greece (45) | Parents were asked the following question: “Have you noticed any improvements/progress/enrichment of knowledge in your child that you think is related to playing with this toy?”. By answering “yes”, the following question was asked: “If yes, in which developmental area?” with the following response options:   - Speech/vocabulary - Gross motor activity or in a coordinated manner - Fine manipulation - Social-emotional development - Mathematical concepts - Creativity - Communication - Science concepts - Other   Parents were asked to indicate the significance of their child’s engagement with technology, but no information was provided on how this was measured. |
| Brauchli et al, 2023, Switzerland (46) | Parents were asked to rate their level of agreement (on a 6-point Likert scale) with the following statements regarding their children’s screen media use:   - I find it good when [child’s name] uses screen media. - It is important for children to learn how to use screen media responsibly as early as possible. - Children must learn to use screen media as early as possible. |
| Brown et al, 2023, USA (47) | Parents were asked to tick off up to 3 of the most important options for the following items:  What do you think are the most negative effects of digital media use in children?   - Sleep problems/ Getting out of shape or obesity - Addiction to gaming or screens - Loss of social skills or being impolite - Aches and body pains - Attention problems - bullying/ Depression - Vision problems - Delay in learning language skills - Aggression - Trouble calming himself/herself when frustrated or upset - Low self-esteem - Exposure to violent or inappropriate content - None - Other   What do you think are the most positive effects of digital media use in children?   - Exposure to a wider vocabulary - Exposure to foreign languages - Relaxation or fun - Learning how to multi-task more easily - Increased creativity - Increase in school readiness - Increased hand-eye coordination or skill with hands - Increased communication with distant relatives or friends - Learning about other cultures, places, and ideas - None - Other |
| Cardy et al, 2023, Canada (48) | No information provided on how parents responded to the following item:   - Overall, how does technology impact your child’s quality of life?   Parents were asked to tick off the domain in which their child benefited from technology use:   - Social skills - Motor skills - Emotion regulation - Language and communication - Cognitive development - Leisure/ recreation - Transition - My child has not benefited from technology in any area - I don’t know |
| Carson et al, 2012, Canada (49) | Parents were asked to rate their level of agreement (on a 4-point Likert scale) as to whether their child’s screen time is:   - It is good for his/her brain - It is something my child finds very enjoyable - It gives me the opportunity to get things done on my own - It allows me to cope from a busy day at work and/or looking after multiple children - My child needs/wants time to relax - It is family time, bonding time, or quality time - It grabs hold of my child’s attention - It teaches my child to get along with others |
| Carson et al, 2013, Canada (50) | Parents were asked to rate their level of agreement (on a 4-point Likert scale) as to whether their child’s screen time is:   - It is a good learning tool - It is child enjoyment - It gives parent opportunity to get things done - It allows parent to cope with busy day/multiple children - It allows child to relax - It is family, bonding or quality time - It grabs hold of child’s attention - It teaches children to get along with others   Parents were asked to rate their level of agreement (on a 4-point Likert scale) with the following statements regarding their perceived barriers towards reducing their child’s screen time:   - Pressure from society to purchase media equipment - Neighbourhood is unsafe - Poor weather limits time outside - Parent needs coping-tool for busy day/multiple children - Parent needs time to do household chores - Child really enjoys screen time - Child does not engage in too much screen time |
| Chattha et al, 2021, Pakistan (51) | NR. |
| Chia et al, 2022, Singapore (52) | Parents were asked to rate the level of importance (on a 5-point Likert scale) of the following statements regarding their child’s digital media use:   - Improve child’s knowledge and skills - Keep child entertained - Communication - Keep child occupied - Distract or divert child’s attention - Put child to sleep   Parents were asked to rate the level of concern (on a 5-point Likert scale) about the following statements regarding their child’s digital media use:   - Poor sleep - Poor eyesight - Lack of physical exercise and activity - Exposure to inappropriate content - Addiction - Lack of parent-child interaction |
| Chen and Tu, 2018, Taiwan (53) | Parents were asked to rate their level of agreement (on a 5-point Likert scale) with the following items regarding the usefulness of the internet for preschool children:   - Internet-based instruction provides more knowledge for young children. - Internet-based instruction develops learning autonomy for young children. - Internet-based instruction provides more learning resources for young children. - Internet-based instruction develops peer relationships among young children. |
| Cingel and Krcmar, 2013, USA (54) | Only examples of items provided.  For perceived positive effects of media, parents were asked to rate their level of agreement (on a 7-point Likert scale) as to whether a variety of media were beneficial to their child’s cognitive, social and physical development (e.g. “I believe education television and videos are helpful to my child’s cognitive development”).  For perceived negative effects of media, parents were asked to rate their level of agreement (on a 7-point Likert scale) as to how worried they were that electronic media were harming their children in terms of their cognitive, social and physical development (e.g. “I worry that electronic media will have a negative effect on my child’s cognitive development”). |
| Covolo et al, 2021, Italy (55) | Parents were asked if they believed the use of smartphones and/or tablets by a preschooler (0-5 years old) posed a risk to his/her health. If yes, they were asked to rate how harmful they thought it was on a scale of 1 to 7.  Parents were asked to rate the level of risk (on a 7-point Likert scale) they believed the use of smartphone and/or tablet would cause a preschooler to:   - Have sleep disorders. - Become obese. - Become epileptic. - Have eye irritation. - Become celiac. - Confuse virtual reality with the real world.   Parents were asked to rate the level of benefit (on a 7-point Likert scale) they believed the use of smartphone and/or tablet would be to a preschooler in terms of:   - Learning new words (for children aged under 2 years old). - Learning new words (for children aged 3-5 years old). - Developing cognitive or creative skills. - Preventing Attention Deficit and Hyperactivity Disorder. - Communicating with distant relatives. - Approaching technology. |
| Dardanou et al, 2020, Norway, Portugal and Japan (56) | Parents were asked to tick off all they believed applied for the following item:  What advantages do you think there are to your child/children using touchscreens?   - They learn new skills. - They learn new knowledge. - It keeps them occupied. - It entertains them. - It allows them to be creative. - It will help them when they go to school. - Other   Further, parents were asked if they had concerns about their child/children using touchscreens and, if yes, to elaborate on what their concerns were related to either children aged 0-2 years old and/or children aged 3 years and over. |
| Dong et al, 2022, China (57) | Parents were asked to rate their level of agreement (on a 6-point Likert scale) with the following statements:   - The use of digital devices is beneficial for early learning and development - Digital devices are suitable for young children to use - The use of digital devices is beneficial for children’s social interaction - The use of digital devices is harmful to the health of young children - The use of digital devices supports children’s future learning or development   Furthermore, parents were asked the following open-ended item: “How do you view young children’s digital use at an early age? Why?” |
| Eales et al, 2021, USA (58) | Only examples of items provided.  Parents were asked to indicate how helpful/hurtful (on a 5-point Likert scale) children’s media use was across six different domains of their child's life: social skills, learning, ability to focus, behaviour, physical activity and creativity (e.g. “Overall, do you think your child’s media use helps, hurts or makes no difference to his/her [domain]”). |
| Ebbeck et al, 2016, Singapore (59) | NR. |
| Fan et al, 2022, China (60) | Parents were asked to tick off what they believed about young children using short-video apps, with the following options:   - It is a waste of time - It enables digital play and releases children’s pressure - It may result in vision problem or other health issues - It enables children to learn academic knowledge and skills - Children may be addicted to digital media - It can broaden children’s vision and understanding of the world - Children may be influenced by low-quality/value contents - It satisfies children’s social needs - Other |
| Farima et al, 2023, Moldova (61) | NR. |
| Garcia-Conde et al, 2020, Spain (62) | Parents were asked to rate their level of agreement (on a 5-point Likert scale) with the following statements:   - It is harmful that my child watches TV/screen. - Watching TV/screen is healthy entertainment for my child. |
| Gjelaj et al, 2020, Kosovo (63) | NR. |
| González-Sanmamed et al, 2023, Spain (64) | NR. |
| Grané et al, 2023, Spain (65) | NR. |
| Griffith et al, 2023, USA (66) | NR. |
| Halpin et al, 2021, Australia (67) | Only examples of items provided.  Parents were asked to indicate the effect (on a 11-point Likert scale) of their child’s current screen use on their child’s functioning (8 items, e.g. “My child’s overall behaviour”); their child’s development (7 items; e.g. “My child’s early learning [e.g. colours, numbers, letters]”); parent-child interaction (5 items; e.g. “How patient I am with my child”); and parent self-care (3 items; e.g. “How easily I can take a break while caring for my child”). |
| Hamilton et al, 2016, Australia (68) | No information provided on how parents responded to the following items regarding how likely they were to limit their child’s screen time to less than one hour per day in the next week to:   - Promote my child’s creativity - Improve my child’s mental well-being - Improve my child’s social skills - Improve my child’s behaviour - Promote family interactions - Promote healthy habits in my child - Increase parent-child confrontations - Increase whining behaviour in my child - Interfere with my other commitments - Increase parent distress |
| Hatzigianni et al, 2014, Australia (69) | NR. |
| Howie et al, 2020, Australia & USA (70) | Parents were asked to rate their level of agreement (on a 5-point Likert scale) with the following statements:   - Using mobile technology will benefit my child overall. - Using mobile technology will be good for my child’s education. - Using mobile technology in child care will be good for my child’s education. - Using mobile technology will improve my child’s social skills. - Using mobile technology is a risk for my child overall. - Using mobile technology will be bad for my child’s education. - Using mobile technology will harm my child’s social skills. - Using mobile technology will make my child at risk to online predators. - Using mobile technology will cause my child discomfort and pain (i.e. back/neck pain). - Using mobile technology will increase the amount of time my child spends sitting. - Using mobile technology will reduce my child’s physical activity. |
| Hutton et al, 2018, USA (71) | Parents were asked to rate the level of importance (on a 5-point Likert scale) of TV viewing for the healthy development of babies <3 months old. |
| Ihmeideh and Alkhawaldeh, 2017, Jordan (72) | Preschool teachers and parents were asked their level of agreement (on a 5-point Likert scale) to the following statements regarding technology and digital media use by children:  Physical/health aspect   - Enhancing children’s movement skills and physical activity. - Raising children’s health awareness (disease prevention, health food, etc.). - Increasing children’s knowledge about safety procedures in their life.   Intellectual aspect   - Developing children’s thinking skills. - Helping children develop creativity. - Improving children’s language and literacy. - Developing children’s problem-solving skills. - Developing children’s numeracy skills.   Emotional aspect   - Helping children in controlling and managing their emotions. - Protecting children from fear, shyness, and anxiety. - Helping children sympathize with others and identify with community members.   Social aspect   - Increasing children’s awareness of their society and its problems. - Helping children cultivate feelings of belonging and citizenship. - Helping children communicate and interact with others. - Helping children know both, their social rights and duties.   Moral aspect   - Helping children cultivate positive morals and values. - Helping children exercise the highest ethics. - Helping children distinguish between good and evil, right and wrong. - Helping children maintain the prevailing good practices and traditions.   Religious aspect   - Developing children's positive attitudes toward their religious teachings. - Helping children learn about certain religious duties. - Helping children acquire religiously inspired social etiquette.   Aesthetic aspect   - Increasing children's ability to understand and appreciate the Arts (acting, singing, theatre). - Helping children learn tidiness, order and consistency. - Helping children experience things and appreciate their beauty. - Helping discover and develop children’s preferences, interests and skills. |
| Ilgar and Karakurt, 2018, Turkey (73) | Mothers were asked to tick off (yes/no) if they agreed to the following statements regarding computer use among preschool children:   - Contribute to perception, attention, and memory development in children. - Contribute to the development of hand-eye coordination in children. - Contribute to the development of creativity in children. - Have a negative impact on children’s socialisation. - Contribute to children’s self-production. - Meet children’s need for alone time. - Support children’s exploratory learning. - Enhance children's better and more frequent technology use. - Lead children not to spare time for actual games. - Restrain children’s family time. - Lead children to use more violence. - Help children discharge their aggressive impulses. - Lead children to become detached from the real world because of the cyber world. - Lead children to obtain everything fastly and become idle. - Have reflections on children's behaviour. - Lead children to mimic in real life what they were exposed to. - Results in dependency if use is not regulated. - Have a negative impact on children’s behaviour. - Lead children to become similar to the game characters. - Are used as a solution when families are tired and cannot spare time for the children. - Children prefer to spend time with their families instead of playing computer games. |
| Istenic et al, 2023, Slovenia (74) | Parents were asked to tick off their opinion (yes/no) regarding to the contribution of traditional and digital games to their child’s development.   - Sensory development (e.g., strengthens visual, auditory, and tactile skills). - Motor development (e.g., encourages the child to move, sit, climb, stand up, and walk; promotes hand-eye coordination). - Cognitive development (e.g., supports the development of speech, sounds, first words, and language skills; contributes to learning the alphabet, numbers, names of objects, etc). - Emotional development (e.g., develops the ability to manage one’s own emotions; empathy). - Listening and observation. - Visual and spatial orientation. |
| Istenic et al, 2023b, Slovenia (75) | No information provided on how parents responded to the following statements regarding digital play’s contribution to the learning and development of their child’s play:   - Media exposure at a young age (0-3 years) is important for early brain development. - A child with not fall behind other children academically if his or her use of technology tools is restricted in the early years (0-3 years). - Children under the age of 2 years should have no TV screen time. - Children between 2 and 5 should limit their screen technology to 1 hour a day. - Children between 2 and 5 should only use screen technology with their parents present. - The use of a computer can promote long-term physical, emotional, or intellectual developmental damage. - Introducing technological tools at a young age prepares children better for tomorrow’s work force. - Children today naturally understand how to use computers and related technology at an early age. - In early childhood, children can use technology as a learning tool. - Young children develop better without technology. - My child should first learn to interact with the physical world; he has a lifetime to interact with the virtual world. - I am concerned about how technology can affect a young child’s brain development and learning. - I want the child to enjoy their childhood and not become addicted to technology. - If my child does not master technology, he will be excluded from peer groups. - Due to the intensive use of digital technology in the family, parents are reading less and less to their children. - Technological toys please children more than traditional. - Preschoolers better develop without technology. - With the help of technological toys, parents easily guide their child’s play. - Technological toys are better than traditional ones. - Some technological toys enhance a child to develop early literacy. - Technological toys enhance a child to be passive. - Children under age three should not use technological toys. - During playtime, the most important communication is between a child and the engaged person. - Adults are less patiently following a child initiative with a play with a technological toy than with a traditional. - More important is communication between a child and person present than a toy itself. - Technological toys enable a child to learn how to manipulate technology. - Adult leads and teaches a child how to play with technological toy. - During the play, a child intensively communicates with the person/s present. |
| Jain et al, 2023, India (76) | Parents were asked to tick off if they agreed to the following items:   - Has your child ever complained of headache?   - If yes: Do you think the headache can be because of using screen (any screen TV/Mobile/Tablet/Laptop/computer) - Has your child ever complained of eye pain?   - If yes: Do you think eye pain can be because of using screen (any screen TV/Mobile/Tablet/laptop/computer) - Has your child every complained of itching in eyes?   - If yes: Do you think the itching in eyes can be because of using screen (any screen TV/Mobile/Tablet/Laptop/computer) |
| Jin, 2013, Korea (77) | Parents were asked to rate their level of agreement (on a 5-point Likert scale) with the following statement regarding their children’s internet use:   - I am always concerned that my children's Internet use will expose them to harmful information. - I always pay attention to my children’s Internet surfing and use. - It is very important to me to know what kinds of services my children access through the Internet. |
| Joginder Singh et al, 2021, Malaysia (78) | Parents were asked to rate their level of agreement (on a 5-point Likert scale) to the following statement regarding their children’s language skills: “My child has learnt new words from screen viewing.” |
| Konok et al, 2020, Hungary (79) | Parents were asked the following statement regarding their perspective toward mobile touch screen device use:   - It is better for a child to start mobile touch screen device use “as late as possible” or “as early as possible”, or with neither.   Parents were asked to tick off their perspective (yes/no) about the following statements regarding the potential harm of mobile touch screen device use:   - It causes behaviour problems/ disturbed behaviour. - It causes neurological problems/ attention deficit/ intellectual deficit. - It is harmful for development/ it doesn't help development. - It induces aggression/ irritation/ tension. - It causes depression/ bad mood. - It overstimulates the nervous system. - It deteriorates phantasy/ imagination/ creativity. - It takes time away from other things/the child doesn't play with other toys/games. - The absence of physical activity/ children are less physically active. - The child can lose her/his interest in other things/ they are more difficult to occupy with other things. - It ruins social relationships/ it causes problems in forming relationships/ they become 'antisocial'/ they become introverted/ it causes loneliness. - The child can come across inappropriate content. - Podophiles/ swindlers/ bad people can find her/him. - The excessive usage is harmful/ they can easily do it in excess. - It harms the eyes. - It can cause addiction/ it is hard to put it down.   Parents were asked to tick off their perspective (yes/no) about the following statements regarding potential benefits of mobile touch screen device use:   - It opens up the child’s world. - It helps maintain/ form relationships. - It increases openness/ the child will be more open to the world/ to new things. - The child can ask for help in emergency. - It offers leisure/ entertainment opportunity. - It improves fine motor skills. - It has improving effects/ it improves skills. - It improves logical thinking/ problem-solving/ intelligence. - She/he can learn through these things/ there are educational applications/ it helps in learning. - The child's knowledge grows/ getting information. - She/he can search for information about things in which she/he is interested/ satisfying curiosity. - It helps foreign language learning. - The child can learn informatics/ she/he can keep pace with technological development/ her/his digital skills improve. |
| Kostyrka-Allchorne et al, 2017, UK (80) | Parents were asked to rate how harmful (on a 5-point Likert scale) to children the following features of TV and films were regarding:   - Inappropriate language - Inappropriate behaviour - Violent content - Fast editing pace   Parents were asked to indicate the effect of media (on a 6-point Likert scale) on children’s development using the following items:   - The effects of popular media on children’s development are. - The effects of watching fast-paced action-filled programmes are. - The effects of watching educational programmes are. - The effects of watching programmes containing violence and threat are. |
| Lee et al, 2022, Korea (81) | Only examples of items provided.  Nine items were used to measure the positivity of attitudes (e.g. “I think watching media will positively influence my child’s behavioural development”).  Four items were used to measure the intellectual dimension of the negativity of attitudes (e.g. “I think watching media will hurt my child’s creativity”).  Four items were used to measure the social dimension of the negativity of attitudes (e.g. “I believe watching media will negatively affect my child’s play with friends”). |
| Lepicnik et al, 2013, Slovenia (82) | No information was provided on how parents responded to the following items regarding competences they thought the usage of information-communication technology mostly developed among children:   - Motor. - Learning/language. - Self-expression. - Social. - Cultural competences. |
| Li and Chen, 2015, China (83) | NR. |
| Liibaan et al, 2023, Scotland (84) | NR. |
| Little, 2019, UK (85) | NR. |
| Luo et al, 2023, Taiwan (86) | Parents were asked to rate their level of agreement (on a 5-point Likert scale) with the following statements regarding young children’s information communication technology use:   - Help children think flexibly - Provide a variety of learning content - Provide diverse stimuli for learning - Training the ability to react - Contribute to the development of cognitive skills (the promotion of thinking skills and knowledge) - Contribute to language learning - Training hand-eye coordination - Contribute to more familiarity with learning content - Help children to understand the learning content - Have a sense of accomplishment in learning - Contribute to active learning - Help to build self-confidence in learning - Rich audio and video help to improve interest in learning - Easily lead to phenomenon of technology-use addition - Develop the habit of overreliance on audio-visual learning - Affect the development of the optic nerve - Reduced interests in exploring the external environment - Reduce motivation and interest in reading - Reduce creativity - Hinder the development of the ability to live independently - Affect the development of thinking - Hinder the development of relationships - Establish improper values |
| Mansor et al, 2021, Malaysia (87) | Parents were asked to rate their level of agreement (on a 5-point Likert scale) with the following items regarding screen time:   - It is good for the development of his/her brain (such as learning aids). - It is something my child finds very enjoyable. - It gives me the opportunity to get things done on my own (such as house chores, cooking). - It helps me to handle a busy day at work and/or take care of children. - My child needs/wants time to relax. - It provides quality time together with family. - It grabs hold of my child’s attention. - It teaches my child to get along with others.   Parents were asked to indicate the influence of screen time (“Negative influence”, “No influence” or “Positive influence”) on their child’s:   - Physical wellbeing: Heart health/ Muscle and bone health/ Marinating a healthy weight/ Fundamental movement skills. - Cognitive wellbeing: Academic achievement/ Cognitive development/ Ability to concentrate/ language development. - Social wellbeing: School readiness/ Social competence/ Self-esteem. |
| Matziou et al, 2021, Greece (88) | NR. |
| Mikelic Preradovic et al, 2016, Croatia (89) | Parents were asked to rate their level of agreement (on a 5-point Likert scale) with the following items regarding children’s computer use:   - Children learn new and useful things on computer. - Children who regularly use computer are at risk of developing dependence. - When using computers, children gain valuable IT skills which will be useful for them in the future. - Computers can have only negative influence on children’s development. - Instead of playing with peers, children spend their time on the computer. - Children can better develop their skills playing computer games. - Children engage in sport less frequently due to the increased computer usage. - Excessive use of computers may separate children from their parents and friends. - Thanks to computers, children’s intellectual development is enhanced. - Children spend more quality time on the computer than watching TV. |
| Milford et al, 2022, Australia (90) | Only examples of items provided.  Parents were asked to rate their level of agreement (on a 5-point Likert scale) with a series of statements about the possible impact on their child in areas such as behaviour, attention, and academic performance. (e.g. “I feel that mobile media has a negative impact on my child’s behaviour,” “I feel that mobile media has a negative impact on my child’s academic performance,” and “I feel that mobile media has a negative impact on my child’s general attention.” |
| Nabi and Krcmar, 2016, USA (91) | Only examples of items provided.  Parents were asked to rate their level of agreement (on a 7-point Likert scale) with statements about learning (e.g. “Educational/entertainment electronic media can improve my child’s language and communication skills”) and fun (e.g. “Using educational/entertainment electronic media is a fun activity for my child”). |
| Natsiopoulou et al, 2013, Greece (92) | Parents were asked to rate their level of agreement (on a 5-point Likert scale) with the following items regarding the effects of children using computers:   - Offers joy and entertainment. - Promotes participation in learning. - Increases imagination and creativity. - Increase initiative. - Respects personal rhythms. - Limits free play. - Limits child’s reading. |
| Nikken, 2019, Netherlands (93) | Only examples of items provided.  Parents were asked to rate their level of agreement (on a 5-point Likert scale) regarding the effects of media on:   - learning (e.g. “My child can learn about the world via media”, “My child may know more about politics thanks to the Children’s News”), - social skills (e.g. “My child may improve his or her social skills by the use of media”, “My child can increase his or her peer group with good friends via social media”), - emotions (e.g. “My child can become calm and quiet from media use”, “My child can learn to improve his or her concentration by using media”), - behaviour (e.g. “My child may adopt rude language from the media”, “My child my get aggressive from seeing violence in the media”), and - health (e.g. “My child may get obese from media use”, “My child may develop sleeping problems because of media use”). |
| Nikken and Schols, 2015, Netherlands (94) | Only examples of items provided.  Parents were asked to rate their level of agreement (on a 5-point Likert scale) regarding:   - positive effects of media (e.g. “Screen media help my child to learn”, “Media can teach my child English”, “Electronic media will be good for my child’s school performances”), - using media as a pacifier (e.g. “Digital media give a moment of rest for my child”, “Media are a good pacifier for my child”, “Media make my child calm and peaceful”, “With media my child doesn’t have to be bored”), - negative effects of media (e.g. “Digital media let my child see or do inappropriate things”, “Media brings my child in contact with wrong people”, “I’d rather see my child play with other things than digital media”, “Digital media are not as good as normal toys for my child”), - and media as being too complicated (e.g. “Media are too complicated for my child”, “Media do not match with my child’s interests”). |
| Njoroge et al, 2013, USA (95) | Parents were asked to rate their level of agreement (on a 5-point Likert scale) with the following statements:   - Education TV programs can help preschoolers learn to recognize letters and numbers. - Educational TV programs can help preschoolers play netter with each other. |
| Nwankwo et al, 2019, UK (96) | NR. |
| O’Connor and Fotakopoulou, 2016, UK (97) | Parents were asked to tick off which of the following benefits they perceived for their 0-3-year-olds using touch screens:   - It entertains them. - They learn new skills. - It keeps them occupied. - They learn new knowledge. - Allows them to be creative. - Other advantage. |
| Ophir et al, 2023, Isreal (98) | Mothers were asked to state their level of agreement (on a 5-point Likert scale) with the following items:   - screen use can lead to cognitive problems, such as delays in brain development or impairments in attention capabilities - screen use can lead to emotional and social problems, such as impairments in emotion regulation and poor interpersonal capabilities - screen use may contribute to children’s cognitive functioning, for example, through online education - screen use may contribute to emotional and social functioning, for example through online communications with friends - it is important to limit screen time among children in the 1^st^ to 3^rd^ grades - it is important to limit screen time among children in the 4^th^ to 6^th^ grades |
| Petegem et al, 2019, Belgium (99) | Parents were asked to rate their level of agreement (on a 5-point Likert scale) with the following statement regarding digital gaming:   - Digital games are a waste of time. - Digital games are useless. - I believe that digital games pose a threat to children’s development. - Digital games are a meaningful activity. - I think it is good that children play digital games.   Playing digital games may be harmful to children. |
| Raj et al, 2022, Malaysia (100) | NR. |
| Raj et al, 2023, Malaysia (101) | NR. |
| Rajalakshmi et al, 2023, India (102) | Parents were asked to tick off their opinion (yes/no) towards the following statements (only some items provided in the result section):   - Increases his/her knowledge - Starts imitating what he/she watches - Might develop sleep problems - Might start eating unhealthy food - Might cause behaviour problems - Might impair eyesight - Has no negative effects - Has no positive effects |
| Rosanda et al, 2022, Slovenia (103) | Parents were asked to tick off which types of the following positive effects they believed digital toys (including digital without screen and screen-based) had for their children:   - Perceptual development - Motor development - Cognitive development - Emotional development - Listening, observation - Visual and spatial orientation - Promotes hand-eye-coordination - Stimulates the strengthening of reflexes - Encourages the development of social skills - Encourages the development of problem-solving skills - Encourages the development of basic mathematical skills - Encourages the development of basic reading skills - Encourages the development of basic language skills - Stimulates interest in science - Stimulates interest in arts and crafts - Stimulates interest in history - Allows child to search for information - Provides entertainment - Encourages the development of the skills needed to succeed in school - Keeps child busy - Stimulates the child’s interest in what is happening in his/her environment |
| Sada Garibay and Lapierre, 2024, Mexico (104) | Parents were asked to rate their level of perceived risk (on a 4-point Likert scale) using the following item:  Which of the following statements describes better your feelings in relation to the use of streaming platforms (Netflix, Claro video, APV, Blim, or other) by your child/children in elementary school? |
| Seršen et al, 2024, Slovenia (105) | Parents were asked to tick off their opinion regarding the following statements:   - Are you concerned about the possible negative effects of watching children’s programmes on your child’s development and learning?   - If answering “yes”, name three aspects of children’s programmes that you most fear could harm youryour child. - Do you believe that children’s programmes can have a positive effect on your child’s development and learning?   If answering “yes”, name three most positive aspects of watching children’s programmes. |
| Solomon-Moore et al, 2017, UK (106) | Parents were asked to indicate how ‘beneficial≥ harmful,’ ‘healthy≥ unhealthy,’ ‘useful≥ of no use,’ and ‘of no concern≥ of concern’ for the following statements:   - Children spending several hours per day watching television or playing video games. - Children spending several hours per day during leisure time using a computer or surfing the Internet. |
| Stuckelman et al, 2023, USA (107) | NR. |
| Suresh and Tiwari, 2023, India (108) | Parents were asked to rate their level of agreement (on a 5-point Likert scale) with the following statement regarding screen-based device use:   - I think my child is benefiting from screen based devices. - I am happy with the range of screen based devices my child has access to. - I believe screen based devices are making my child’s life better. - I think my child is skilled at using screen based devices. - I think screen based devices prevents my child from interacting with other people. - Screen based technology helps me to keep my child occupied when the family attends to other work. - I will introduce more screen based devices to my child he/she grows up. - I find it hard to choose the appropriate activities and games for my child in screen based devices. - I think it is necessary to give screen based devices exposure to children from an early age. - I think screen based devices is helping to improve my child’s attention and learning skills. - I think use of screen based devices increases the interactive opportunities for my child. - I prefer giving screen based devices over other toys for my child to play with. - I think it is necessary to encourage outdoor play activities along with screen based devices. - I believe screen based devices exposure has increased tantrums in my child. - I think screen based devices usage has negatively affected my child’s communication skills. - I think my child prefers screen based devices over face to face interactions. - I am concerned regarding child’s screen based devices usage. - Certain contents in screen based devices is inappropriate for young children. - My child spends too much time on screen based devices. - My child may get addicted to screen based devices. - My child losing interest in other activities and lacking real life functioning skills due to excessive screen based devices use. - Harmful effects of prolonged screen based devices usage on my child’s health. - Whether screen based devices might negatively affect my child’s learning, thinking, attention etc. - Whether increased used of screen based devices can negatively affect child’s creativity and imagination. - Increase use of screen based devices can result in social deficit, less social interaction and isolation of my child. - Excessive screen based devices can impair my child’s communication skills. - Excessive screen based devices can impair my child’s speech and language development. - My child’s screen based devices use might impact our cultural values. |
| Tanusha et al, 2023, Malaysia (109) | Parents were asked to tick off their opinion (yes/no/unsure) about the following statement:   - Do you think that your preschool child’s digital device use causes more benefit than harm?   Parents were asked to rate their level of agreement (on a 5-point Likert scale) with the following themes related to perceived risks of children using digital devices:  Physical   - Damages eyesight - Exposed to radiation - Inactive lifestyle   Intellectual   - Causes device addiction - Has undesirable contents - Causes over-dependence   Emotional   - Causes poor social-emotional development - Causes impatience - Encourages tantrums   Social   - Causes social isolation - Causes poor social skills - Causes poor communication skills   Parents were asked to rate their level of agreement (on a 5-point Likert scale) with the following themes related to perceived benefits of children using digital devices:  Physical   - Improves movements and coordination of hands and fingers - Improves sensation of vision, hearing and touch - Improves reflexes   Intellectual   - Improves academic achievement - Promotes creative and interactive learning - Enhances learning process   Emotional   - Appreciates music - Encourages independence - Reduces tantrums   Social   - Promotes technology awareness - Easily accessible and portable - Entertaining |
| Tay et al, 2021, Singapore (110) | Parents were asked to rate the importance (on a 5-point Likert scale) of digital media use regarding:   - To improve child's knowledge and skills. - For entertainment. - To keep child occupied. - For communication purposes. - To distract or divert child’s attention. - To put child to sleep.   Parents were asked to rate their concern (on a 5-point Likert scale) about perceived harmful effects of child’s use of digital media regarding:   - Poor sleep - Lack of physical exercise and play - Lack of parent-child interaction - Exposure to inappropriate content - Addiction - Poor eyesight |
| Vaala and Hornik, 2014, USA (111) | Mothers were asked to rate their attitudes (on a 7-point Likert scale) toward letting their child watch TV/videos for more than an hour a day on at least several days each week on three dimensions:   - bad/good - foolish/wise - harmful/beneficial |
| Vaiopoulou at al, 2021, Greece (112) | Parents were asked to rate their perspectives (on a 7-point Likert scale) of children using educational apps regarding the following themes:  Learning outcome:   - Facilitate new knowledge acquisition. - Enhance a child’s language development. - Promote creative thinking. - Contribute to the cognitive development. - Facilitate foreign languages learning. - Offer feedback in case of error. - Promote logical thinking.   Worries:   - Undermine children development. - They create health problems. - Problems are due to radiation. - Reduces quality interaction with parents. - Cause introversion in children.   Enjoyment:   - They offer pleasant sounds. - They enclose pleasant images. - They contain fun characters for kids. - They entertain the children.   Values:   - They are complemented by traditional teaching. - They offer multimedia teaching material. - They strengthen the motivation for learning. - They create an effective learning environment.   Usability:   - They provide instructions suitable for children of this age. - They are easy to use by children. - Children easily understand the content. - Children can use them without the guidance of an adult.   Involvement:   - They capture the child’s attention. - They capture the child’s interest. - They create an addiction to the child. |
| Vittrup et al, 2016, USA (113) | Parents were asked to rate their level of agreement (on a 7-point Likert scale) with the following statements regarding children’s use of media and technology:   - Media exposure at young age (0-3 years) is important for early brain development. - A child will not fall behind other children academically if his or her use of technology tools is restricted in the early years (0–5 years). - Children under the age of 2 years should have no TV screen time. - TV, videos, video games, or the computer can be used to keep children occupied while caregivers attend to important tasks. - Children under the age of 5 years should only watch educational programs. - TV advertisements have little impact on children under the age of 5 years. - The use of a computer can promote long-term physical, emotional, or intellectual developmental damage. - Introducing technological tools at a young age prepares children better for tomorrow’s work force. - Children today naturally understand how to use computers and related technologies at an early age. |
| Vincent et al, 2021, France (115) | No information was provided on how parents responded to the following items regarding which benefits they believed using smartphones and tablets had for children under the age of 3:   - Promotes learning (new words, improvement of language, shapes, colours, etc. - Develops dexterity (tactile function). - Calms, soothes, promotes concentration. - Keeps the child occupied/ Promotes sleep, helps to fall asleep. - Entertainment: pleasure, emotions, distraction, fun. - No perceived benefits.   No information was provided on how parents responded to the following items regarding which risks they believed using smartphones and tablets had for children under the age of 3:   - Negative impact on learning. - Negative impact on motor development. - Increases agitation, decreases attention or concentration. - Promotes isolation, reduces contact. - Prevents from sleeping well. - Increases the risk of overweight. - No perceived risk. |
| Wang et al, 2024, China (114) | Parents were asked to rate their level of agreement (on a 5-point Likert scale) with the following items:   - Children’s use of electronic information tools is not good for their brains. - Online activities (online games, movies) are detrimental to children’s development. |

*NR=Not Reported.*
