# Supplementary material for: Questionnaires Used to Explore the Perspectives of Parents and Health Professionals on Young Children’s Use of Technology: Systematic Review
Source: JMIR Pediatr Parent. 2026 Jun 11;9:e84712. doi: 10.2196/84712 (PMC13256481; doi:10.2196/84712)
Supplement: Multimedia Appendix 5 [file pediatrics-v9-e84712-s005.docx]

Table S1 presents full summaries of study findings related to perspectives towards children’s technology use based on questionnaire responses.

**Table S1.** Summary of findings related to perspectives towards children’s technology use based on questionnaire-responses.

| **Author, year, country** | **Perspectives from study participants** |
| --- | --- |
| Akyol, 2022, Turkey (31) | All parents included in the study stated that technology addiction was harmful (100%), and 35% of the parents thought that technology addiction increases in online education compared to face-to-face education. |
| Aladé and Donohue, 2022, USA (32) | A total of 67% of the parents agreed that having a mobile device in the home made their job as a parent easier; 82% agreed that that mobile devices help their children learn at home; 96% agreed that their child enjoys using a mobile device in the home; 85% agreed that they enjoy using the mobile device with their child; and 41% of the parents agreed that they were concerned that their child uses the mobile device too much.  When asked about mobile device use at school, 92% agreed that it is important for children to have access to technological in school; 38% agreed that technology use in school can distract from leaning; 94% agreed that there can be a healthy balance between traditional and technology-based teaching methods; 96% agreed that mobile devices can be a useful learning tool at school; while only 21% agreed that mobile devices takes aways from important learning in school. |
| Al-Balushi and Al-Shihi, 2016, Oman (33) | NR. |
| Alkalash et al, 2023, Saudi Arabia (34) | A total of 73% of the parents had a positive attitude towards their children’s exposure to screens. |
| AlSamhori et al, 2023, Jordan (35) | A total of 37% of parents believed that using technology improves their child’s mental and physiological growth, while 73% perceived that technology negatively impacts their child’s mental and psychological growth. |
| Amzalag, 2021, Isreal (36) | Parents perceived that their children gained the following skills through digital learning games: independent learning (mean on a 1-5 Likert scale=3.98, SD=0.96); curiosity, interest, and motivation (mean=3.69, SD=1.05); and creative thinking (mean=3.63, SD=1.21). Parent perceived that digital learning games used instead of traditional homework enabled their children to enjoy meaningful learning and to construct knowledge (mean=3.88, SD=1.18); to master learning materials (mean=3.80, SD=0.96); and to increase motivation and learn from their mistakes (mean=3.58, SD=1.09). |
| Arippin et al, 2023, Brunei Darussalam (37) | For items related to knowledge regarding their children’s screen time, parents most frequently agreed to the following statements: “Uncontrolled children’s screen time can lead to addiction to the devices” (46%); “children’s sleep pattern and quality can be disrupted by increase in their screen time” (39%); and “increase in children’s screen time is likely to decrease their effort in physical activity” (34%).  For items related to parents’ attitude towards their children’s screen time, parents most frequently agreed to the following statements: “I have the responsibility to control our child(ren)’s screen time by paying close attention on the appropriateness of the screen time activities” (90%); “I am aware that our child(ren) engagement with screen time is influenced by our use of screen-based devices and/or by others (e.g. siblings and/or friends)” (69%); and “I observed that our child(ren)’s use of screen device interferes with our family quality time” (66%).  For items related to parents’ practices around screen time, the most frequently agreed to statements were: “I ensure that I take away my child(ren) screen-based devices at home when they play or have social activities” (84%); “I encourage my child(ren) to play with toys or talk face-to-face rather than spending time every waking hour, using mobile phone, watching TV/video, and on laptop” (83%); and “I usually stop my child(ren)’s screen time at least an hour before bedtime to get him/her to fall asleep” (76%). |
| Asplund et al, 2015, USA (38) | A total of 34% of the parents disagreed that young children who never watch TV miss a lot that is of value, and 42% disagreed that TV is a useful way of keeping the children amused. |
| Balaban and Bayindir, 2019, Turkey (39) | NR. |
| Bansal et al, 2023, India (40) | For items related to knowledge regarding their children’s screen time, parents most frequently agreed to the following statements: “increased children screen time more likely to increase consumption of soft drinks and snacks”(32% mothers; 29% fathers); “children that spend more screen time are at risk of emotional, mental and behavioural problems“ (23% mothers; 25% fathers); and “increased children’s screen time may increase risk of the children being overweight/obesity” (22% mothers; 24% fathers).  For items related to parents’ attitude towards their children’s screen time, parents most frequently agreed to the following statements: “I have the responsibility to supervise our child(ren)’s screen time activity even when there is increase household and/or work demand” (22% mothers; 25% fathers); “it is challenging to manage our child(ren)’s screen time when there is a lot of screen-based  devices available in out household even though I should manage it” (21% mothers; 23% fathers); and “I would consider my child(ren)’s level of screen time to be a serious matter even if he/she/they is/are active, healthy and well-behaved” (21% mothers; 21% fathers).  For items related to parents’ practices around screen time, the most frequently agreed to statements were: “I try to limit or not use screen-based devices whenever I am with my child(ren)” (32% mothers; 33% fathers); “I don’t give screen-based devices to my child(ren) to keep them temporarily occupied and be quiet especially in time when I am busy and when he/she get fussy or moody” (28% mothers; 25% fathers); and “I usually stop my child(ren)’s screen time at least an hour before bedtime to get him/her fall asleep” (17% mothers; 20% fathers). |
| Barmomanesh et al, 2017, New Zealand (41) | A total of 48% of the parents had a negative feeling toward their child’s use of smartphones/tablets, whereas 31% were neutral, and 21% had a positive feeling. More than half (61%) of the parents believed that using digital technologies prepares children for the real world they have to live in. |
| Beyens and Eggermont, 2014, Belgium (42) | NR. |
| Bleakley et al, 2013, USA (43) | NR. |
| Boonmun et al, 2023, Thailand (44) | Results of parents’ attitudes towards their children’s screen time reduction were stratified in the control (n=35) and intervention group (n=36). In the control group, the mean attitude score was 72.86 (SD=8.41) at baseline, with higher scores indicating a more positive attitude towards children’s screen time reduction. In the intervention groups, the mean attitude score was 73.03 (SD=7.28) at baseline. |
| Bourha et al, 2024, Greece (45) | Prior to their child’s exposure to technology-enhanced toys, 50% of the parents believed that their child’s engagement with technology was very significant or extremely significant. Following their child’s exposure to such toys, this increased to 52.5%.  A total of 81% parents believed that technology-enhanced toys resulted in improvement of their child’s knowledge. Among these parents, 27% believed that their child’s fine-motor skills had improved; 25% believed that their child’s speech/vocabulary had improved; and 17% believed that their child’s creativity had improved after exposure to technology-enhanced toys. |
| Brauchli et al, 2023, Switzerland (46) | The following mean scores were reported regarding parents’ attitudes towards children’s screen media use across four measurement time points: T1=2.53 (SD=1.08); T2=2.51 (SD=1.06); T3=2.71 (SD=1.19); and T4=2.72 (SD=1.13) (with higher scores representing more positive attitudes). |
| Brown et al, 2023, USA (47) | The most commonly held beliefs about the negative effects of childhood digital media use were addiction to gaming/screens (52%), sleep problems (39%), getting out of shape or obesity (33%), loss of social skills or being impolite (33%), and exposure to violent or inappropriate content (32%). The most commonly held beliefs about the positive effects of childhood digital media use were exposure to a wider vocabulary (44%),%), learning about other cultures, places, and ideas (41%),%), and relaxation or fun (36%). |
| Cardy et al, 2023, Canada (48) | From the community sample, 44% of parents perceived a positive to very positive impact of technology use on children’s quality of life. Compared to the group of parents with autistic children, parents in the community group were more likely to report that their child has not benefited from technology in any area (adjusted odds ratio, AOR=0.35, 95%CI: 0.16, 0.73).  For children’s quality of life, more parents in the community group compared to the parents with autistic children believed that the impact of technology was positive for children who used technology for education (AOR=4.61, 95%CI: 2.52, 8.55), recreation (AOR=2.54, 95%CI: 1.37, 4.71), and therapy (AOR=8.50, 95%CI: 2.97, 30.78).  Leisure/recreation, language and communication, and cognitive development were the most common (over 40%) domains that parents from the community sample believed benefited children by using technology. |
| Carson et al, 2012, Canada (49) | NR. |
| Carson et al, 2013, Canada (50) | The most common perspectives among the parents were that screen time is enjoyable for their child (96%), when their child engages in screen time it gave the parents an opportunity to get things done (87%), and that screen time is a good learning tool (79%). The three most agreed on barriers to reducing screen time were that their child does not engage in excessive screen time (75%), screen time is enjoyable for their child (70%), and parents need time to perform household chores (64%). |
| Chattha et al, 2021, Pakistan (51) | A total of 45% of the parents believed that screen time improved the child’s knowledge and vocabulary; 14% of the parents believed that screen time negatively affected the child’s vision; 11% believed that the child learned unacceptable things when exposed to screens and 6% of the parents believe that screen time had no advantage for their children. |
| Chia et al, 2022, Singapore (52) | The most common positive perspective among the parents was that digital media was very important to improve their child’s knowledge and skills (48%). The most common concerns were that digital media use would result in addiction (76%), poor eyesight (73%), and expose the child to inappropriate content (74%). |
| Chen and Tu, 2018, Taiwan (53) | NR. |
| Cingel and Krcmar, 2013, USA (54) | Mean positive belief score among the parents was 3.77, SD=1.29, on a 1-7 Likert scale, with a higher score presenting more positive beliefs about children's media use. For the negative belief score, with a higher score presenting more negative beliefs about children's media use, the mean was 4.14, SD=1.39, also on a 1-7 Likert scale. |
| Covolo et al, 2021, Italy (55) | Most adults (predominantly parents) believed that the use of mobile devices by children aged 0-5 years old posed a health risk (74%) and that mobile devices were harmful to children (76%). The most commonly perceived risks associated with mobile device usage were eye irritation (83%), confusing virtual reality with real world (74%), and having sleep disorders (65%). The most commonly perceived benefit was communicating with distant relatives (47%). Becoming obese was considered a high risk by 51% and a low risk by 32% of respondents. |
| Dardanou et al, 2020, Norway, Portugal and Japan (56) | Among the three countries, the majority of the parents stated that using touchscreens entertained their children (mean = 0.47, SD = 0.50), followed by helped their children learn new knowledge (mean = 0.35, SD = 0.48), learn new skills (mean = 0.34, SD = 0.47); and in being creative (mean = 0.17, SD = 0.38).  Among the Portuguese parents, 50% expressed concerns about their children’s use of touchscreens, predominantly related to their children becoming dependent on technology and not socialising with their peers and environment. Furthermore, 82% of these parents believed that technologies do not enhance cognitive development for 0-3-year-olds, do not stimulate creativity, and that children’s health, specifically their vision, is affected negatively.  Among Norwegian parents, 50% expressed concerns about 0–3-year-olds using touchscreens, while 90% of Japanese parents stated concerns about their children’s touchscreen usage. A total of 29% of Norwegian parents and 12% of Japanese parents reported that early use of technology use would help children when they start at school. |
| Dong et al, 2022, China (57) | NR. |
| Eales et al, 2021, USA (58) | Parents’ perceptions of media use as helpful vs. hurtful were not different pre- to post-COVID-19. Mean score (with higher scores indicating ‘more hurtful’) pre COVID-19 was 3.19 (SD=0.51) and mean score post COVID-19 was 3.21 (SD=0.60). |
| Ebbeck et al, 2016, Singapore (59) | The majority of parents/caregivers identified that touch screen devices were most risky for children’s intellectual (55%) and physical (53%) development. Intellectual development consisted of risks related to addiction, undesirable contents and over-dependence. Physical development consisted of risks related to vision deterioration, inactive lifestyle and radiation. Most parents/caregivers (60%) believed touch screen devices may benefit children’s intellectual development. This included benefits related to improved academic outcomes, creative and interactive learning, and enhanced learning process. |
| Fan et al, 2022, China (60) | A total of 55% of the parents believed that short-video apps could broaden children’s horizons and help them understand the diversity of cultures and societies; 49% of the parents believed that short-video apps entertained their children and cultivating their personal hobbies; and 44% of the parents believed that short-video app would help their child learning language and life skills.  A total of 52% of the parents were concerned that their child had too much screen time, and 27% of the parents were concerned about unsafe content such as violence and bad language. |
| Farima et al, 2023, Moldova (61) | A total of 92% of the parents believed that the use of electronic devices could cause health problems in children; 26% of the parents believed that electronic devices contribute to the harmonious development of children; 17% believed that they have a beneficial effect on children; and 20% believed that devices help the child learn to read, write, and speak. |
| Garcia-Conde et al, 2020, Spain (62) | Mean score for parents believing that TV/screen is harmful for their child was 1.91 (SD = 1.02), with a higher score indicating parents’ belief that TV/screen is less harmful.  Mean score for parents believing that TV/screen is healthy entertainment for their child was 1.98 (SD = 0.90), with a higher score indicating parents’ belief that TV/screen is more entertaining. |
| Gjelaj et al, 2020, Kosovo (63) | A total of 67% of the parents reported positive attitudes towards using digital technologies for their young children.  The most common perceived benefits associated with children's technology use were language development (28%) and acquiring technology skills (25%). A total of 43% of the parents believed using technology would promote children’s school readiness.  When considering concerns about the negative effects of technology on their child’s development, 59% of the parents reported concerns about their child engaging in less active play time, 52% reported concerns about their child spending less time playing in the yard; and 36% reported concerns about developing physical disorders. |
| González-Sanmamed et al, 2023, Spain (64) | Overall, parents rated the risks associated with children using mobile devices higher than the benefits. With a higher scorer indicating higher perceived risk, the risks with the highest scores were “access to inappropriate content” (mean=4.12, SD=1.24), “commit/suffer cybercrimes” (mean=4.02, SD=1.28), and “addiction” (mean=3.95, SD=1.34). The perceived benefits with the highest scores were “entertainment” (mean=3.47, SD=1.14), “educational utility” (mean=3.45, SD=1.24), and “more access to information” (mean=3.38, SD=1.37). |
| Grané et al, 2023, Spain (65) | A total of 85% of the parents believed that some applications were educational; 70% believed that technology entertained children; and 63% believed that technology is fun for children to use. On the other hand, 78% of the parents believed that technology is additive for children and 46% believed that they are harmful for children under 6 years. |
| Griffith et al, 2023, USA (66) | NR. |
| Halpin et al, 2021, Australia (67) | Mean score for parents’ perception on how their child’s screen use affects their child’s health, development and daily functioning and their own wellbeing was 5.62 (SD=1.06, range=3.13-9.61), with higher scores indicating perceptions of more positive effects. |
| Hamilton et al, 2016, Australia (68) | The highest mean scores for the parents’ beliefs on benefits of children’s screen time were found for “promoting healthy habits” (mean=5.42, SD=1.57) and “improving my child’s social skills” (mean=5.27, SD=1.64), with higher scores indicating parents’ finding the benefit more likely.  The highest mean scores for the parents’ beliefs on costs of children’s screen time were found for “increase parent-child confrontations” (mean=4.01, SD=2.05) and “increase whining behaviour in my child” (mean=3.93, SD=1.99), with higher scores indicating parents’ finding the cost more likely. |
| Hatzigianni et al, 2014, Australia (69) | The most commonly held reasons among parents for why their child should use computers were to improve educational skills (40%), technological skills (38%) and to prepare their children for future employment (35%). |
| Howie et al, 2020, Australia & USA (70) | Parents reported the highest agreement with the statements “Using mobile technology will increase the amount of time my child spends sitting” (mean=4.3, SD=0.8) and “Using technology will reduce my child’s physical activity” (mean=4.0, SD=1.0).  Parents agreed with items on mobile technology being good for their child’s education (mean=3.4, SD=0.9), harming social skills (mean=3.2, SD=1.1), making their child at risk to online predators (mean=3.3, SD=1.1), and having overall benefits (mean=3.2, SD=0.9).  Parents disagreed with mobile technology being bad for education (mean=2.5, SD=0.8), being good for their child’s education when used in childcare (mean=2.6, SD=1.1), improving social skills (mean=2.2, SD=0.8), and being a risk overall (mean=2.8, SD=1.0). Parents were neutral (mean=3.0, SD=1.0) on mobile technology causing their child discomfort and pain. |
| Hutton et al, 2018, USA (71) | Results at first data collection (prenatal) showed that most mothers believed that television viewing by children <3 months old were “not important” (64%) or “somewhat important” (22%) for their development.  Results from the second data collection (~2 months old) were similar, with 53% of mothers believing television viewing was “not important” and 25% of mothers believed television viewing was “somewhat important” for healthy development. |
| Ihmeideh and Alkhawaldeh, 2017, Jordan (72) | For the physical or health aspect of the role of technology, parents scored a mean value of 3.52 (SD = 0.77) on enhancing children's movement skills and physical activity; 3.50 (SD = 0.67) on raising children's health awareness; and 3.75 (SD = 0.92) on increasing children's knowledge about safety procedures in their life.  For the intellectual aspect, parents scored a mean value of 4.17 (SD = 0.83) on developing children's thinking skills; 4.03 (SD = 0.77) on helping children develop creativity; 4.29 (SD = 0.82) on improving children's language and literacy; 4.18 (SD = 0.83) on developing children's problem-solving skills; and 4.05 (SD = 0.81) on developing children's numeracy skills.  For the emotional aspect, parents scored a mean value of 3.96 (SD = 0.78) on helping children in controlling and managing their emotions; 4.30 (SD = 0.80) on protecting children from fear, shyness, and anxiety; and 3.86 (SD = 0.95) on helping children sympathize with others and identify with community members.  For the social aspect, parents scored a mean value of 3.32 (SD = 0.82) on increasing children's awareness of their society and its problems; 3.45 (SD = 0.68) on helping children cultivate feelings of belonging and citizenship; 3.68 (SD = 0.91) on helping children communicate and interact with others; and 3.18 (SD = 0.74) on helping children know both, their social rights and duties.  For the moral aspect, parents scored a mean value of 3.52 (SD = 0.75) on helping children cultivate positive morals and values; 3.63 (SD = 0.96) on helping children exercise the highest ethics; 3.67 (SD = 0.88) on helping children distinguish between good and evil, right and wrong; and 3.94 (SD = 1.05) on helping children maintain the prevailing good practices and traditions.  For the religious aspect, parents scored a mean value of 4.01 (SD = 0.79) on developing children's positive attitudes toward their religious teachings; 4.14 (SD = 0.87) on helping children learn about certain religious duties; and 4.12 (SD = 0.88) on helping children acquire religiously inspired social etiquette.  For the aesthetic aspect, parents scored a mean value of 3.84 (SD = 0.92) on increasing children's ability to understand and appreciated the arts (acting, singing, theatre); 3.38 (SD = 0.92) on helping children tidiness, order and consistency; 4.14 (SD = 0.91) on helping children experience things and appreciate their beauty; and 3.62 (SD = 0.99) on helping discover and develop children's preferences, interests and skills. |
| Ilgar and Karakurt, 2018, Turkey (73) | More than 70% of the mothers believed that computer games had a negative impact on children’s dependency (93%) and socialisation (88%), and that computer games reduced time for family interaction (88%). Less than 50% of the mothers believed that computer games contributed positively to children’s perception, attention, and memory development (45%); creativity (37%); exploratory learning (31%); and self-production (22%). |
| Istenic et al, 2023, Slovenia (74) | Parents generally viewed traditional toys more favourably than digital screen toys across various developmental domains.  A total of 17% of the parents believed digital toys stimulated sensory development, and 94% believed traditional toys did the same. A total of 6% of the parents believed digital toys stimulate motor development, compared with 93% for traditional toys. Digital toys were perceived to promote cognitive development by 45% of the parents compared with 73% for traditional toys. A total of 15% believed digital toys enhanced emotional development, whereas 87% of the parents linked traditional toys to this. Digital toys were perceived by 43% of the parents as being able to encourage listening and observation, compared with traditional toys being perceived by 74% of the parents. Visual-spatial orientation was believed to be promoted by digital toys by 19% of the parents, whereas 84% believed this was stimulated with traditional toys. |
| Istenic et al, 2023b, Slovenia (75) | For parents' attitudes towards digital technology for child's development and leaning, parents rated the highest on the items of “I want the child to enjoy their childhood and not become addicted to technology” (mean = 4.48, SD = 0.73), “the use of a computer can promote long-term physical, emotional, or intellectual development damage” (mean = 4.15, SD = 0.90) and "my child should first learn to interact with the physical world, s/he has a lifetime to interact with the virtual world" (mean = 4.12, SD = 0.99). Parents would limit screen time to an hour daily (mean = 4.06, SD = 0.99), and they believed that screen technology could only be used under the supervision of parents (mean = 4.07, SD = 0.85).  Parents indicated concerns about the influence digital technology has on a child’s development and learning (mean = 3.79, SD = 1.01) and believed that a child develops better without digital technology (mean = 3.69, SD = 1.06). Parents believed that a child would not fall behind other children academically if his or her use of technology tools is restricted in the early years (0-3 years) (mean = 3.70, SD = 1.37).  For parents' perspective on child(ren)s play with traditional and digital toys, parents rated the highest on the items of "during playtime the most important is communication between a child and engaged person" (mean = 4.13, SD = 0.84); and "more important is communication between a child and person present than a toy itself" (mean = 3.99, SD = 0.91).  Parents believed that technology toys encourage children to be passive (mean = 3.38, SD = 1.00), preschoolers develop better without the technology (mean = 3.39, SD = 1.02); and that children under three should not use technological toys (mean = 3.64, SD = 1.06), but that some technological toys can enhance the child’s development of early literacy (mean = 3.45, SD = 0.82) and enable a child to learn how to manipulate technology (mean = 3.50, SD = 0.67). |
| Jain et al, 2023, India (76) | NR. |
| Jin, 2013, Korea (77) | Parents scored a mean value of 4.19 (SD = 0.58) on interest in children's internet use, with higher values representing higher agreement on the items. |
| Joginder Singh et al, 2021, Malaysia (78) | Most parents (67%) believed that screen time improved their children’s language skills, 7% believed that screen time had no effect on their children’s language skills, and 8% believed that screen time had a deteriorated effect. The remaining 18% of the parents were unsure of the effects of screen time on their children’s language skills. |
| Konok et al, 2020, Hungary (79) | A total of 32% of the parents reported a negative attitude towards child use of mobile touch screen devices, while 46% reported a neutral/mixed attitude, and 22% reported a positive attitude. Most parents believed that using a tablet/smartphone in early childhood can have both harmful (82%) and beneficial (77%) consequences. |
| Kostyrka-Allchorne et al, 2017, UK (80) | Regarding the severity of various features of television and film that were thought to be detrimental to young children’s development, violent content was rated as the most harmful compared to inappropriate language, inappropriate behaviour and fast pace (p<.001, 95%CI: -0.63 to -0.32; p<.001, 95%CI: -3.5 to -1.2; p<.001, 95%CI: 1.11-1.61, respectively).  Parents believed that the effects of popular media on children’s development were somewhat positive (p<.001, 95% CI: 0.83 to 1.22); the effects of watching fast-paced programmes were somewhat negative (p=.063); the effects of watching educational shows were positive (p<.001, 95%CI: 2.24 to 2.48); and the effects of watching violent content were very negative (p<.001, 95%CI: -1.82 to -1.44). |
| Lee et al, 2022, Korea (81) | Parents scored a mean value of 3.06 (SD = 0.65) on the positive attitude toward media use, with higher values representing more positive attitudes. For negative attitudes toward media use on intellectual and social dimensions of a child’s development, the parents scored mean values of 2.89 (SD = 0.73) and 2.83 (SD = 0.78), respectively. |
| Lepicnik et al, 2013, Slovenia (82) | When asked which competences the parents believed usage of information-communication technology (ICTs) mostly develops, 59% of the parents believed that ICTs partially develop child’s learning competences; 54% of the parents believed that ICTs partially develop a child’s motor competences; and 54% of the parents believed that ICTs partially develop child’s self-expression competences. A total of 49% of the parents believed that ICTs partially develop child’s language competences, and 42% of parents believed that ICTs partially develop child’s social competences. |
| Li and Chen, 2015, China (83) | Between 60% and 80% of the parents believed that tablets could make children ‘addicts’ and that tablets could ‘be prejudicial to health’. |
| Liibaan et al, 2023, Scotland (84) | Most parents believed that screen time has a negative impact on concentration (~40%), sleep (~40%), and social skills (~40%). |
| Little, 2019, UK (85) | NR. |
| Luo et al, 2023, Taiwan (86) | With higher scores indicating a higher level of agreement more positive attitude towards children’s technology use among the parents,  the highest means were found for the following statements: “rich audio and video help to improve interest in learning” (mean=3.62, SD=0.95); “provide a variety of learning content” (mean=3.50, SD=0.92); and “provide diverse stimuli for learning” (mean=3.44, SD=0.94).  Regarding negative attitudes towards children’s technology use, the highest scores were found for the following statements: “easily lead to the phenomenon of technology-use addiction” (mean=4.16, SD=0.87); “develop the habit of overreliance on audio-visual learning” (mean= 4.02, SD=0.83); and “affect the development of the optic nerve” (mean=3.94, SD=0.84). |
| Mansor et al, 2021, Malaysia (87) | NR. |
| Matziou et al, 2021, Greece (88) | A total of 48% of the parents did not agree that television affects their children, while 25% reported that television may affect children negatively. |
| Mikelic Preradovic et al, 2016, Croatia (89) | The positive attitudes of parents toward children’s computer use were indicated by their high level of agreement with the following statements: "children learn new and useful things on computer” (M = 3.72, SD = 0.99); “when using computers, children gain valuable IT skills which will be useful for them in the future” (M = 3. 98, SD = 0.91); and “computers can have only negative influence on children’s development” (M = 3.91, SD = 1.03) with the last statement being inversely coded.  The negative attitudes of parents were indicated by their high level of agreement with the following statements: “children who regularly use computer are at risk of developing dependence” (M = 2.26, 1.08); “children engage in sport less frequently due to the increased computer usage” (M = 2.37, SD = 1.31); and “excessive use of computers may separate children from their parents and friends” (M = 2.47, SD = 1.23) with all three statements being inversely coded. |
| Milford et al, 2022, Australia (90) | NR. |
| Nabi and Krcmar, 2016, USA (91) | NR. |
| Natsiopoulou et al, 2013, Greece (92) | A total of 83% of the high socioeconomic status (HSES) parents and 76% of the low socioeconomic status (LSES) parents agreed or totally agreed that using computers offers joy and entertainment to the child.  Among HSES parents, 56% agreed or totally agreed that using computers promotes participation in learning compared to 51% of the LSES parents. Computers were seen as enhancing imagination and creativity by 65% of HSES parents, compared to 49% of LSES parents who shared this view. Computers were considered to promote initiative by 43% of the HSES parents and 37% of the LSES parents agreed or totally agreed that using computers increase initiative. A total of 41% of the HSES parents agreed or totally agreed that using computers respects personal rhythms, while 47% of the LSES parents disagreed or totally disagreed. |
| Nikken, 2019, Netherlands (93) | Parents scored mean values of 3.48 (SD = 0.58), 3.00 (SD = 0.60) and 3.41 (SD = 0.58) on the positive effects of media on children’s learning, social skills, and emotions, respectively, with higher scores indicating more positive perspectives.  For the negative effect of media, parents scored mean values of 3.10 (SD = 0.80) and 2.95 (SD = 0.86) on children’s behaviour and health, respectively, with higher scores indicating more negative perspectives. |
| Nikken and Schols, 2015, Netherlands (94) | The “positive media effects” score of the parental attitudes about media for children had a mean value of 3.55 (SD = 0.65), while the “media function as a pacifier” score had a mean value of 3.04 (SD = 0.72), with a higher score representing a more positive perspective. |
| Njoroge et al, 2013, USA (95) | Parents of African American (OR = 4.16; 95% CI: 1.72-10.04) and Asian American/Pacific Islander/Hawaiian (OR: 2.73; 95% CI: 1.42-5.24) children had significantly higher odds of agreeing with the statement “educational TV programs can help preschoolers to play better with each other”, compared with parents of non-Hispanic white children.  Parents classified as high education/not low income had significantly lower odds of agreeing that “educational TV programs can help preschools learn to recognize letters and numbers” (OR = 0.52, 95% CI: 0.30-0.92); and the statement that “educational TV programs can help preschoolers play better with each other” (OR = 0.45, 95% CI: 0.30-0.69) compared with medium-education/not-low-income families. |
| Nwankwo et al, 2019, UK (96) | Majority of the parents (84%) were concerned that their children were spending a lot of time using screen devices, and 76% of them reported that their children were inactive due to this behaviour. Most parents (79%) agreed that screen time hinders and restricts their children from having a more active lifestyle. |
| O’Connor and Fotakopoulou, 2016, UK (97) | Most of the parents (72%) perceived learning new skills as the benefits of their 0-3-year-olds using touch screens, while 52% and 39% of the parents perceived learning new knowledge and allowing them to be creative as the benefits of the use of touch screens, respectively.  A total of 62% of the parents reported having concerns about their 0-3-year-olds using touch screens, and 26% of the parents expressed fears that their child may become addicted to their mobile devices. |
| Ophir et al, 2023, Isreal (98) | In study 1, the mothers had a mean of 3.04 (SD=0.83),), with higher scores indicating a more positive attitude towards children’s screen use. For the negative attitude score, the mean was 3.61 (SD=0.80),), with higher scores indicating a more negative attitude towards children’s screen use.  In study 2, the mothers had a mean of 2.68 (SD=0.77) for the positive attitude score. For the negative attitude score, the mean was 4.10 (SD=0.63). |
| Petegem et al, 2019, Belgium (99) | Parents had a mean score of 2.54 (SD=0.66) for negative attitudes toward children’s digital gaming, with higher scores representing a more negative attitude. |
| Raj et al, 2022, Malaysia (100) | A total of 37% of the parents had a negative attitude towards children’s screen time, while 63% had a positive attitude. The scores of parents’ perception regarding the influence of screen time on their child’s well-being were 8.0 ± 4.0 on physical wellbeing, 10.0 ± 4.0 on cognitive wellbeing and 6.0 ± 3.0 on social wellbeing, with one unit increase in parental perception score indicating greater perception of positive influence of screen time on child’s wellbeing. |
| Raj et al, 2023, Malaysia (101) | With higher scores indicating a more positive perception of screen time on child’s well-being, mothers in both the intervention and control groups had a mean score of 2.7 (SD=0.5-0.6) at baseline. |
| Rajalakshmi et al, 2023, India (102) | The most commonly held beliefs among caregivers were that digital media might cause behaviour problems (68%), sleeping problems (55%); and increase the knowledge of children (54%). Overall, 30% of the caregivers perceived that digital media has no positive effects and 11% of caregivers perceived that it has no negative effects. |
| Rosanda et al, 2022, Slovenia (103) | Parents mostly believed that digital toys with and without screens facilitated cognitive development (46%) and listening skills (41%).  Of positive effects of digital technology, the most commonly held beliefs among the parents were that it encourages development of basic mathematical skills (33.3%), problem-solving skills (22%), basic reading skills (17%), and promotes hand-eye coordination (17%). |
| Sada Garibay and Lapierre, 2024, Mexico (104) | The mean score of parent perceived risk in their children’s use of video streaming was 2.46 (SD=1.01), on a four-point scale with higher scores indicating stronger risk perceptions regarding media. |
| Seršen et al, 2024, Slovenia (105) | The majority of the parents (94%) perceived that children’s programmes can have a positive contribution to their child’s development and learning. For emotional development, parents perceived positive effects as fun and happiness while watching (n=35), relaxation and comfort before going to bed (n=22), and getting acquainted with emotions, expressing emotions and empathy (n=18). For imagination, creativity and thinking, parents perceived positive effects as development and stimulation of the imagination (n=32), encouraging curiosity and exploration (n=21), development and encouragement of creativity (n=8), and stimulating thinking and logical thinking (n=8). For social development, parents perceived positive effects as learning how to solve conflicts and problems (n=7), learning what is good and what is bad (n=6), and learning about interpersonal relationships and developing social skills (n=8).  A total of 42% of parents responded that they don’t have any concern, while 58% of the parents expressed concern.  For emotional development, parents were concerned about the development of screen addiction (n=20), and emotional and behavioural issues (n=11). For cognitive development, parents were concerned about attention deficiency (n=7). For social development, parents were concerned about copying improper behaviour, bad habits or unreal images (n=12). |
| Solomon-Moore et al, 2017, UK (106) | Parents had a mean score of 3.8 (SD=0.8) for negative attitudes toward children’s screen viewing, with higher scores representing a more negative attitude. |
| Stuckelman et al, 2023, USA (107) | NR. |
| Suresh and Tiwari, 2023, India (108) | Among parents of typically developed (TD) children, 50% reported having a neutral attitude towards their child’s media technology and screen time use. Regarding the attitudes related to screen based devices use, 59% agreed or strongly agreed that screen-based devices prevent their children from interacting with others; 48% agreed or strongly agreed that their children are benefiting from screen based devices; and 40% disagreed or strongly disagreed that screen-based device usage has negatively affected their children’s communication skills.  Regarding the concerns related to screen based device use, 71 % agreed or strongly agreed that increased use of screen-based devices can result in a social deficit, less social interaction and isolation of their children, 66% agreed or strongly agree that increased used of screen based devices can negatively affect child’s creativity and imagination; and 64% agreed or strongly agreed that excessive screen-based devices can impair their children’s communication skills. |
| Tanusha et al, 2023, Malaysia (109) | The overall parents’ perception regarding digital device use among their preschool children was mixed, with 30% perceived more benefits, 35% perceived more harm, and 35% were unsure of its effects.  For the perception of risk, most parents perceived that digital device use would cause risk to their children’s physical (72-91%) and intellectual domains (72-86%). Majority of parents perceived that digital device causes damage to eyesight (91%), results in device addiction (86%) and exposes their children to radiation (81%).  For the perception of benefits, over half of parents perceived that digital device use promotes creative and interactive learning (63%), promotes technology awareness (65%) and is easily accessible and portable (63%). |
| Tay et al, 2021, Singapore (110) | Parents considered digital media use most important for improving the child’s knowledge and for entertainment, with mean ratings of 3.13 and 3.38, respectively, with higher scores representing more important. Parents were most concerned about perceived harmful effects of digital media use as poor eyesight (mean = 4.08) and addiction (mean = 4.07), followed by lack of parental-child interaction (mean = 3.70), lack of physical exercise and play (mean = 3.49) and poor sleep (mean = 3.19), with higher scores representing more concerned. |
| Vaala and Hornik, 2014, USA (111) | Mothers had a mean score of 3.93 (SD = 1.51) for attitudes towards letting their child watch more than an hour of TV/videos a day for at least several days each week, with higher values indicating more positive attitudes. |
| Vaiopoulou at al, 2021, Greece (112) | Parents in the “Negative Attitude” group, who did not appreciate educational apps and worried about their children’s use, were more likely to be in the younger age group (b = -0.075, p < 0.001).  Parents with higher knowledge of new technologies and who appreciated apps were most likely to belong to the “Positive Attitude” group (b = 0.41, p < 0.01).  Parents in the “Negative Attitude” group believed that app causes conflict between parents and children to a higher degree (b =0.61, p < 0.0001) compared to parents in the “Mild Attitude”, “Positive Attitude” and “Indifferent Attitude” groups. |
| Vittrup et al, 2016, USA (113) | The media attitude score ranged from 17 to 41, with a mean of 28.82 (standard deviation (SD) = 4.74), showing a positive skew (i.e. positive perspectives).  A total of 33% of parents agreed that media exposure at a young age (0–3 years) is important for early brain development, and 33% believed that children may fall behind other children academically if their use of technological tools is restricted in early childhood.  Most parents (93%) believed that TV advertisements do influence young children; 11% believed that the use of computers can promote long-term physical, emotional, or intellectual developmental damage. A total of 69% of parents believed that introducing technological tools at a young age helps prepare children better for tomorrow’s work force. |
| Vincent et al, 2021, France (115) | A total of 35% of parents believed that using smartphones and tablets had benefits for children’s learning; 17% believed that the use helped develop dexterity; and 4% believed that the use calms, soothes and promotes concentration.  In terms of perceived risk associated with using smartphones and tablets for children, 72% believed it caused isolation, 65% believed it caused restlessness, and 62% of parents believed it caused sleep disturbance. |
| Wang et al, 2024, China (114) | With higher scores indicating a higher level of agreement, the mean scores for the statement “children’s use of electronic information tools is not good for their brains” were 2.890 (SD=1.120), 2.630 (SD=0.916) and 2.830 (SD=1.108) for mothers, fathers and grandparents, respectively. Mean scores for the statement “online activities (online games, movies) are detrimental to children’s development” were 3.190 (SD=1.242), 2.880 (SD=1.246) and 3.230 (SD=1.198) for mothers, fathers and grandparents, respectively. |

*SD=Standard Deviation.*
