# Supplementary figures and images for: Questionnaires Used to Explore the Perspectives of Parents and Health Professionals on Young Children’s Use of Technology: Systematic Review
Source: JMIR Pediatr Parent. 2026 Jun 11;9:e84712. doi: 10.2196/84712 (PMC13256481; doi:10.2196/84712)

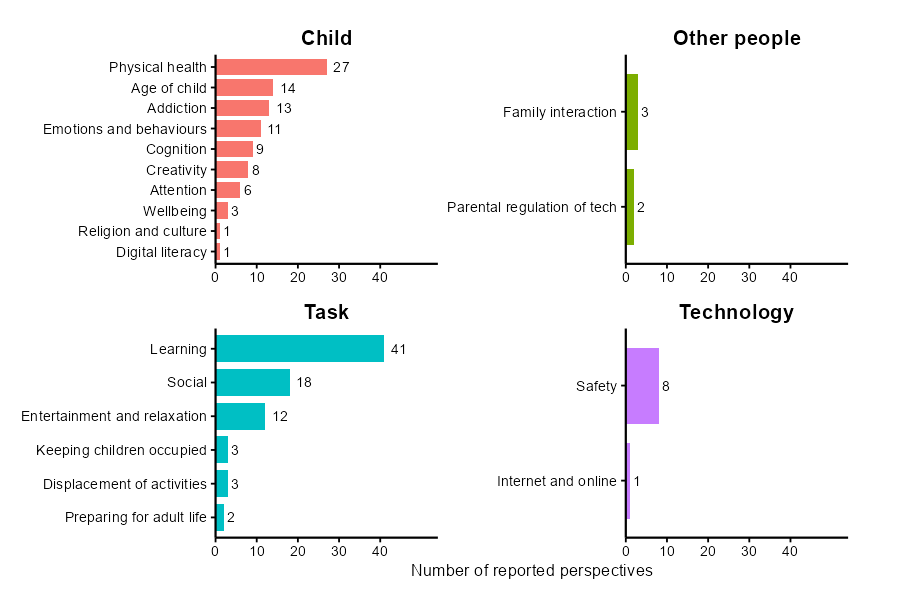

Supplement: Multimedia Appendix 6 [file pediatrics-v9-e84712-s006.png]
